# Examining Challenges to Co-Design Digital Health Interventions With End Users: Systematic Review

**DOI:** 10.2196/50178

**Published:** 2025-03-14

**Authors:** Anthony Duffy, Nazanin Boroumandzad, Alfredo Lopez Sherman, Gregory Christie, Indira Riadi, Sylvain Moreno

**Affiliations:** 1 School of Interactive Arts & Technology Simon Fraser University Surrey, BC Canada; 2 Meta Seattle, WA United States; 3 School of Gerontology Simon Fraser Universiity Vancouver, BC Canada

**Keywords:** digital health, end users, user experience, health behavior, intervention, co-design, mobile health, mHealth, digital health citizen

## Abstract

**Background:**

Digital health interventions (DHIs) are changing the dynamic of health care by providing personalized, private, and instantaneous solutions to end users. However, the explosion of digital health has been fraught with challenges. The approach to co-design with end users varies across a diverse domain of stakeholders, often resulting in siloed approaches with no clear consensus. The concept of validating user experiences contrasts greatly between digital stakeholders (ie, user experience and retention) and health stakeholders (ie, safety and efficacy). Several methodologies and frameworks are being implemented to address this challenge to varying degrees of success.

**Objective:**

We aimed to broadly examine the advancements and challenges to co-design DHIs with end users over the last decade. This task was undertaken to identify the key problem areas at the domain level, with the ultimate goal of creating recommendations for better approaches to co-design DHIs with end users.

**Methods:**

We conducted a systematic search of key databases for co-design studies involving end users in DHIs. Searches were divided into 3 relevant streams: health behavior, user experience, and digital methodologies and frameworks. The eligibility criteria were guided by the PerSPEcTiF framework and the PRISMA (Preferred Reporting Items for Systematic Reviews and Meta-Analyses) checklist. In line with this framework, studies were included in this review that (1) address research on DHIs; (2) focus on interaction and co-design with end users; (3) explain results such that uptake, effectiveness, satisfaction, and health outcomes are discernible, positively or negatively; and (4) describe actionable procedures for better DHI design. The search was conducted in a diverse group of 6 bibliographical databases from January 2015 to May 2024: PsycINFO, PubMed (MEDLINE), Web of Science, CINAHL, Institute of Electrical and Electronics Engineers Xplore, and Scopus. From the 13,961 studies initially screened for titles and abstracts, 489 (3.6%) were eligible for a full-text screening, of which 171 (1.2%) studies matched the inclusion criteria and were included in a qualitative synthesis.

**Results:**

Of the 171 studies analyzed across 52 journals, we found 5 different research approaches, spanning 8 different digital health solution types and 5 different design methodologies. These studies identified several core themes when co-designing with end users: advancements, which included participatory co-design; challenges, which included participatory co-design, environment and context, testing, and cost and scale; and gaps, which included a pragmatic hybridized framework and industry implementability.

**Conclusions:**

This research supports a pragmatic shift toward using mixed methods approaches at scale, methods that are primed to take advantage of the emerging big data era of digital health co-design. This organic outlook should blend the vision of digital health co-designers with the pragmatism of Agile design methodology and the rigor of health care metrics.

**Trial Registration:**

PROSPERO CRD42021238164; https://www.crd.york.ac.uk/prospero/display_record.php?ID=CRD42021238164

**International Registered Report Identifier (IRRID):**

RR2-10.2196/28083

## Introduction

### Background

Digital health interventions (DHIs) are revolutionizing health care delivery by providing personalized, private, and instantaneous solutions to end users. An umbrella term, a DHI is a mobile- or web-based digital solution that facilitates a diverse array of health interventions—be they emotional, decisional, or behavioral. A DHI is designed for a complex set of end users—those who use the intervention, including patients, caregivers, and health care professionals [1]. With the ever-increasing ubiquity of mobile health (mHealth) technologies, cost-effective scalable DHIs are emerging almost daily [2]. These solutions range from self-monitoring to diagnostic, educational, and behavior change–based interventions. At the heart of a DHI is the contextual adaptation [3] around the needs of end users, those who use the technology directly to impact their health [4], such as a patient with diabetes monitoring blood sugar on an mHealth app. This is an exciting yet challenging shift in health care design. It promises more autonomy to the end user through a bottom-up [5] design approach; however, it ruptures the top-down [6] traditional health care approach that positions experts as the central designers of interventions. This evolution marks a shift from visiting health facilities for health services toward the ubiquitous digital participation of end users and health experts. This has produced a new era of health literacy built around diagnostics, services, and self-help. This reciprocal, empowering approach is creating a more mutual understanding [7], evolving the dynamic [8] among health care professionals, engineers, designers, and end users in DHI co-design, ultimately shifting the balance of power toward the patient. Nonetheless, the promise of DHIs is tempered by the fact that 26% of apps are discarded after first use [9]. To address this concern, numerous design approaches such as user-centered and human-centered design, among others, are being used to varying degrees of success [10]. Each approach seeks to incorporate end users, from the primary stages of ideation to the final testing stages of production, gathered around multidisciplinary teams of digital and health care professionals. It is within this focus that we have examined the prominent plus points (ie, advancements), pain points (ie, challenges), and system-wide gaps to incorporate end users in co-design, issues that cut across the entire range of design approaches used in digital health at present.

### The Complexity of Hybridizing Digital and Health Approaches to Incorporate End Users

Despite today’s digital sphere increasingly being one of digital natives, the path toward a fluid, organic digital health design space has been fraught with challenges. From a digital perspective, rapid Agile development methods evolve to garner end-user satisfaction in an evolving form of iterative qualitative evaluation. From a health care perspective, slow, safe expert-led studies produce large quantitative datasets toward validating patient involvement [11]. Digital health, in its nascent stages, sits somewhat uncomfortably between these two domains. From the macrolevel of policy down to the microlevel of co-design workshops, the very *language of collaboration* may differ. Hybridizing these 2 contrarian methodologies is challenging. The concept of building validation around the user or patient varies, depending on the given perspective. These challenges, along with others, have led to a multiplicity of design approaches, a disparity in conceptualizing the end user, and competing agendas [6,7,12,13] among DHI stakeholders that muddy the waters of digital health design. In this regard, we were inspired to conduct this systematic review on the basis of the initial analysis of our previous study titled, “The Challenges Toward Real-world Implementation of Digital Health Design Approaches” [10]. In our primary study, we found that irrespective of the domain, design methodology, or user definition (Multimedia Appendix 1), there are common pain points (ie, repeated points of conflict or displeasure resulting in a poor user experience [UX]) emerging that impact digital health at the domain level.

In addition, numerous studies [3,6,11,14,15] have noted the challenge to design frameworks that effectively define and implement “co-design” or “co-leadership” around end-user involvement in DHI design. On one hand, health stakeholders may lament the lack of alignment of UX design to health care delivery [9]; on the other hand, digital stakeholders may insist that user-centered design (UCD) is essential toward acceptability [16]. In both cases, each stakeholder is viewing *efficacy* through a different lens. This cuts to the heart of the matter of co-designing DHIs with end users; the overarching philosophy and methods used are starkly different. Therefore, there is a need to better understand the common pain points in this collaboration, which in turn may lead to a better perspective on why, when, and how to incorporate end users in the design of DHIs.

### Objective of This Study

This study seeks to compile the core challenges to incorporate end users in the design of DHIs over the last decade. We sought to thematically analyze current approaches, to analyze evidence of pain points that presented a challenge or conflict in the incorporation of end users in the co-design process, and to explore gaps and opportunities toward a more organic co-design approach in digital health. This includes a broad stroke of studies, including quantitative, qualitative, mixed methods, and research through design. It features the perspectives of health care, psychology, engineering, development, and design in the production of mHealth, eHealth, and extended reality DHIs. This intentionally broad approach is reflective of the nascency of digital health as a field and the open-mindedness of this study, such that the divergence and convergence of methodologies and tools is viewed as a prosperous and necessary growing pain toward a more organic digital health approach to end users.

It is hoped that through critical analysis of these advancements, challenges, and gaps, synthesized findings from the following questions lead to an improved approach toward incorporating end users in DHI co-design:

1. How are end users currently incorporated into iterative DHI design, and what is the effectiveness of current methods?
2. What are the most common pain points (ie, challenges) encountered while incorporating end users in DHI design activities?
3. What are the current gaps in end-user research at the domain level, and how can these be addressed to better integrate end users into the iterative design process in digital health, aiming to improve efficacy and uptake?

## Methods

### Study Design

This systematic review followed an a priori published protocol with detailed methods registered with PROSPERO (CRD42021238164) [17]. The review followed the Cochrane Handbook for Systematic Reviews [18] and was reported using the PRISMA-P (Preferred Reporting Items for Systematic Reviews and Meta-Analyses Protocols) guidelines [19].

We followed six stages in this systematic review: (1) literature search, (2) article selection, (3) data extraction, (4) quality appraisal, (5) data analysis, and (6) data synthesis.

### Eligibility Criteria

Considering the complexity of DHIs, we used the PerSPEcTiF [20] guidelines for intervention (Table 1). We selected it due to its suitability for qualitative synthesis in the health care domain.

In line with this framework, studies were included in this review that (1) address research on DHIs; (2) focus on interaction and co-design with end users; (3) explain results such that uptake, effectiveness, satisfaction, and health outcomes are discernible, positively or negatively; and (4) describe actionable procedures for better DHI co-design.

**Table 1:** Details of the PerSPEcTiF question formulation framework as applied to this review.

Initials	Definition	Detail
Per	Perspective	From the perspective of end users
S	Setting	In the setting of digital health
P	Phenomenon of interest or problem	What are the most prominent pain points
E	Environment	Within an environment of designing DHIs^a^
C	Comparison (optional)	—^b^
Ti	Time	During ideation and co-designing
F	Findings	In relation to understanding the challenges of successfully incorporating end users in the successful design of DHIs

^a^DHI: digital health intervention.

^b^Not applicable.

### Search Strategy

We systematically searched 6 electronic databases: PsycINFO, PubMed (MEDLINE), Web of Science, CINAHL, Institute of Electrical and Electronics Engineers Xplore, and Scopus. We selected these databases according to preliminary searches and consultations with experts and librarians in this field. Keywords related to DHIs from preliminary searches were compiled and refined. We adapted the search strategy as needed to return a breadth of papers without retrieving an unmanageably large number of irrelevant articles.

A list of the search terms used in this review were grouped into 3 clusters (Table 2). In addition, we have included all search strings for replicability (Multimedia Appendix 2).

**Table 2:** Clusters of categorized search keywords.

Categories	Keywords
DHIs^a^	“digital health” or “mHealth” or “eHealth” and “end user*” or “end user*”
User experience	“user experience” or “UX” or “user centred” or “user centered” or “human centred” or “human centered” or “patient centred” or “patient centered” or “person based” or “person centered” or “person centred” or “participatory design” or “involvement” and “digital health” or “mHealth” or “eHealth”
Methodologies and frameworks	“agile” or “scrum” or “kanban” and “digital health” or “mHealth” or “eHealth”

^a^DHI: digital health intervention.

Cluster 1 includes DHIs (owing to behavior change in health care resulting from DHIs). Cluster 2 includes UX (owing to engineering, development, and design in digital health). Cluster 3 includes methodologies and frameworks (owing to digital project facilitation and to health care intervention design and policy).

### Inclusion and Exclusion Criteria

The inclusion and exclusion criteria were based on the peer-reviewed studies outlined in Textbox 1.

**Inclusion and exclusion criteria.**

**Inclusion criteria**

- Qualitative studies
- Case studies
- Observational studies, including cross-sectional surveys
- Nonrandomized studies (eg, before and after studies and interrupted time series studies)
- Cohort studies

**Exclusion criteria**

- Noncritical analysis of end-user involvement in digital health design (ie, no results or discussion on end-user incorporation)
- Single person studies
- Nonprimary research (eg, reviews and commentaries)
- Randomized controlled trials
- Nonhuman studies

Due to the rapid innovation of digital health, only studies in the English language from January 1, 2015, to May 31, 2024, were included. Broadly put, the inclusion criteria included any population group that involved the critical analysis of end-user incorporation (ie, inclusive of terms such as patient, person, and human) in the co-design of a DHI. Studies that did not meet this criteria were excluded, including those that included end users but did not provide any critical analysis of the process; reviews and commentaries due to them being nonobservational or nonexperimental; single-user studies due to the inability to generalize; randomized controlled trials (RCTs) due to a lack of focus on the end-user integration in the design of a DHI; and nonhuman studies, which are impertinent to the design of a DHI.

### Screening and Article Selection

All articles identified and selected from database searches were stored in the reference management software Paperpile (Paperpile LLC), which was used to eliminate duplicates and to tag and organize the research findings. The initial screening process involved 2 independent reviewers (AD and either IR or NB) who screened titles and abstracts of all studies for possible inclusion using an Excel (Microsoft Corp) spreadsheet (Multimedia Appendices 3 and 4) to ensure no studies met the exclusion criteria. After filtering results on the basis of the possible inclusion criteria, the second stage involved a full-text review of the remaining articles, which was conducted by 3 independent reviewers (AD and IR or AS). Upon scrutiny of the full-text selections, reviewers (AD and IR or AS) removed items that did not meet the inclusion criteria. In the third and final stage, reviewers (AD and IR or AS) met to cross-reference eligibility of the final inclusion list, as well as to settle any conflicts. This cross-reference involved the individual review of all items to white list them per the inclusion criteria. For items in conflict, reviewers (AD and IR or AS) simultaneously scanned items side by side to determine whether the items met the inclusion criteria. Since this was the third round of filtering, the majority of items met the inclusion criteria, so the determining factor in conflicts was to collectively review each item to see whether the studies actively involved end users in the design of the DHI and whether critical analysis of this was present in the results or discussion sections of the respective study. This final step removed any subjectivity and resolved conflict.

### Data Extraction, Analysis, and Synthesis

A data extraction template was created by one of the authors (AD) that provided a record of the study characteristics. The categorization of themes and subthemes emerged organically from the findings (Multimedia Appendix 5). The collection, extraction, and subsequent analysis and synthesis of data is outlined in Table 3.

Raw content (ie, quotes and reflective analysis) was extracted and collected in a research document (Multimedia Appendix 6). Data were extracted and analyzed relative to the goals of the research questions, following guidance from the Cochrane Handbook for Systematic Reviews of Interventions [18]. These data are synthesized and presented in narrative form in the Results section. Full characteristics of all included end-user studies are available in Multimedia Appendix 7.

**Table 3:** Data extraction characteristics and the identified themes and subthemes.

Category				Description
**Data extraction**				
	Summary of characteristics			Full citation, author, PMID^a^, year, and journal
	Study type			Case study, quantitative study, qualitative study, research article, and mixed methods study
	Study details			Target population, end users, duration (h), and solution
	Design methodology			User-centered design, human-centered design, patient-centered design, person-based approach, patient-led design, and general participatory design.
	Project facilitation method			Kanban, Scrumban, Waterfall, XP^b^, Agile inspired, design thinking, and unspecified
**Data analysis and synthesis (themes and subthemes)**				
	**Key plus points (ie, positives)**			
		Participatory co-design	Collaboration, ideation, improved mode of delivery, behavior modification, and in situ environment	
	**Key pain points (ie, negatives)**			
		Participatory co-design	Collaboration, the language of digital health, co-design, competing interests, hierarchical versus democratic design, and methodological misalignment	
		Environment and context	Untested in situ and multiplicity of user types	
		Testing	Lack of participants, quality of participants, condensed time frame, lack of comprehension, technology, and cultural sensitivity	
	**Cost and scale**			
		Challenges (ie, gaps in the research)	Organic co-design approaches, fluidity of digital health efficacy evaluation, Agile digital health methods, industry implementability, supply chain, medical device versus consumer app, and research and development limitations	

^a^PMID: PubMed identifier.

^b^XP: extreme programming.

## Results

### Search Results

**Figure 1:**
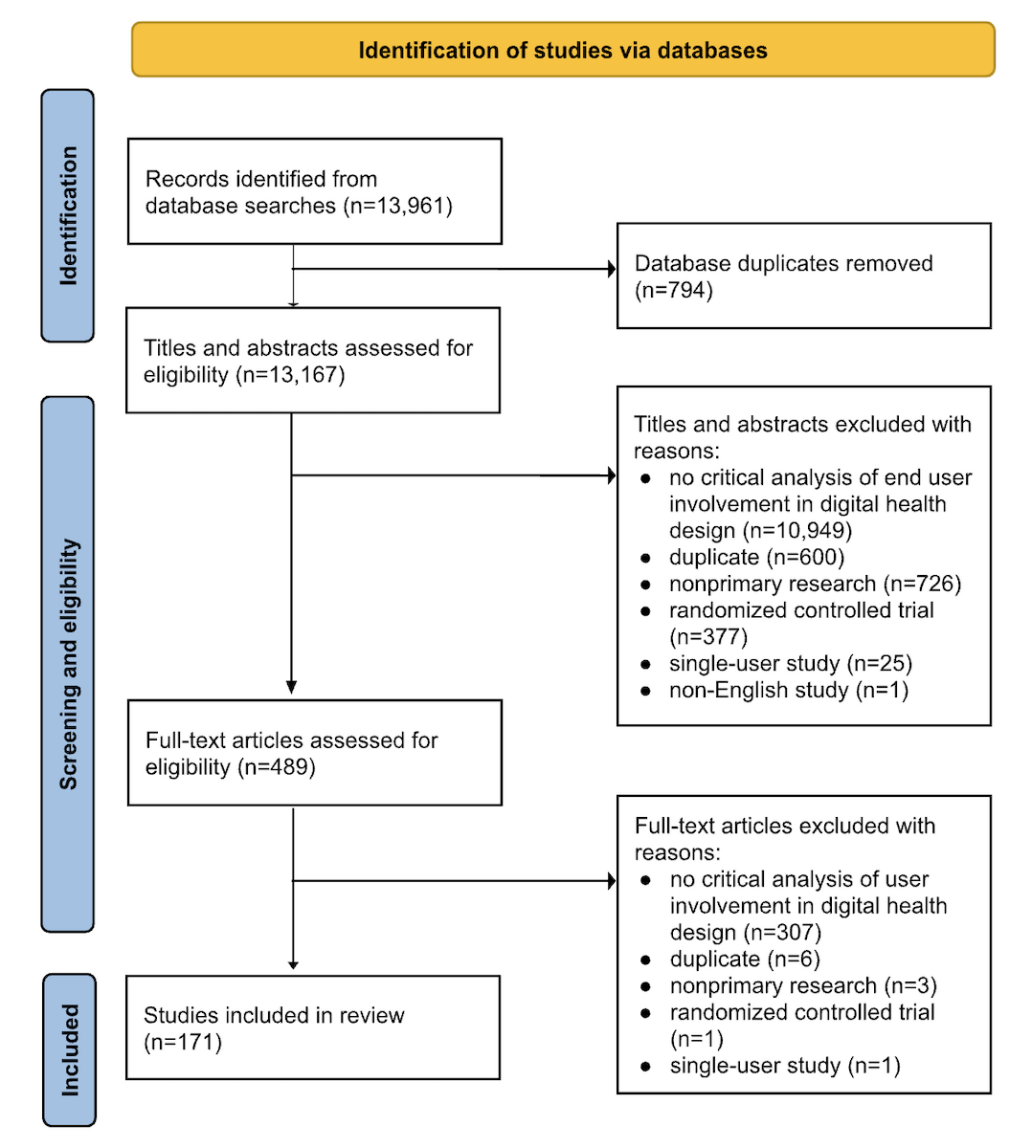
PRISMA (Preferred Reporting Items for Systematic Reviews and Meta-Analyses) flowchart of the study inclusion and exclusion process.

The search strategy identified 13,961 studies. After removing duplicates (n=794), a total of 13,167 (94%) studies were screened for title and abstract. After the initial title and abstract screening phase, 12,678 (91%) studies were excluded, leaving 489 (4%) potentially relevant papers. In the second full-text screening phase, 318 (2%) studies were excluded. This resulted in the inclusion of 171 (1%) relevant studies in this systematic review. Reasons for exclusion included a lack of critical analysis of end-user involvement in digital health co-design and nonprimary research (eg, reviews and op-eds, RCTs, and single-user case studies). A PRISMA (Preferred Reporting Items for Systematic Reviews and Meta-Analyses) flow diagram (Figure 1) was used to record the details of the screening and selection process so that the study can be reproduced.

### Study Characteristics

Before addressing our research questions, we present a record of characteristics detailing the diversity of digital health studies retrieved (Table 4).

**Table 4:** Analysis of study characteristics (N=171).

Study characteristics			Studies, n (%)			
**Year**						
		2015	4 (2.3)			
		2016	7 (4.1)			
		2017	14 (8.2)			
		2018	18 (10.5)			
		2019	25 (14.6)			
		2020	38 (22.2)			
		2021	10 (5.8)			
		2022	26 (15.2)			
		2023	24 (14)			
		2024	5 (2.9)			
**Type and solution**						
	Qualitative			107 (62.6)		
	Quantitative			6 (3.5)		
	Mixed methods			43 (25.1)		
	Case study			12 (7)		
	Research article			4 (2.3)		
**Methodology and methods (project facilitation^a^)**						
	Agile			24 (14)		
	Scrum			9 (5.3)		
	Design thinking			10 (5.8)		
	Unspecified			133 (77.8)		
**Target population and end users**						
	Patients			91 (53.2)		
	Older adults			23 (13.4)		
	Health professionals			15 (8.8)		
	Young people			11 (6.4)		
	Adults			9 (5.3)		
	Patients and health professionals			5 (2.9)		
	Other			17 (9.9)		
**Journal type**						
	JMIR			94 (55)		
	BMC^b^			11 (6.4)		
	IEEE^c^			6 (3.5)		
	Other			60 (35.1)		
**Digital solution**						
	mHealth^d^			97 (56.7)		
	Web based			35 (20.5)		
	eHealth			22 (12.9)		
	Device			7 (4.1)		
	Serious gaming			6 (3.5)		
	Other			4 (2.4)		
**Design methodology^e^**						
	User-centered design			84 (49.1)		
	Participatory design			58 (33.9)		
	Human-centered design			18 (10.5)		
	Person-centered design			9 (5.3)		
	Person-based approach			4 (2.3)		
	Person-led design			1 (0.6)		
**Number of end users involved**						
	1-5			21 (12.3)		
	6-10			44 (25.7)		
	11-15			28 (16.4)		
	16-20			15 (8.8)		
	21-30			25 (14.6)		
	31-40			7 (4.1)		
	41-50			6 (3.5)		
	≥51			9 (5.3)		
	Unspecified			16 (9.4)		

^a^Three studies identified with Scrum, which is a subset of Agile.

^b^BMC: Biomed Central.

^c^IEEE: Institute of Electrical and Electronics Engineers.

^d^mHealth: mobile health.

^e^Three studies identified more than one methodology.

### Study Types

Owing to the diversity of digital health, the 171 studies identified span 5 different research approaches. Most approaches were qualitative (107/171, 62.6%), followed by mixed methods (43/171, 25.1%), indicative of the social and emotional influence on DHIs. These studies were collected from 52 different journals across multiple research domains, with JMIR accounting for the majority (94/171, 55%).

### Study Details

The DHIs spanned 8 different solution types with mHealth (97/171, 56.7%) representing the majority. This development is in line with the growing dominance of mobile digital devices with up to 92% of internet traffic at present being mobile based [21].

These solutions were used in 15 different populations with patient-focused populations being the most prominent (91/171, 53.2%). There was considerable variation in the number of end users involved in a DHI co-design with 6 to10 (44/171, 25.7%) end users being the most prominent, followed by 11 to15 (28/171, 16.4%), 21 to 30 (25/171, 14.6%), and 1 to 5 (21/171, 12.3%) end users. Only 9 studies (5.3%) involved >50 end users.

### Study Design Methodology

As a guiding design methodology, UCD (84/171, 49.1%) was most prominent, followed by the broader approach of participatory design (58/171, 33.9%), then human-centered design (18/171, 10.5%). Other approaches such as patient-centered design, patient-based approach, and patient-led design were used sparingly.

### Study Project Facilitation Method

Finally, in addition to tracking the design methodology, we tracked the project facilitation method used. The vast majority of studies did not specify a project facilitation method (133/171, 77.8%) that was used. Of those that did, Agile was used the most (24/171, 14%), followed by design thinking (10/171, 5.8%). Scrum, a subset of Agile, was next (9/171, 5.3%), while none specified other common industry methods such as Kanban, Extreme Programming, or Waterfall. This is particularly interesting when comparing how digital health prototypes are facilitated in academic research environments versus typical industry methods.

**Figure 2:**
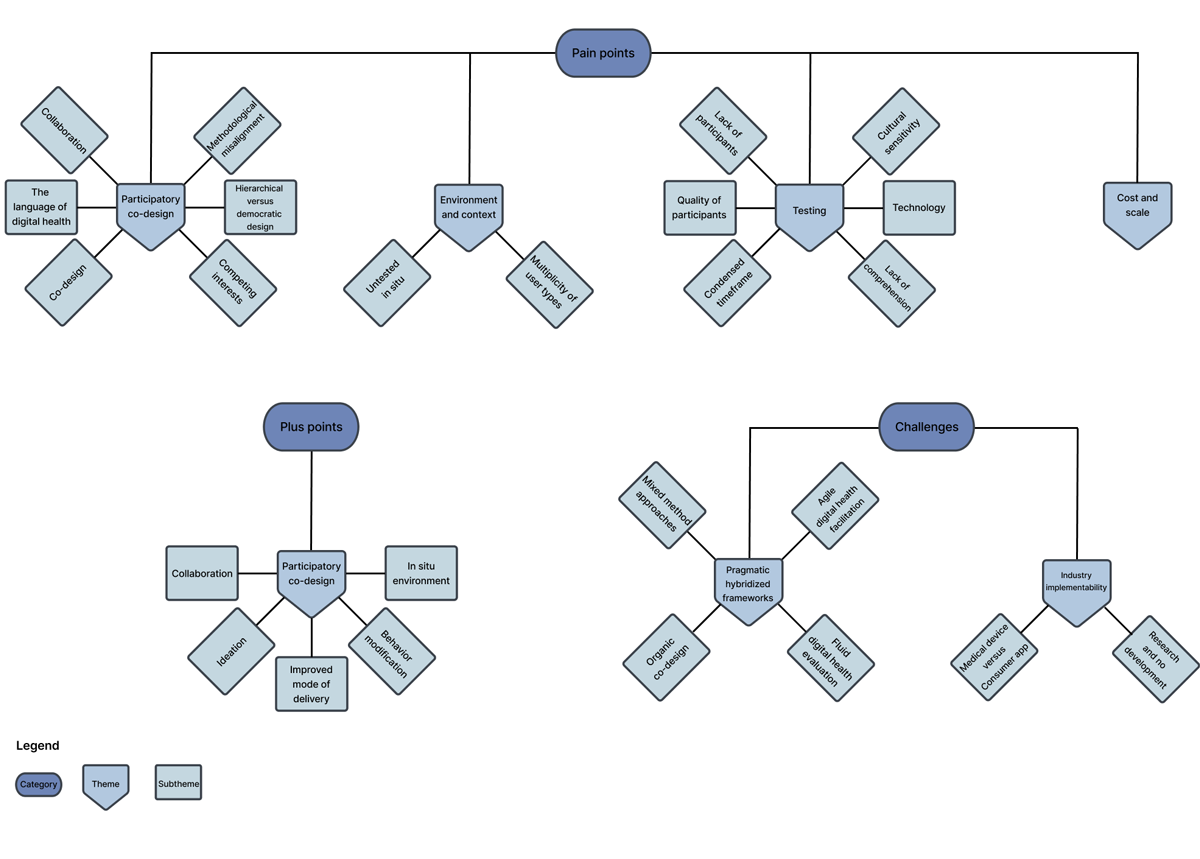
Flowchart of the thematic analysis of plus points (ie, advantages), pain points (ie, disadvantages), and challenges to co-design digital health interventions (DHIs) with end users.

We present the aforementioned study characteristics to illustrate the diversity of investigating the incorporation of end users in co-design. As discussed, this rigorous approach sought to incorporate as many vantage points as possible to identify common advancements, challenges, and opportunities for improvement in the domain. A thematic analysis of the plus points (ie, advantages), pain points (ie, disadvantages), and challenges to incorporate end users in the co-design of DHIs (Figure 2) as addressed in the 3 research questions is presented later.

A brief definition of the resultant themes and subthemes is presented in Table 5. This is followed by an explanation of the results relevant to each of the 3 research questions in this study.

**Table 5:** Description of the themes and subthemes identified in the data synthesis.

Section and theme						Description			Studies, n (%)
**Plus points**									
	**Theme: Participatory co-design**				Positive advancements from the active participation of end users in the co-design workshop phases to ideate and test DHIs^a^			95 (55.5)	
		**Subthemes**							
			Collaboration	Collaboration among stakeholders (eg, developers, health professionals, and end users) in the co-design of a DHI			69 (72.6)		
			Ideation	Creative process to construct ideas, requirements, and design concepts in the nascent stages of co-design with end users			55 (57.9)		
			Immediate behavior modification	The benefits of instant behavior changes obtained by end users through the knowledge enhancing collaboration of DHI design workshops			16 (16.8)		
			In situ environment	Ideating and testing with end users in the actual environment the intervention is designed for (ie, hospital, care homes, among others)			7 (7.4)		
**Pain points**									
	**Theme: Participatory co-design**				Failures when incorporating end users in the active participation of co-design workshop phases to ideate and test DHIs			70 (40.9)	
		**Subthemes**							
			Collaboration challenges	Failures to curate and implement collaboration approaches among stakeholders (eg, developers, health professionals and end users) in the co-design of a DHI			14 (20)		
			The language of digital health	Difficulties communicating a common language of co-design considering the various semantics that stem from differences in collaborator perspectives (ie, digital, health care, and end users)			14 (20)		
			Cultural sensitivity	Understanding the nuances of culturally appropriate approaches that are used to describe the content and functionality of DHIs with end users			13 (18.6)		
			Competing interests	Contrasting objectives defined by health and digital goals that create friction in the process of designing around the end users			18 (25.7)		
			Methodological misalignment	Differences in digital health intervention design stemming from methodological misalignment (ie, digital, health or behavioral approaches to co-design)			13 (18.6)		
			Hierarchical versus democratic design	The challenging contrast between the slow, steady, and data-driven approach to health care design and the laissez-faire rapid approach to digital design innovation, making processes and evaluation difficult in digital health			17 (24.3)		
	**Theme: Environment and context**				The collaboration and testing environment of the end user and the contextual understanding of who the end user is			38 (22.2)	
		**Subthemes**							
			Untested in situ	The lack of co-designing or testing in the contextual environment of the user			23 (60.5)		
			Multiplicity of user types	Lack of understanding of the complexity of user types (ie, patient, person, clinician, therapist, and caregiver)			16 (42.1)		
	**Theme: testing**				Problems incurred during the testing phase with end-user collaborators that led to questions on the validity, reliability, and generalizability of results			113 (66.1)	
		**Subthemes**							
			Lack of participants	In reference to a lack of end-user testers leading to questions on the validity and reliability of results and determinations			75 (66.4)		
			Quality of participants	In reference to a lack of broader representation of user types (ie, patient user, caregiver user, and clinician user), including demographic concerns (ie, ethnic diversity), thus weakening the generalizability of results			58 (51.3)		
			Condensed timeframe and limited participation	The condensed time frame leading to limited participation of end users in collaboration workshops and testing, raising questions on rigor and applicability of results broadly			25 (22.1)		
			Lack of technological comprehension	Inclusive of knowledge of technical terminology and digital device variance (ie, iPhone vs Android)			14 (12.4)		
**Challenges**									
	**Theme: Pragmatic hybridized frameworks**				The need for new frameworks that are friction reducing, pragmatic (ie, fit for purpose), and hybridized (ie, a collective fluid combination of digital, health and behavioral objectives), leading toward more transdisciplinary approaches in DHI design			60 (35.1)	
		**Subthemes**							
			The value of mixed method approaches to end-user data	Despite the advantages that mixed methods approaches bring to DHIs (ie, blending emotional and functional feedback for better design decisions), the uptake in mixed methods approaches to data collection is low and inhibits more pragmatic approaches to DHI evaluation			24 (40)		
			Organic co-design approaches	The challenge to cocreate approaches to end-user involvement in co-design that are not a sum-of-its-parts approach; siloing digital (ie, usability) and health care (ie, safety) elevation as unrelated metrics			25 (41.7)		
			Fluid digital health efficacy evaluation	The challenge to amalgamate the *fail fast* approach of digital design and the *first do no harm* approach of health care design around end-user design involvement			39 (65)		
			Agile digital health project facilitation	The difficulty to implement Agile industry-styled co-design practices with the rigor and requirements of health care safety and efficacy			18 (30)		
	**Theme: Industry implementability**				The lack of consideration when co-designing with end users for downstream industry implementation within the complexity of the health care system (ie, government policy and industry constraints and regulations that impact design)			13 (7.6)	
		**Subthemes**							
			Medical device vs consumer app	The challenge to navigate consumer (ie, end user) preferences that collide with medical device regulation			5 (38.5)		
			Supply chain	Health care delivery involves the collaboration of industry suppliers and government regulatory bodies that bring DHIs to the end users. The lack of health care business analysts in the co-design process with end users negates business modeling factors that could reduce downstream deployment challenges.			6 (46.2)		
			Research and no development	The lack of recognition and resolution of the aforementioned failures and challenges often leads to prototypes that never reach the development or deployment stages			5 (38.5)		

^a^DHI: digital health intervention.

### Research Question 1: How Are End Users Currently Incorporated Into Iterative DHI Co-Design, and What Is the Effectiveness of Current Methods?

#### Theme: Participatory Co-Design

The most positive and influential approach to the incorporation of end users in the co-design of DHIs can broadly be termed as participatory co-design (ie, an approach wherein end users participate in the co-design process). In total, 95 of the 171 (55.5%) included studies discussed the advantages and advancements of participatory co-design in the digital health domain, irrespective of the domain approach (ie, engineering and health among others). This overarching methodological approach culminated in subthemes such as collaboration, ideation, environment, and improved behavior modification.

##### Subtheme: Collaboration

Collaboration (69/171, 40.4%) [6,7,9,12,22-87] was identified as a key benefit of participatory co-design. Studies explained that collaboration in DHI design allows complex teams to co-create dynamic solutions [30] that uniquely combine a variety of digital and health approaches around the end user [23,88,89]. Collaboration may take the form of interviews, design workshops, and crowdsourcing (ie, web-based surveys and mass testing). This constructive, creative, iterative [90], and engaging process reflects the diverse mosaic of digital health stakeholders, while increasing the understanding of end-user needs due to the variety of vantage points [91]. Increasingly, studies showed that collaborating with a diverse stakeholder group around the end user, from ideation to production, increased end-user design participation [8,28,36], improving evidence-based approaches [92]. This development is increasingly important, since earlier attempts to incorporate end users in late-production testing have been widely found to be ineffective [22]. Collaboration, particularly through the early low-fidelity stages of prototype design, increased adaptability of the solution in a low-cost phase [36]. It also created mutual understanding among all stakeholders regarding their roles and how they relate to the DHI as a sum of its parts rather than as siloed experts [37,93]. This approach is increasing sensitivity to the cultural context and personal preferences of end users, which in turn is improving the common language of communication in digital health solutions [32]. In short, collaboration is the element of participatory co-design that is closing the gap between the patient, clinician, and designer [39].

##### Subtheme: Ideation

Ideation (55/171, 32.2%) was identified as a key benefit in participatory co-design. Loosely defined as the ability to generate new ideas [30], studies used it as a hands-on approach to incorporate users within digital health teams in workshops [29] to cocreate and prioritize end-user preferences [30,94] and subsequent features in a DHI. This process was used early in the DHI design process, often encapsulated in design sprints or health game jams [95] that empathetically seek to understand end-user needs in a process of divergence and convergence [14,38], whereby finding differences is seen as a necessary building block on the path to agreeing on a robust co-designed solution.

##### Subtheme: Immediate Behavior Modification

Immediate behavior modification (16/171, 9%) [15,24,27,39,42,51,64,68,69,74,76,96-98] in participating end users was identified as a beneficial yet perhaps unexpected advantage of in situ collaboration and ideation. Studies observed instant behavior modification from incorporating end users in DHI workshops. The multidisciplinary nature of rapid iteration that organically collects design, development, health care, and end-user experience data in a decentralized experimental environment allowed for persuasive design and behavior change in real time. This reciprocal process allowed digital and health stakeholders to educate end users on how a DHI can improve their experience, while end users were able to educate other stakeholders on how and what makes a positive UX for them.

##### Subtheme: In Situ Environment

Co-designing in situ (7/171, 4%) [35,53,72,89,91,99,100] such that on-site collaboration with end users took place in hospitals, clinical environments, or homes [89] increased the realism of stakeholder participation, leading to a more natural evaluation of the intervention. In such studies, this led to a better ability to co-conceptualize solutions [101]. This shift is in response to studies showing limitations on the quality of data and the potential effectiveness of DHIs that were commonly not tested in situ (eg, university laboratory environments), which challenges external validity [35,100]. In situ co-design increased familiarity of surroundings, enhanced comfort, and provided a more genuine, meaningful interaction with end users [91].

### Research Question 2: What Are the Most Common Pain Points (That Is, Challenges) Encountered While Incorporating End Users in DHI Design Activities?

#### Theme: Participatory Co-Design

Participatory co-design was simultaneously a core theme of the positive advancements as well as common failures to incorporate end users in the design of DHIs (70/171, 40.9%). This dichotomy is not unexpected considering the nascency of the burgeoning digital health industry. These challenges are related to collaboration, communication (ie, the language of digital health), competing interests, hierarchical versus democratic design approaches, and methodological misalignment. Broadly speaking, these challenges are growing pains toward blending digital and health care approaches in co-design.

##### Subtheme: Collaboration Challenges

Collaboration challenges (14/171, 8%) [5,7,30,41,53,65,68,71,88,89,102-105] in participatory co-design ranged from the challenge to curate collaboration approaches based on the given use-case scenario, to variance in stakeholders’ knowledge bases (ie, technology, health care, and UX), to miscalculating the need for onboarding co-designers in the workshop process, to mediating disagreements [102], and misallocating stakeholder types (ie, too many clinicians, not enough end users, or vice versa) [103]. These factors led to disagreements, increased timelines, and overbudgeting that challenged the effectiveness of the co-design around the end user. These collaboration challenges are resultant of a clash of cultures (eg, digital and health care) and are defined by the following subthemes.

##### Subtheme: The Language of Digital Health Co-Design

Challenges to form a common language in co-design (14/171, 8%) [42,60,62,64,65,71,74-76,103,106-109] identified the difficulty of blending the semantics of health care, digital design, and the colloquialisms of end users. Design “sprints” [108] sometimes felt rushed to health care stakeholders and end users if unexplained. While safety may indicate imminent danger to end users, in health care, safety is more indicative of preventing harm from happening [107]. Conceptualizing what a prototype was or explaining what a minimum viable product represents in nascent design stages was not a given for nondigital stakeholders. Usability (ie, technical), UX (ie, emotional), and patient efficacy (ie, medical) are all by-products of the co-design process, but the language, interpretation, and implementation in the workshop meant different things to different stakeholders leading to conflicting understandings.

##### Subtheme: Cultural Sensitivity

Stemming from the common language issue, studies identified the challenge to collaborate with end users in a way that is culturally sensitive and appropriate (13/171, 7.6%) [38,39,42,44,47,57,68,88,110-115]). Cultural sensitivity has been observed in 4 main categories: content, functionality, technology, and user interface [3]. Studies cited the challenge of using inclusive digital media in workshops that is culturally befitting, as well as addressing sensory needs (eg, vision and hearing deficiencies) of patient end users [88]. For example, the use of gamified incentives and rewards in mHealth apps [38] for communities that suffer from gambling addictions may be a critical oversight. Studies that incorporated cultural frameworks built with end users showed promise toward negating downstream challenges in the design cycle, particularly concerning appropriate content, features, and imagery in the interface design [3,39].

##### Subtheme: Competing Interests

Competing interests (18/171, 10.5%) [4,7,9,14,16,43,64,78,88,98,101-103,106,116-119] were found to be a prominent challenge within participatory co-design with end users. This can be summarized as the focus on health systems and services versus the needs of matching digital technologies to user preferences [14]. These competing lenses [101] led to contrasting perspectives on how to incorporate end users in DHI co-design. Broadly, it speaks to the clash of cultures as expert-led health care approaches and innovative user-driven approaches collide. The concept of “shared ownership” proved uncomfortable in numerous studies [9,98,101,118]. Although there was a shared vision on improving quality and safety around end users, exactly *how* this was achieved is a point of conflict [7]. Disease management, improved well-being, and UX are all facets of an intelligent design, yet they speak to different objectives [95]. Stakeholders often felt internal pressure to deliver aspects of the solution that reflect their domain (ie, digital, health care, and policy), which challenged the co- in co-design. Where a patient stakeholder may desire anonymous data tracking (eg, for privacy), a health care professional may desire mapped data tracking (eg, for safety and electronic records), and a digital stakeholder may need analytics (eg, to drive sales) [7]. The inability to corral these competing interests led to disengagement or abandonment of the collaboration in some cases [119].

##### Subtheme: Methodological Misalignment

Ironically, a paradox exists in that the emergence of diverse stakeholder groups seeking to co-design with end users has led to difficulties in communicating and co-designing [120]. Methodological misalignment (13/171, 7.6%) [29,35,47,58,60,64,65,69,91,120-122] in the approach to evaluating safety, usability, and efficacy differed vastly between health care and digital experts, respectively. The goals of these contrasting stakeholders were quite divergent [35]. In this regard, digital health is a domain struggling to formulate its own unique design culture around end users. Contextual barriers [91,121] exist such that the qualitative nature of rapid design sprints does not quantify the risks, safety, or efficacy of a longitudinal study, which spans large datasets (ie, RCTs). Simply put, it can be difficult to gain buy-in due to the vast variation in research approaches.

##### Subtheme: Hierarchical Versus Democratic Design

Methodological misalignment surfaced most prominently as a conflict between hierarchical and democratic concepts of designing DHIs (17/171, 9.94%) [3,6,9,12,15,35,38,69,90,93,113,123-128]. Traditionally, a more paternalistic relationship [3] exists between health care experts and patients (ie, end users). This hierarchical construct often relies on expert-driven “hard data” decisions. This is contrary to the more democratic, inclusive approach to digital design workshops, where the end user and other stakeholders are often seen as equal. The concept of *leadership and followership*, such that design workshops often see different stakeholders take the lead based on a given exercise [93], is a new and rupturing approach to health care interventions. Evidence-based and experience-based approaches accentuated the juggling act of balancing perspectives and soothing egos. On the design side, certain biases related to the pragmatic mode of using user-driven exercises conflicted with evidence-based research in health care, especially in the arena of safety and clinical efficacy. Design workshop approaches to user personas and user journeys tell a story about a user and their preferences, but they do not account for clinical perspectives on harm prevention and safety. Where digital experts may insist “the solution must occur organically” around the end user, this democratic approach may be viewed as oversimplification by health experts who may insist that a “first do no harm” approach must involve quantitative data that exemplifies safety and efficacy. In this context, the aforementioned challenges culminate in the battle between innovation and clinical reality [15].

#### Theme: Environment and Contextualization

After understanding the various challenges that participatory co-design poses to stakeholders working with end users in the design of a DHI, an emergent theme that developed was the impact of the collaboration environment and the contextualization of the end user (38/171, 22.2%).

##### Subtheme: Untested In Situ

Numerous studies observed the challenging impact of not designing a DHI with end users in situ (23/171, 13.4%) [3,7,15,30,68,73,74,77,99,106,110,113,116,129-138]. In situ here refers to co-designing or testing in the contextual environment of the user. For example, a triage digital tablet in a waiting room or a sleep diary app in a user’s bedroom. This limited generalizability, challenged the value [110] of design workshops, and weakened the ability to evaluate the true context of use [3,116,129-131,134-137]. Without testing DHIs in their natural environment, the context of the data collected was sometimes considered incomplete. Furthermore, it does not observe how end users would interact with the data and experiences they are creating, *in real-world settings* [30,106,133]. For many mHealth solutions, app use was found to vary by time and location, with various environmental factors impacting how end users may interact with the intervention [7,132]. This made it increasingly difficult to generalize both design considerations and testing conclusions from a controlled laboratory environment. For digital stakeholders, this may render UX evaluation speculative. For health stakeholders, clinical efficacy evaluation may be incomplete.

##### Subtheme: Multiplicity of User Types

Another factor impacting the contextualization of the DHI was the multiplicity of user types (16/171, 9.4%) [6,47,54,58,72-74,77,80,85,130,137,139-142]. End users were viewed as patients, persons, clinicians, therapists, and passive users (ie, an observer such as a caregiver), among others. Numerous DHIs had more than one end user, each using the solution for a different objective. Unlike traditional collaborative design workshops that involve 1 end-user type or a potential customer, mHealth solutions often provide services to numerous types of end users. For instance, in heart medication apps, a patient may objectify a knowledge base with push notifications and, as a user goal, a therapist may objectify behavior change as a user goal and a physician may objectify metrics to track medication history as a user goal—all 3 stakeholders may be end users of the same DHI [137]. Therefore, positioning the patient as the sole end user may insufficiently reflect the scope of end-user incorporation in the design process.

#### Theme: Testing

Foundational challenges in participatory co-design, inclusive of environmental and contextual issues, unsurprisingly culminated with pain points downstream in testing with end users (113/171, 66.1%). Testing subthemes included a lack of participants, the quality of participants, a lack of testing comprehension, and condensed time frames.

##### Subtheme: Lack of Participants

A lack of participants (ie, end users) challenged the generalizability of results (75/171, 43.8%) [3,7,11,15,25,27,28,32,34,36,41,42,45-49,51-57, 60,61,63-66,69,71,72,74,75,80,84,90,94,98,99,104, 105,108,110,119,127,129,131-134,136,140,142-163]. This limitation in the number of participants is a common challenge for co-design workshops [28]. The reliance on 5 test participants [11,131,157] is a common practice in qualitative research, where findings from 5 end users are often assumed to reflect the experiences of 50 or 500 end users. This approach is foreign to traditionally quantifiable health research, and is admittedly speculative and potentially biased, as observed in many of the study limitations. Therefore, despite the richness in emotional feedback derived from and around end users in co-design workshops, the lack of quantifiable evidence limits and weakens the broad applicability of results [129]. Terms such as “sufficient” [8] or “generally provable” were used alongside admissions of lack of generalizability. The rapid, cost-effective, human-centered approach [15] to design workshops concedes larger sample sizes, edge cases, and an overall breadth of data obtained from large-scale patient trials in health care. Ironically, while aiming to use methods that address “the needs of end users,” [32,34,147,156,157,164,165] these same methods inherently reduce the scope of end user needs, conceding large-scale feedback that could further advocate for design workshops emphasizing “acceptability” and “usability,” with large-scale, quantifiable validation from a broader pool of end users.

##### Subtheme: Lack of Diversity of Participants

The lack of diversity among participants (58/171, 33.9%) [12,14,30,35,36,38,41,42,45-49,51-57,61,63-66,88, 95,98-100,104,105,111,124,130,139,145,148,152,155,159,161,166-169] was evident in a third of studies. Studies with small sample sizes were not always able to account for various cultural and educational user differences within a demographic [14,30,88,95,100,111,127,145,155]. The lack of representability, both in variety of end-user types (eg, patient, clinician, caregiver, and others) and skillset (ie, tech savvy superusers [124] vs standard users) contributed to consistent validity and generalizability concerns. With the clinician (ie, patient advisor), patient, and caregiver (ie, observer) often being co-users of the DHI, excluding one end-user group or more from the co-design or testing phases was seen as a risk of biasing results [29,139,168]. The user test of a patient and that of a clinician may vary drastically due to contrary perspectives and user needs, despite them both being end users of the same DHI [35,36,148].

##### Subtheme: Condensed Time Frame and Limited Participation

In some cases, the quantity and quality of participants was directly impacted by the rapid, condensed time frame (25/171, 14.6%) [14,30,35,37,53,60,64-66,71,80,83,92,99,100, 108,116,119,146,148,149,152,170-172] of co-design workshops built around end users. Some studies recognized that despite the overtly flexible approach to iterating with end users, involving them alongside clinicians and digital stakeholders, from conception to validation, is a time-constrained process affected by availability and budget [14,30,100,108,148,149,152,171]. The challenge to coordinate health stakeholders’ involvement in end-user co-design was more apparent, considering that design workshops are not a part of the day-to-day workflow of health professionals [170]. In addition, patient health often dictated availability and digital teams often floated between multiple projects simultaneously [116]. The short time frames often resulted in only low-fidelity prototypes with a limited scope of input, often making it difficult to speculate whether higher fidelity finished products would garner the same feedback [119].

##### Subtheme: Lack of Technological Comprehension

The previously mentioned pain point on the language of digital health co-design translated to further challenges downstream when testing ideas and prototypes due to a lack of technological comprehension (14/171, 8.2%) [14,23,27,32,36,71,74-76,116,144,145,173] among some stakeholders. Assumptions connecting personal experiences and digital health solutions were sometimes unvalidated, creating a disconnect among stakeholders [14,173]. In addition, familiarity with testing devices (eg, iPhone vs Android or extended reality interfaces) [144] was not ubiquitous. The lack of perception and clarity of objectives sometimes intimidated end users [36] and increased the difficulty in blending clinical, usability, and UX feedback in a constrained timeframe.

In summary, participatory co-design with DHI end users has highlighted challenges on the difficulty of amalgamating common interests, common language, and the collision of systematic and organic design approaches, culminating in methodological misalignments that produce varying testing approaches and hoped outcomes. This challenges the broader goal of defining a digital health framework around end-user involvement in co-design.

### Research Question 3: What Are the Current Gaps in End-User Research at the Domain Level, and How Can These Be Addressed to Better Integrate End Users Into the Iterative Design Process in Digital Health, Aiming to Improve Efficacy and Uptake?

After assessing plus points and pain points in the first 2 research questions, our final question sought to understand the limitations and gaps that are challenging the advancement of end-user incorporation in the design of DHIs.

#### Theme: Pragmatic Hybridized Framework

Despite positive advancements in incorporating end users in digital health co-design, the various aforementioned flash points led to advocating for a more pragmatic hybridized digital health framework (60/171, 35%). Subthemes centered around the need for a more mixed method, adaptable, and contextual approach to incorporating end users; one that is a fluid, whole-of-system digital health approach.

##### Subtheme: The Value of Mixed-Method Approaches to End-User Data

Numerous studies identified taking a more mixed-method approach (2/171, 14%) [26,36,40,48,50,52,57,66,72,80,81, 96,104,138,141,154,155,161,174-178] as a cornerstone toward hybridizing digital and health approaches to incorporate end users. Unsurprisingly, mixed methods studies suffered less limitations in the form of validity and generalizability. Notably, studies found that there were discrepancies between what end users verbalized versus what was being observed from them [36], indicative of a mismatch between end-user desires and end-user actions. This observation strengthened the need for other forms of quantitative assessment for correlation. Another study found that pooling larger datasets through the System Usability Scale [26,141] and weighing them against smaller qualitative datasets was seen as a more prudent method of testing assumptions and weighing feedback. A study by He et al [176] found that end-user preferences for a tutorial (documented in qualitative interviews) was implemented, but they were subsequently found to be ignored by end users during quantitative data use analysis of the next iteration. Gaining metrics from hypothesis testing [37] was seen as an emotionally detached compliment to qualitative workshop approaches to gain personal insights from end-user preferences and behavior. An additional benefit to this approach was comparing data between experiment and control groups [149]. In some cases, the “hard data” metrics from mHealth app use were used to support or reject findings from interviews and observations. This was strongly exemplified in a study by Fico et al [37] in which a user-centered workshop design (ie, a qualitative approach) was complemented by bio-signal sensors to implement “a reliable information workflow.” In this regard, valuable emotional feedback from end users was cross-referenced with sensory data analytics for stronger conclusions [148,177]. In essence, the pragmatic, synthesized coevaluation of quantitative and qualitative data may only serve to strengthen the reliability of studies by blending quantifiable statistics with emotional insights from end-user analysis.

##### Subtheme: Organic Co-Design Approaches

Building upon the value of richer mixed method approaches to evaluating end-user co-design involvement, the need for more organic co-design approaches (25/171, 14.6%) [3,9,16,38,40,46,47,50,52,57-59,61,64,65,68,81,82,89,106,116,126,141,177] was discussed in a number of studies. Organic, as in a holistic co-design approach, one that combines digital, health, and behavioral metrics into a more unified process. This is a response to the problem of misaligning usability and health care design objectives and the recognition that “end users” are not one single homogenous group [9]. Co-design workshops were found to have benefited from a funneling of methodologies and methods, whether qualitative or quantitative, that reflect digital (ie, technical and usability) and health care (ie, medical and psychoanalytical) around end-user involvement. Those studies that found this to be successful noted that it is only possible where diverse stakeholder involvement around the end users is consistent throughout the iterative design process [9,38,89]. Values such as impartiality [116] and interdisciplinarity [127,177] were identified as key elements toward hybridizing approaches. These values recognized that the needs of stakeholders and end users may be highly disparate [16]. Therefore, if acceptability [127] is a ubiquitous term that satisfies digital goals of usability, health care goals of efficacy, and end-user goals of usefulness, then refining a co-design approach that is organically suited to the needs of the digital health ecosystem is paramount.

##### Subtheme: Fluid Digital Health Efficacy Evaluation

The call for a more organic, digital health co-design approach was backed by calls to integrate digital and health efficacy metrics into a singular framework for evaluation (39/171, 22.8%) [9,12,14,16,34,35,39,46,47,50,52,55,56,58, 61,64-66,69,71,78,80-82,92,96,99,104,106,107,118, 142,146,157,170,171,175,179-181]. Various studies found a siloed approach that demarcated digital and health stakeholders was ineffective toward curating interventions. Studies recognized that design workshops did not always account for technical usability, behavior changes or patient engagement approaches that are all part of the digital health evaluation ecosystem [9,92,106,126,149,157,170,180]. Determining user motivation and confidence did not account for health system constraints [180], and determining health safety did not account for user engagement. Essentially, studies found a need to bridge [14,39,106,118,170] digital concepts such as increased usability, with health care concepts, such as reduced risk, in a workshop environment that fluidly and holistically facilitates both needs around the end user.

##### Subtheme: Agile Digital Health Project Facilitation

Numerous studies (18/171, 10.5%) [3,11,35,50,53,62,64,66,67,82,98,117,121,123,152,155,171,182] discussed the challenge to funnel digital and health co-design approaches into an adaptation of Agile methodology. Agile has been used in DHIs as a rapid design framework in response to legacy methods such as RCTs, which span years [11]. Agile uses short-cycle iterations (ie, sprints) with small sample sizes (ie, teams) [35] producing mainly qualitative results. However, efforts to port Agile “out of the box” into digital health have been met with challenges. While Agile presents exciting methods to incorporate end users and produce rapid emotional responses, studies have found that this qualitative analysis struggles to incorporate the rigor of health care development [121]. For example, some studies found that in Agile design sprints, patient end users were unsure of what a health solution should look like [152]. This exemplified the difficulty of entirely democratizing Agile digital health workshops. This, coupled with the small sample size of Agile teams, raised questions on validity and generalizability [98]. Findings exemplified the need for having project leads, such as Scrum masters and product owners, who are familiar with health care contexts [182] and can effectively blend digital health design sprints that better satisfy the needs of all stakeholders. In this context, evidence-based and theory driven approaches can meet around user needs and preferences. As Schwartz et al [98] summarized in their study, “experimenting with agile adaptation in digital health will require a transparency of the development process, collecting shared difficulties and solutions toward a more standardized implementation suited to mHealth interventions.”

#### Theme: Industry Implementability

Industry implementability (13/171, 7.6%) [3,13,16,24,31,35,36,91,106,146,153,171,177] represented downstream awareness of the need to develop a more fluid digital health hybridized design framework around end users. This is being fueled by the emerging policy shift toward the digital empowerment of patient end users. This new environment is further integrating government policy and administration, eHealth records, and encrypted end-to-end access through various collaborations between government bodies, industries, and end users (ie, citizens). Understanding the challenge to co-design for medical devices versus consumer devices was highlighted by studies that found conventional UCD processes hitting the wall of medical device regulation [31]. Nonetheless, medical regulation is part of the digital health ecosystem and needs to be considered in the ideation “design features” [36]. The needs of distributed health services were found to differ considerably with end-user preferences [16]. This reality addressed the complexity of propelling nascent stage DHI co-design toward the market. From industry suppliers to government regulatory bodies to end users, potential blockers in postdevelopment stages loomed large due to misalignment with clinical workflow and health policy [91,106]. Cooperation between public and private sectors to deliver DHIs added layers of bureaucracy [3]. In some cases, conceptual prototypes were not implementable in a given health care information technology ecosystem [153] due to security or privacy policies. This challenge exposed a gap in the business model to identify critical success factors [171] that are often missing in digital health stakeholder collaborations around end users.

## Discussion

### Principal Findings

This review examined the advancements and challenges of co-designing DHIs with end users, highlighting key gaps and limitations that cut through a wide range of design methodologies. The burgeoning growth of the digital health industry is a major advancement in health care delivery, one that is seeing exciting developments and major investment. Participatory co-design approaches that promote collaboration and ideation around the end users, often in situ, are promoting positive behavior change by seeking to absorb a diverse stakeholder group into a more fluid digital health ethos. These iterative design approaches are often centered around UCD methodologies and other similar approaches (eg, human-, person-, and patient- centered approaches) that emphasizes a more holistic empathetic approach, positioning the end users as coequal in the design phase (Multimedia Appendix 1). Co-design is an exploratory process that experiences divergence and convergence. Through this disruptive process, the collective brain of a diverse stakeholder group is sought out in order to resolve conflicts and to amalgamate design needs. It is hoped that through these approaches, a common language and improved cultural context may form, leading to a better co-design outcome, one that provides both better efficacy and UX.

These promising developments are tempered by the sheer complexity of combining digital and health approaches into one funneled, organic design around the end user. The rapidity of co-design workshops, with their limited cohort size, has raised many questions about the validity and reliability of findings. While an RCT is the gold standard in health care validation, its timeframe often outlives the development of a digital solution [11]. In response to this, researchers are experimenting within the nascent stages of digital health design, porting a variety of research methodologies (ie, observational and experimental, whether qualitative or quantitative) and design methods (ie, Agile and behavior change, among others), mixing and matching concepts seeking out how best to validate DHIs with end users.

Exactly *how* this incorporation occurs is the flash point of conflict. Digital design perspectives that are heavily borrowed from the mainstream digital industry are rooted in UX approaches. Health design perspectives focus on long-term safety and efficacy (Multimedia Appendix 8). While digital approaches are suited to rapid innovation, pivoting changes in real-time, health approaches are rooted in rigor and safety, implementing change slowly with longitudinal data. The variance between qualitative and quantitative approaches, UX and behavior change, and efficacy and usability accentuates the downstream challenges that occur in ideation, testing, and implementation of DHIs. This culture clash calls for pragmatism. Hybridizing digital and health approaches such that the demarcation between them eventually dissolves is the apex of DHI co-design. To better achieve this, the digital health domain must become comfortable with the uncomfortable, allowing the tensions of hierarchical health approaches to collide with flat-management digital approaches. Finding common ground between complaints of digital UX approaches that oversimplify health concerns and health approaches that are bureaucratic and time intensive will be paramount in creating an equilibrium that blends competing agendas.

### Understanding the Advancements, Failures, and Challenges to Incorporate End Users in DHI Co-Design

#### Advancements

In response to the call for better contextual design [3] to curate better health outcomes [4] around end users, various digital, health, and behavioral approaches are being implemented in digital health. From a digital perspective, collaboration and ideation with end users is a response to traditional top-down waterfall approaches that backload testing and feedback late in the development process, when it is often too late to properly incorporate fundamental changes [15]. From a health care perspective, there is a push toward digital health citizens [183]; persons who take more control of their own health care, whether observing metrics, building their knowledge base, or proactively engaging health care professionals digitally. Our findings highlight that these 2 motivating factors are driving improvements in collaborative ideation and testing with end users in their natural environments, leading to improved behavior modification. These positive findings in participatory co-design are tunneling digital health toward a more organic design process with end users, one that is seeking to balance health care and purposeful design more fluidly. These positive developments are exemplifying theoretical concepts that advocate for more reciprocal, self-empowering approaches to co-design [184] with end users, as digital health began to emerge a decade ago. Nonetheless, this is still very much a nascent exploratory stage for digital health, one that through the process of discovery continues to uncover new challenges.

#### Failures and Challenges

The findings in this study indicate that participatory co-design with DHI end users has interconnected challenges that underscore the difficulty of amalgamating common interests, common language among stakeholders, and both systematic and innovative design approaches. These challenges culminate in methodological misalignments that produce variance in ideating and testing, resulting in divergent goals. These themes complicate the broader objective of defining a digital health framework around end-user involvement in co-design. Optimistically, some studies focused on the core theme of pragmatism, seeking to implement digital health solutions around end users that are fit for purpose, albeit more theoretically than experimentally. To resolve the key challenges identified in the Introduction section, namely the multiplicity of design approaches, resolving disparate understandings of the end user, and dissolving competing agendas [6,7,12,13], DHIs require frameworks that are truly representative of all aspects of digital health delivery, breaking through the common silo disconnects. From the messiness of brainstorming ideation to the effectiveness of user testing right through the product implementation and system-wide distribution, an evolution is needed, one that provides broader, richer data points to better understand end-user incorporation and to provide better validation of iterative design decisions. The need for more data points and better testing methods is supported by the fact that 112 of 171 (65.5%) studies in this review encountered failures or challenges in testing phases citing either a lack of end users or the limited diversity among them. This speaks to the lack of data impacting digital health design decisions and its overarching philosophies.

The complexity of bringing DHIs to market cannot be accomplished solely with small, often speculative user studies. Downstream implementation and policy concerns can render years of research fruitless. New frameworks must emerge that are holistic and consider a global outlook on digital health distribution. Doing so may require evolving the digital health ecosystem by dissolving the tribalism of qualitative versus quantitative approaches, instead mixing methods pragmatically to better account for the sheer breadth of digital health success factors. Theoretically, hybridizing the 3 pillars—digital (eg, experience and usability), health (eg, medical and psychoanalytical), and business (eg, critical success factors)—may be crucial for better collectivizing design insights, improving industry implementability, and reducing downstream failures in product market fit. Practically, there is a need for co-designing frameworks and platforms that provide rapidly accessible, contextual, and demographic data to better justify iterative design decisions around end users. The end user is a person, a patient, a user, and a consumer. Their incorporation spans many personas and multiple user journeys that beget numerous types of data points relative to the evaluation of a DHI, from preconception to postdelivery; a “whole of system” co-design approach around health end users will be paramount to the future.

**Figure 3:**
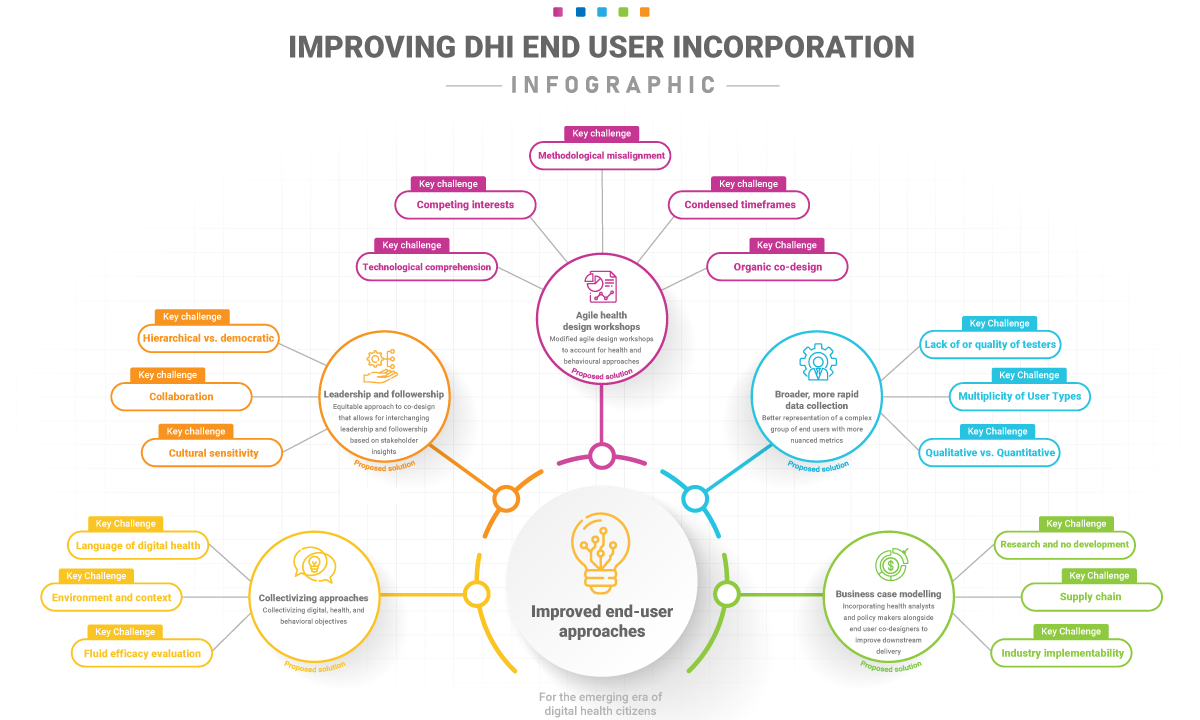
Mind map of key challenges and proposed solutions. DHI: digital health intervention.

On the basis our findings (Figure 3), we propose the core recommendations illustrated in subsequent sections.

#### Recommendation 1: Agile Health Design Workshops

Considering that Agile methodology is a project facilitation approach conceptualized outside of health care, unsurprisingly it has faced challenges when ported out of the box. To combine lightweight, rapid, qualitative Agile methods with more rigorous quantitative health methods, we recommend Agile workshops that triangulate emotional and observational feedback with statistical biosensor and analytical feedback that may soothe concerns about validity, generalizability, and attrition. This topic in itself could spawn a study on a mixed methods Agile framework design for digital health. Building on the calls for more Agile efficient approaches to digital health co-design suggested by Peiris et al [185] and Wilson et al [186], we propose the recommendations outlined in Textbox 2.

**Agile efficient approaches to digital health co-design.**

- Involving digital health agilists who have an overarching and empathetic view of the needs of digital, health, and end-user stakeholders. Coordinators of digital health projects (ie, Scrum masters and product owners) need to align the collective goals that are unique to digital health, ones that may not be solely reflected in traditional user experience design workshops.
- A preiteration sprint (ie, sprint 0) that involves creating shared digital health milestones and backlog grooming that combines the goals of health stakeholders (eg, clinical assessment, safety, and efficacy), digital stakeholders (eg, usability and technical validation), and end users (eg, user experience and acceptability). This preliminary approach may also account for discrepancies in technical ability and comprehension, as well as cultural sensitivities.
- From this, the selection of co-design activities that seek to combine these goals into an organic digital health workshop process may occur [171]. These activities should acquire metrics, whether qualitative or quantitative, that pragmatically fit the co-design objectives of a digital health team.
- Subsequent sprint retrospectives and sprint planning that continuously engages and reevaluates the collective goals of the digital health objectives and milestones, merging user experience validators with health and behavioral assessments.

Using the analogy of the toolbox, much like a carpenter must be pragmatic when selecting the given tools for the task at hand, digital health workshops must use a similar logic by hybridizing the methodologies, methods, and metrics that are fit for purpose [121]. Standardizing this process has proven difficult [98]. Shoehorning standard Agile practices into digital health continues to prove challenging, most notably in the struggle to validate studies with small participant groups in health care [104]. Therefore, there is a need to produce rapid quantifiable methods within the Agile design cycle, which points toward crowdsourcing ideation and testing data at scale. Charting a path that is reflective of collective co-designed digital health goals may reduce previously observed methodological misalignment, improving the flow of the co-design and building toward a more organic set of collective hoped outcomes. We do not suggest this as a magic elixir or standardized framework but as a flexible Agile health toolkit that seeks to amalgamate digital health approaches around the end user in the Agile workflow. This process must be tailored, balancing what participants say, do, imagine, test, and evolve, without being exhaustive [127]. As Schwartz et al [98] summarized in their study, experimenting with Agile adaptation in digital health will require a transparency of the development process, collecting shared difficulties and solutions toward a more standardized implementation suited to mHealth interventions. As a result, this may reduce downstream challenges of divergent objectives, hoped outcomes and failed implementation.

#### Recommendation 2: Account for the Multiplicity of End-User Types With Broader, More Rapid Data Collection

While traditional UCD workshops hone in on the end user as a single entity, DHIs are often more complex, with multiple types of end users. With 112 of 171 studies (65.5%) in this review citing challenges in either the number or variance of end users involved, recent digital health studies highlight the drawbacks of traditional UCD workshops, particularly in health care, where the “user (ie, patient) does not always have answers” [65] to clinical interventions [56]. The oversimplification of what a digital health end user is may deeply affect the integrity of the co-design. Considering that an mHealth app for heart medication may be used for tracking medication use; analytical data may serve multiple end users (eg, patient, caregiver, pharmacist, and clinician). Each type of end user has different goals, user experiences, and hoped outcomes. This reality was summarized well by Honary et al [139] where they keenly recognized that relatives of mHealth end users have different roles and responsibilities in the DHI. This growing pain in digital health is coming to the forefront as a study by Mak et al [48] advocated for mixed methods approaches that can better substantiate findings in diverse populations. To address this challenge and to account for the complexity of user types, broader pools of co-design data are needed; data that drill down on end-user demographics to create personas from rapid database-driven results. In this regard, a study by Tyo and Desroches [96] has experimented with the quantification of user personas to analyze clusters to better inform design requirements. These emerging studies speak to the need for quantifiable digital co-design platforms that can be deployed at scale.

#### Recommendation 3: Implement a Leadership and Followership Approach to Co-Design Workshops

A key flash point in the co-design process is the concept of democratic versus hierarchical approaches. The bottom-up (ie, shared and innovative) approach of design workshops is diametrically opposed to the top-down (ie, systematic and preplanned) approach of health care. In our findings, the high value of “equal coleadership” [6,14,127] was often championed as a guiding light in qualitative workshops. This has advantages and disadvantages, such that the democratic process brings many voices to the table, but this same process can eliminate evidence-based research from the solution [58]. Where criticism may exist of clinician-led studies being narrow minded, entirely democratic qualitative workshops can also be challenged for simplemindedness. For example, when a patient insists on a design preference [157] that conflicts with a safety issue backed up by years of quantifiable empirical data collected by clinicians. As Mirkovic et al [12] found, there is a need for equity over equality when it comes to workshop design with experts and end users. Undoubtedly, the value of a diverse array of stakeholders is of high importance; however, considering them all coequal through all stages of the design process does not serve to strengthen the design around the end user, rather it may weaken it if popular is preferred over practical, or entertainment over effectiveness. The plain democratization of the design process may lead to safety and efficacy concerns being voted off the island so to speak. Similarly, patient concerns with UX could be demoted since they are not central to behavior change or health policy goals.

The process of divergence and convergence in design workshops can instead benefit from a leadership and followership approach, such that design stakeholders lead the facilitation of UX objectives, health stakeholders lead the facilitation of safety and efficacy objectives, and patient stakeholders may lead discussion on their experiences and lifestyles [67,93,177]. In each example, the collaborative approach is central; however, there is identifiable leadership and followership such that a given expertise is provided enough ownership [101] to guide the workshop forward more effectively. This workshop approach may shift the perspective from *coequals* to *coleaders,* jointly seeking to learn from one another in a collaborative space. The goal of this approach would be to better balance person-centeredness, clinical acceptability, and UX [6] without eliminating expertise through a democratic design process.

#### Recommendation 4: Collectivizing Digital, Health, and Behavioral Objectives

A more pragmatic flexible framework that better understands the complexity of end-user types, one that seeks to blend the hoped outcomes of a disparate group of stakeholders, by necessity may benefit from collectivizing digital, health, and behavioral metrics and goals. Of the 171 studies, 70 (40.9%) studies listed various co-design challenges in collaboration and alignment. As the study by Ledel et al [7] found, both evidence-based (ie, health) and experience-based (ie, digital) approaches have merit for the end user, but friction exists when aligning these objectives. To reduce analysis in a vacuum and to align goals collectively, we suggest workshop co-design activities that blend objectives, such as safety (ie, health based) and usability (ie, digital based) can be blended into safe usability; behavior modification (ie, health based) and UX (ie, digital based) into user behavior; and efficacy (ie, health based) and uptake (ie, digital based) into effective uptake.

If the digital health end product is a fusion of digitally delivered health objectives, then the co-design process should suitably reflect this both philosophically and practically, aiming to reduce friction and combine goals and milestones. As Donovan et al [58] summarized in their study, “DHIs do not exist in isolation; they are integrated into existing clinical pathways.” Considering that stakeholder disengagement and disparity in hoped outcomes is a frequent challenge, the aim of designing interviews, observations, and testing, whether qualitative or quantitative, that blend approaches would be to increase the fluidity of the co-design process with end users. This fluidity may reduce the challenge of balancing the innovation equilibrium; letting designers innovate and health stakeholders evaluate, with patient experiences provide the link between the two. This environment can better accommodate clinical reality, incorporating biomedical knowledge, clinical workflows, and overarching health organizational requirements [35] around Agile practices that improve user collaboration, ideation, and rapid testing. This is very much an experimental process, but it begins with a change in thinking that can prevent implementation challenges downstream.

#### Recommendation 5: Include Business Case Modeling in the Co-Design Process for Downstream Implementability

Our final recommendation looks forward toward the downstream challenges of industry implementation and scalability. Rarely, in any of the studies observed, did we find co-design workshops that included health care policy or business stakeholders who addressed the clinical workflow challenges of delivering DHIs at scale to end users. Only 13 of 171 (7.6%) studies described the challenge of industry implementability, yet presumably every DHI project seeks to bring a viable product to market. In 2018, Hetrick et al [16] found that despite the success of their mHealth youth co-design, barriers exist toward the implementation of the app in clinical care settings that factor various service and governance factors. This identified the need to include health care analysts and policy makers in the co-design phase. Elements of public and private sector collaboration are interwoven into the fabric of health care service delivery that have their own set of needs that uniquely affect the constraints of a DHI. This factor needs to be incorporated into the co-design process to prevent blue-sky prototypes being discarded. A study by Vial et al [61] called for a transdisciplinary approach that includes business model definition coaching as a complement to the co-design team [61]. This accountability [15] recognizes the final leg of a DHI, the health care delivery platform. The inability to recognize this prevents scalability [12] and does not factor the ever-increasing integration of DHIs with government objectives to deliver ubiquitous services at scale.

### Preparing for the Era of Digital Health Citizens

These 5 recommendations are responses to the challenges identified in this review, with an eye toward the emerging era of digital health citizenry, in which techno-sociality and bio-sociality are undemarcating the boundaries of digital health [187]. This “health citizen” agenda envisions digital health as more of a public good that provides tools, opportunities, responsibilities, and rights [183]. This transformation is built upon the prosumerist nature of today’s technologies, such that any given DHI may be an end-user experience of patients, clinicians, caregivers, therapists, and passive observers collectively, facilitated through algorithmically driven big data solutions (ie, artificial intelligence and machine learning) that are intuitive, customizable, and adaptable. The delivery of health care is increasingly blending government (eg, health records and policy), medical (eg, public and private sector solutions), and citizens (eg, end users) into one fluid infrastructure. This relationship is becoming increasingly reciprocal, more productive than facilitative in nature [187]. The potential for crowdsourcing data in tomorrow’s era of digital health seems boundless.

Needless to say, this vision needs to be brought to the forefront of the messier, creative stages of co-design workshops. The reliance on small qualitative datasets does not leverage the vastness of crowdsourcing end-user feedback that is equally capable of collecting quantifiable, demographically contextual statistics, while garnering emotional qualitative data in the form of asynchronous digital co-design platforms. If there is one word to define the evolution of digital health moving forward, it may be pragmatism. Leveraging the right tools to incorporate end users in the design of DHIs should mean removing any boundaries to the type of data collected. This means removing any allegiances to particular schools of thought on qualitative or quantitative, observational, or statistical, instead focusing on a pragmatic “patient activation” [187], approach to garner positive involvement from end users at scale. There is no need for competing interests methodologically; a combination of metrics that increases the understanding of digital health users only serves to strengthen the confidence of design solutions, reducing attrition rates and increasing validity and generalizability. The digital health citizen is envisioned not as a user, an expert, or a novice, but as an active participant in the health care ecosystem, one who constantly learns and adapts to their health care needs. It is fluid and proactive rather than static and receptive. Therefore, there is a clear need to move the incorporation of end users in DHI co-design from small participant workshops objective to a dynamic socio-digital platform of ideating, testing, and evaluating in real time, at scale. Looking forward, this approach involves a new outlook that leverages the vision of the digital health citizen methodology, with the pragmatism of Agile tools, and the rigors of health care metrics. Experimenting with processes and tools that expand the range of data collection may better identify end-user types, better triangulate preferences and challenges, and provide stronger integration with health policies and implementation objectives downstream. Refocusing co-design approaches within this lens serves to better use the emerging potential of big data, potentially increasing the user perspectives of all stakeholders across the digital health spectrum, without sacrificing the rapidity and innovation of the design environment.

### Strengths and Limitations

This systematic review drew from a large swath of studies, using various methodologies and techniques to incorporate end users in DHIs. The strength of this approach was identifying a large number of studies for analysis and highlighting key global issues that digital health stakeholders face, including points of failure and challenges. We used the PerSPEcTiF framework due to its suitability to qualitative studies in health care. However, many of these studies were limited in the number of participants involved, bringing the validity and reliability of findings from these studies into question. The largely qualitative approach to studies selected, with vast variance in data collection approaches, did not allow for sensitivity analysis on the basis of measurement criteria (ie, setting, duration, and demographics).

A limitation also exists in the generalizability of findings toward specific scenarios (ie, mental health and patient aids) in which domain-specific usages weigh heavily on the context of end-user incorporation. We recommend further research to investigate these themes as subsets within specific digital health communities (ie, mental health and serious gaming) In addition, in many cases, studies did not provide perspectives on risk of harm when using approaches to incorporate end users. This issue requires critical analysis itself. With digital health being a hybrid domain, there is no standardized approach to quality assessment, leaving the potential for risk of bias. These factors complicate data extraction and interpretation. This may be improved by using a health-centric qualitative framework in such studies, such as Confidence in the Evidence from Reviews of Qualitative Research [188].

### Conclusions

The challenge to incorporate end users in the co-design of DHIs is complicated by a variety of methodological influences on problem definition, testing approaches, and hoped outcomes. The porting of legacy Agile, behavioral, and analytical methods has often resulted in conflicts that weaken conclusions that can be drawn from the co-design. In this study, we sought to draw a line through the common pain points and gaps in the literature that are omnipresent regardless of the design approach. The subsequent recommendations stemming from this systematic review serve as guidelines that may help soothe the path toward the big data environment that is emerging through a socio-technical digital health citizen approach into the mid-21st century.

## Appendix

Multimedia Appendix 1 A Venn diagram of the interdisciplinary intersection of digital health interventions.

Multimedia Appendix 2 End users in design of digital health interventions: search string queries.

Multimedia Appendix 3 End users in design of digital health interventions: screening template (2015 to 2021).

Multimedia Appendix 4 End users in design of digital health interventions: screening template (2022 to May 31, 2024).

Multimedia Appendix 5 End users in design of digital health interventions: data extraction template for categorization of themes and codes.

Multimedia Appendix 6 End users in design of digital health interventions: raw data content (ie, quotes and reflective analysis) extracted from eligible studies in the review.

Multimedia Appendix 7 Characteristics of included end-user studies.

Multimedia Appendix 8 Comparison of evaluation in digital and health-based perspectives.

Multimedia Appendix 9 PRISMA (Preferred Reporting Items for Systematic Reviews and Meta-Analyses) checklist.

## Abbreviations

DHI: digital health intervention
mHealth: mobile health
PRISMA: Preferred Reporting Items for Systematic Reviews and Meta-Analyses
PRISMA-P: Preferred Reporting Items for Systematic Reviews and Meta-Analyses Protocols
RCT: randomized controlled trial
UCD: user-centered design
UX: user experience

## Data Availability

All data generated or analyzed during this study are included in this published manuscript and its supplementary information files.
