# Supplementary material for: Examining Challenges to Co-Design Digital Health Interventions With End Users: Systematic Review
Source: J Med Internet Res. 2025 Mar 14;27:e50178. doi: 10.2196/50178 (PMC11953610; doi:10.2196/50178)
Supplement: Multimedia Appendix 1 [file jmir_v27i1e50178_app1.pdf]

INTERDISCIPLINARY DOMAIN OF DIGITAL HEALTH

Approaches and perspectives in digital health intervention design

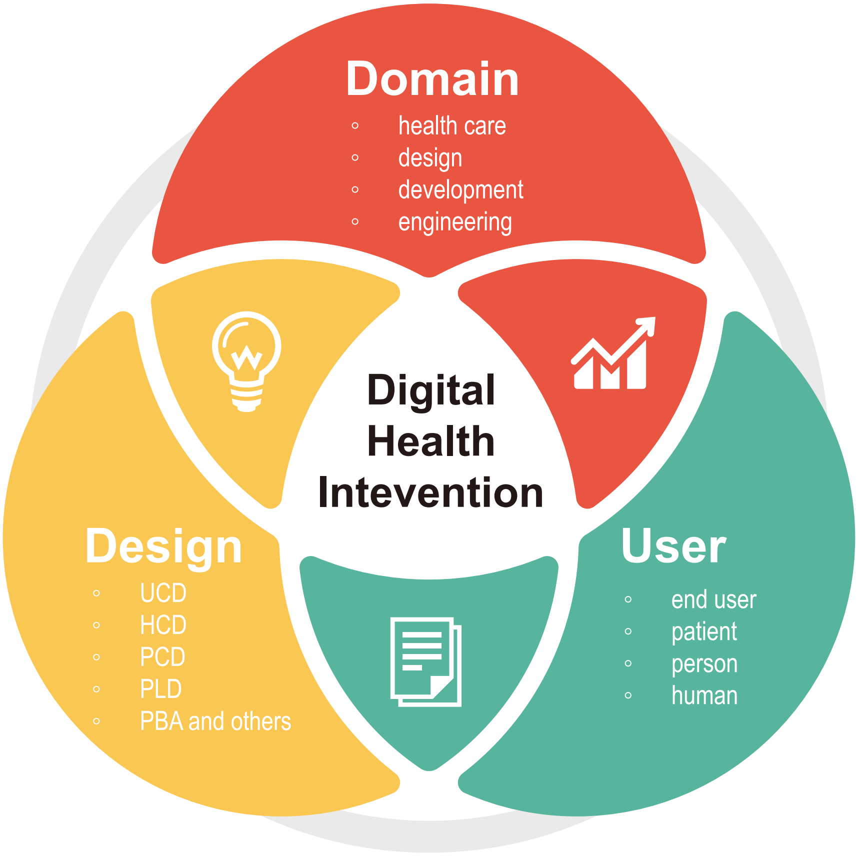

DESIGN

Various design methodologies such as user-centred, human-centred, patient-centred, patient-led, person-based and other hybrid approaches are employed that encompass different research philosophies, methods and metrics all aimed at incorporating end users.

DOMAIN

Various perspectives from health care to digital design, app development and engineering play a role in shaping a digital health intervention. This collaboration brings great promise to digital health but also creates domain silos and divergent goals.

USER

The user of a digital health intervention may be considered an end user, a patient, a person or human. This framing of the user of the intervention is largely premised on the domain and design employed.
