# Supplementary material for: Examining Challenges to Co-Design Digital Health Interventions With End Users: Systematic Review
Source: J Med Internet Res. 2025 Mar 14;27:e50178. doi: 10.2196/50178 (PMC11953610; doi:10.2196/50178)
Supplement: Multimedia Appendix 6 [file jmir_v27i1e50178_app6.pdf]

Examining Challenges to Incorporate End Users in the Design of DHIs - Full Text Review Notes

PREPARED FOR
Digital Health Circle

PREPARED BY
Anthony Duffy
PhD Student
Digital Health Circle Lab, School of Interactive Arts & Technology,
Simon Fraser University

January 1 2015 - May 31 2024

Plus Points **2**
Participatory Co-Design 2
Collaboration 2
Ideation 8
Improved Mode of Delivery 12
Behaviour Modification 13
Environment 14
In-Situ 14
Pain Points **16**
Participatory Co-Design 16
Collaboration 16
Culture Clash (Digital/Health/End User) 19
The Language of Digital Health Co-Design 19
Competing Interests 20
Hierarchical vs Democratic Design 26
Methodological Misalignment 31
Cultural Sensitivity 33
Cost and Scale 36
Environment and Context 39
Untested In-Situ 39
Multiplicity of User Types 43
Testing 45
Lack of Subjects 45
Quality of Subjects 54
Condensed Time Frame 59
Comprehension 63
Technology 63
Gaps in the Literature **65**
Pragmatic Hybridised Framework 65
Value of Mixed-Method Approaches 65
Organic Co-Design 70
Fluid Digital+Health Efficacy 74
Agile Digital Health 80
Industry Implementability 98
Supply Chain 98
Medical Device vs Consumer App 100

Colour Codes:

Yellow = Key Stat

Green = Key Positive

Red = Key Challenge

Plus Points

Participatory Co-Design

Collaboration

Keywords: complimentary, co-creation, involvement, ideation, prioritisation, perspective, mode of delivery, behaviour modification, improved patient-professional relationship, digestible (common understanding), self-directed, collective brain (truly evidence-based), vision, causes and consequences, resolving ambiguities, bottom up vs top down

Our team has **unique and complementary backgrounds** encompassing: HIT, human computer interaction, mHealth, UCD, UX evaluation, system design and architecture, human subjects protection, and clinical, and translational research.

Abujarad F, Alfano S, Bright TJ, et al. Building an Informed Consent Tool Starting with the Patient: The Patient-Centered Virtual Multimedia Interactive Informed Consent (VIC). *AMIA Annu Symp Proc*. 2017;2017:374-383. https://www.ncbi.nlm.nih.gov/pubmed/29854101

Typically, user-centered design of patient-facing dashboards involves an initial mock-up of some kind. We found that the participants, **working together in groups, were able to generate a set of dashboards and collaboratively decide on a prototype design** without such preliminary prompting. Our study reinforces that including patients and informal caregivers in the creation of an initial set of dashboards may **increase the level of user involvement in the design process overall.**

Working in a team with others who have similar backgrounds may generate more dynamic and thoughtful conversations, versus working one-on-one with a professional designer who does not have HF or a CIED.

Ahmed R, Toscos T, Rohani Ghahari R, et al. Visualization of Cardiac Implantable Electronic Device Data for Older Adults Using Participatory Design. *Appl Clin Inform*. 2019;10(4):707-718. doi:10.1055/s-0039-1695794, pmid:31533172

The HCD technique of cocreation supported stakeholders in a constructive, creative, and inclusive process that encouraged contributions and ideas from different perspectives. Insights from the exploratory research were critical to ensure that stakeholders truly understood the end users. These insights also informed the overarching principles for the design.

Andersson SR, Hassanen S, Momanyi AM, et al. Using Human-Centered Design to Adapt Supply Chains and Digital Solutions for Community Health Volunteers in Nomadic Communities of Northern Kenya. *Glob Health Sci Pract*. 2021;9(Suppl 1):S151-S167. doi:10.9745/GHSP-D-20-00378, pmid:33727327

In particular, parents wished for easily **digestible**, non-medical explanations as to what to expect during the treatment process, accompanied by advice for parents on how to prepare their child for their hospital stay in an age-appropriate manner.

Aufegger L, Bùi KH, Bicknell C, Darzi A. Designing a paediatric hospital information tool with children, parents, and healthcare staff: a UX study. *BMC Pediatr*. 2020;20(1):469. doi:10.1186/s12887-020-02361-w, pmid:33032549

As endorsed by children, adolescents, parents, and health care providers in phase 1, participants at the workshop generated pain management advice for the app that could be **self-directed** by the child or adolescent with minimal training, previous experience, or involvement or direction of an adult, such as a parent or health care provider.

Birnie KA, Campbell F, Nguyen C, et al. iCanCope PostOp: User-Centered Design of a Smartphone-Based App for Self-Management of Postoperative Pain in Children and Adolescents. *JMIR Form Res*. 2019;3(2):e12028. doi:10.2196/12028, pmid:31008704

This design and development study also has a number of strengths. First, the study employed key stakeholder involvement from the very beginning, including cancer survivors, health care providers working with survivors in various hospital units, eHealth experts including a designer and IT developers, the hospital Privacy and Security Protection Committee, and the Innovation unit. Having user involvement from the start and combining input obtained from the patient user with that obtained from health care providers along with evidence-based concepts and content likely increases the potential for the intervention to be effective.

Børøsund E, Mirkovic J, Clark MM, et al. A Stress Management App Intervention for Cancer Survivors: Design, Development, and Usability Testing. *JMIR Form Res*. 2018;2(2):e19. doi:10.2196/formative.9954, pmid:30684438

While it is tempting to wait to seek users or patient advice until a finished product is available, the insight and critique early in product development helped in ways that

could not have been envisioned at the beginning of the process. Despite their lack of software development experience, the patient advisers provided valuable comments and insight, which were vital to the development of MyIDEA

Boyd AD, Moores K, Shah V, et al. My Interventional Drug-Eluting Stent Educational App (MyIDEA): Patient-Centered Design Methodology. *JMIR Mhealth Uhealth*. 2015;3(3):e74. doi:10.2196/mhealth.4021, pmid:26139587

We involved patients and clinicians early in the P2P project in several ways, including informant interviews and feedback on analyses, requirements, and early design concepts. This helped learn lessons early, such as clinicians insisting that because of interindividual variability in physical activity, activity goals should be highly individualized, whereas other goals (eg, medication adherence) could be identical for all users. Early learning allowed earlier decisions about scope, facilitated evidence-based design choices, and prevented having to make costly future design revisions. In terms of the scope, early stakeholder involvement helped eliminate especially difficult or risky design concepts, for example, including medication titration advice.

Cornet VP, Toscos T, Bolchini D, et al. Untold Stories in User-Centered Design of Mobile Health: Practical Challenges and Strategies Learned From the Design and Evaluation of an App for Older Adults With Heart Failure. *JMIR Mhealth Uhealth*. 2020;8(7):e17703. doi:10.2196/17703, pmid:32706745

In addition to theory , evidence, and engaging design principles, mHealth interventions should also have social validity with regards to acceptability amongst its stakeholders [30]. Consequently, there is a growing trend towards adopting a user-centered design (UCD) approach [31-33]. This is especially pertinent in the case of apps where approximately **26% of all apps downloaded are discarded after first use** [34].

Curtis KE, Lahiri S, Brown KE. Targeting Parents for Childhood Weight Management: Development of a Theory-Driven and User-Centered Healthy Eating App. *JMIR Mhealth Uhealth*. 2015;3(2):e69. doi:10.2196/mhealth.3857, pmid:26088692

We found that working with members of the target population at all stages of the project was a useful strategy; stakeholder engagement aids the development of research interventions that are both adaptive to the needs of the patient and the preferences of the provider [37].

Davies A, Mueller J, Hennings J, Caress A-L, Jay C. Recommendations for Developing Support Tools With People Suffering From Chronic Obstructive Pulmonary Disease: Co-Design and Pilot Testing of a Mobile Health Prototype. *JMIR Hum Factors*. 2020;7(2):e16289. doi:10.2196/16289, pmid:32410730

The UCD based methodology of three cycles used in this study, which included patients and doctors, helped to understand how they perceive their role in the process of care, especially with respect to patients. This understanding allowed us to create a system that can support them in the management of the disease but also to

encourage the process of learning the **causes and consequences** of their decisions related to diabetes.

Fico G, Martinez-Millana A, Leuteritz J-P, et al. User Centered Design to Improve Information Exchange in Diabetes Care Through eHealth : Results from a Small Scale Exploratory Study. *J Med Syst*. 2019;44(1):2. doi:10.1007/s10916-019-1472-5, pmid:31741069

Applying a broad range of theory and best practice from the fields of health behavior, communication and user design is fundamental to the development of evidence-informed digital health.36,37 **This is especially critical for informed decision making in clinical trials which involves managing the emotional elements of uncertainty.**38

As Hamel and colleagues suggest, and our work confirms, digital health tools benefit from user centered design with a multi-disciplinary team bringing "critical theoretical and evidence based knowledge"14

Fleisher L, Bass SB, Shwarz M, et al. Using theory and user-centered design in digital health: The development of the mychoice communication tool to prepare patients and improve informed decision making regarding clinical trial participation. *Psychooncology*. 2020;29(1):114-122. doi:10.1002/pon.5254, pmid:31654442

A key strength of this study was the emphasis on user-centered design, following the guidance for design of complex interventions and principles of the person-based approach to intervention development (25, 26). The iterative methodology enabled participants to guide the development and provide their inputs at each stage. Sensitivity of the researchers and design team to local and cultural context, language, participant media preferences, and digital access helped focus on user needs and formed important considerations for the prototype design.

Gonsalves PP, Hodgson ES, Kumar A, et al. Design and Development of the "POD Adventures" Smartphone Game: A Blended Problem-Solving Intervention for Adolescent Mental Health in India. *Front Public Health*. 2019;7:238. doi:10.3389/fpubh.2019.00238, pmid:31508404

The time it took for each individual to analyze and provide feedback was on average 1 h. Within this hour, the individual was experiencing and commenting on context, was being formally interviewed, was filling out questionnaires, and was providing opinions on interface concepts. Therefore in one session the use case analysis provides multiple streams of data, whereas in previous literature, this kind of feedback would need to be gathered across multiple activities, such as surveys, interviews, and ethnographic observations.

Harte R, Quinlan LR, Glynn L, et al. Human-Centered Design Study: Enhancing the Usability of a Mobile Phone App in an Integrated Falls Risk Detection System for Use by Older Adult Users. *JMIR Mhealth Uhealth*. 2017;5(5):e71. doi:10.2196/mhealth.7046, pmid:28559227

Throughout the design process, both breastfeeding mothers and lactation consultants were engaged in the design of the Mother's Milk Connection application. This iterative

process ensured the end product would meet the needs of breastfeeding mothers. Engaging key providers, such as lactation consultants, allowed input on use of the Mother's Milk Connection ap- plication in the healthcare setting that captures essential data to guide individualized breastfeeding support.

Jefferson UT, Zachary I, Majee W. Employing a User-Centered Design to Engage Mothers in the Development of a mHealth Breastfeeding Application. *Comput Inform Nurs*. 2019;37(10):522-531. doi:10.1097/CIN.0000000000000549, pmid:31414995

Combining principles from these different design approaches enabled us to create a positive and open environment that engaged and supported the participants to explore and propose new overall ideas, specific features, and contexts for the implementation of tools that expand users' personal strengths and resources in a valuable and meaningful manner. For instance, the workshop started with an activity where the participants interviewed each other about their strengths, allowing them to both gain a better understanding of the concept of strengths by recognizing strengths in themselves and in others and to reflect and share positive personal experiences that they experienced while using them.

Mirkovic J, Jessen S, Kristjansdottir OB, Krogseth T, Koricho AT, Ruland CM. Developing Technology to Mobilize Personal Strengths in People with Chronic Illness: Positive Codesign Approach. *JMIR Form Res*. 2018;2(1):e10774. doi:10.2196/10774, pmid:30684404

We have also demonstrated that this approach promotes more holistic integrated pain care models [17,30] and enables consistency of messaging between consumers and their clinicians (eg, sharing of short targeted audiovisual content during clinical consultations about other people's pain experiences, and how they have implemented positive evidence-based behaviors to improve their pain care).

Slater H, Stinson JN, Jordan JE, et al. Evaluation of Digital Technologies Tailored to Support Young People's Self-Management of Musculoskeletal Pain: Mixed Methods Study. *J Med Internet Res*. 2020;22(6):e18315. doi:10.2196/18315, pmid:32442143

Ideation

Keywords: ideation, perspective, empathy, changing communication pattern, short cycles, rapid learning, multiple streams of data, divergence and convergence, MVP, rapid prototyping

The Scrum team met with researchers throughout the sprint cycles to gather user feedback and plan the tasks to be completed by the end of the next phase. A summary of the activities completed in each Scrum phase is described below.

Liu S, La H, Willms A, Rhodes RE. A "No-Code" App Design Platform for Mobile Health Research: Development and Usability Study. JMIR Formative Research. 2022;6(8):e38737. Accessed April 15, 2024. https://formative.jmir.org/2022/8/e38737

In general, this project also showed how conducting PD is necessary but still challenging. It has been difficult to plan several of the studies in advance. The use of the opportunistic approach allowed us to use the available local health care professionals throughout the design, development, and validation of RPs presented in this paper.

First, a specific set of methods from the user-centered design and PD methodologies was used. Second, the studies were conducted with a selected list of institutions and their experts as participants.

Khowaja K, Waheeda Syed W, Singh M, et al. A Participatory Design Approach to Develop Visualization of Wearable Actigraphy Data for Health Care Professionals: Case Study in Qatar. *JMIR Hum Factors*. 2022;9(2). https://www.scopus.com/inward/record.uri?eid=2-s2.0-85128862270&doi=10.2196%2f25880&partnerID=40&md5=b922d680f944ac3a25c677c64a1f97b3

Although all participants were involved in the prior focus groups that informed the design session materials, the hands-on PD process also **generated new ideas**, beyond those that emerged in the focus groups, suggesting that the method of creating a dashboard may have opened up new considerations.

Using patient preferences for information captured in prior focus group sessions, PD effectively established **prioritization** and visual presentation needs of older adults with HF as part of a larger iterative user-centered design process.

Ahmed R, Toscos T, Rohani Ghahari R, et al. Visualization of Cardiac Implantable Electronic Device Data for Older Adults Using Participatory Design. *Appl Clin Inform*. 2019;10(4):707-718. doi:10.1055/s-0039-1695794, pmid:31533172

The ideation and initial planning was the most mentioned work task, and participants commented that changing the physical location gives different **perspective**.

Ahtinen A, Andrejeff E, Vuolle M, Väänänen K. Walk as You Work: User Study and Design Implications for Mobile Walking Meetings. In: *Proceedings of the 9th Nordic Conference on Human-Computer Interaction*. NordiCHI '16. Association for Computing Machinery; 2016:1-10. Accessed February 8, 2022. https://doi.org/10.1145/2971485.2971510

New mobile devices provide **powerful tools for children in health care, with the potential for paying attention to their needs**. This can **change the communication pattern** between children and health care professionals, as well as strengthen children's empowerment [11,29].

An important challenge in research with children is to find appropriate ways for engaging and creating opportunities for children to have genuine influence on the research process [51]. Children in our study have added valuable views and quality of ideas regarding content, aesthetics, and usability that have genuinely influenced the design of

Sisom 2. Involving children throughout the low- and high-fidelity evaluation was considered as essential for the outcomes.

Arvidsson S, Gilljam B-M, Nygren J, Ruland CM, Nordby-Bøe T, Svedberg P. Redesign and Validation of Sisom, an Interactive Assessment and Communication Tool for Children With Cancer. JMIR Mhealth Uhealth. 2016;4(2):e76. doi:10.2196/mhealth.5715, pmid:27343004

Working in **short cycles** gives the ability to attain better results, value **rapid learning**, and fine-tune the existing work in a short period. Dow et al [33] stated that contributors working in cycles on a new task are as efficient as contributors working sequentially on a task they master.

Given that the work evolved through events, the games were developed using an agile methodology: prototypes were designed and a **minimal viable product** was developed and further improved by prioritizing the functions to be added.

One key learning outcome after hosting many **health game jams** is that such events are well adapted to raise awareness, foster collaboration, recruit long-term contributors, and create a multitude of prototypes to test different ideas. Because they are ephemeral, these events, however, do not allow the creation of high-quality games. Prototypes realized during game jams can, however, be redeveloped by a multidisciplinary team of professionals and tested during dedicated cocreation events.

Balli F. Developing Digital Games to Address Airway Clearance Therapy in Children With Cystic Fibrosis: Participatory Design Process. *JMIR Serious Games*. 2018;6(4):e18. doi:10.2196/games.8964, pmid:30463835

The PERSONAs definition supported the differentiation of user groups that finally resulted in tailored PHR registries. Placing the patient at the centre of the development process through UCD was the key.

Fico G, Martinez-Millana A, Leuteritz J-P, et al. User Centered Design to Improve Information Exchange in Diabetes Care Through eHealth : Results from a Small Scale Exploratory Study. *J Med Syst*. 2019;44(1):2. doi:10.1007/s10916-019-1472-5, pmid:31741069

The process of using an iterative approach was the main advantage in the intervention creation. This meant that development was continuously modified so that each component was not finalized until participant feedback was provided.

Humphries SM, Rondung E, Norlund F, et al. Designing a Web-Based Psychological Intervention for Patients With Myocardial Infarction With Nonobstructive Coronary Arteries: User-Centered Design Approach. *J Med Internet Res*. 2020;22(9):e19066. doi:10.2196/19066, pmid:32940615

The 2 first and the 2 latter stages make up a diamond of **diverging and converging** activities. Divergence refers to activities where designers (and co-designers) go broadly out to discover new opportunities, solutions, and ideas for their design. Conversely, the converging activities are focused on narrowing down, concretizing, and creating based on the former phase.

It is our position that those goals together make participatory approaches well suited. In addition, as our secondary goal is to explore opportunities for designing strengths-focused tools, it seems right indeed to include users, primarily as they should be considered to be experts of their own situation, and additionally as they can widen the design space [101] by, **for instance, contributing ideas that the researchers and designers would not think of.** Thus, users can also contribute productively in situations with solid evidence forming the basis of mHealth tools. As presented earlier, even how text is written and phrased benefited from user input. Thus, we would recommend considering all forms of creating mHealth tools, even small projects that, for instance, translate an existing tool, processes in which user participation can be of great value.

Jessen S, Mirkovic J, Nes LS. MyStrengths, a Strengths-Focused Mobile Health Tool: Participatory Design and Development. *JMIR Form Res*. 2020;4(7):e18049. doi:10.2196/18049, pmid:32706651

In this study , we organized a series of participatory design workshops with people living with chronic illnesses to jointly explore preferences, requirements, and ideas for gameful mHealth tools. The results of the study showed that engaging the participants with gamelike activities supported them to be collaborative, effective, and creative, especially by applying activities that set particular rules to their interaction (such as the rules of the game itself and the restriction of design elements). In addition, this approach provided the participants with a direction for their exploration of new ideas through, for example, the personas with their connected design challenges and the game elements cards. These findings are in line with previous studies that explore using gamelike participatory activities in design processes

Jessen S, Mirkovic J, Ruland CM. Creating Gameful Design in mHealth: A Participatory Co-Design Approach. *JMIR Mhealth Uhealth*. 2018;6(12):e11579. doi:10.2196/11579, pmid:30552080

Equal coleadership ensured that the main stakeholder perspectives of the project were always represented at the leadership table. In turn, these perspectives ensured that the design and development of Opal incorporated the necessary elements of person-centeredness, clinician acceptability, and informatics feasibility. A nonexhaustive list of these elements, as identified over the course of the project, is provided in Multimedia Appendix 2.

Kerkhof Y, Pelgrum-Keurhorst M, Mangiaracina F, et al. User-participatory development of FindMyApps; a tool to help people with mild dementia find supportive apps for self-management and meaningful activities. *Digit Health*. 2019;5:2055207618822942. doi:10.1177/2055207618822942, pmid:30944726

The first was a divergence of choices between caregivers and stakeholders (community and health network). It was decided to seek a solution acceptable to all parties through mediation. These tensions can be seen as democratic limitations resulting from the choice to involve stakeholders in the design process, but they can also be seen as creative

resources that are part of the co-design method because they bring to light issues that would have emerged sooner or later [48].

Latulippe K, Hamel C, Giroux D. Co-Design to Support the Development of Inclusive eHealth Tools for Caregivers of Functionally Dependent Older Persons: Social Justice Design. *J Med Internet Res*. 2020;22(11):e18399. doi:10.2196/18399, pmid:33164905

Concluding the workshop, we gained overwhelming positive feedback from the participants, stating that the overall positive focus helped to keep them interested and engaged during the whole day workshop. This was also reflected through the amount and variety of participants' feedback, as well as the themes and topics they discussed and the ideas that they proposed during the workshop. Thus, we can conclude that combining principles from participatory design, service design, and appreciative inquiry methodologies was an effective approach to enhance overall participant engagement, reflections, and creativity during the workshop with multiple stakeholders. This approach engaged them to think about new ideas and possibilities for technology that go beyond problem solving and addressing peoples' deficits.

This **ambiguity** of the term personal strengths reported by participants also influenced the idea-generating part of the workshop.

Mirkovic J, Jessen S, Kristjansdottir OB, Krogseth T, Koricho AT, Ruland CM. Developing Technology to Mobilize Personal Strengths in People with Chronic Illness: Positive Codesign Approach. *JMIR Form Res*. 2018;2(1):e10774. doi:10.2196/10774, pmid:30684404

While there are many instances where co-designing approaches have been successfully utilized for designing information architecture [28,29], our experience sheds light on two specific benefits of such approaches for mHealth apps. **First, co-design activities similar to ours can help user experience designers to become familiar with medical procedures and terminology without spending too much time on secondary research. For example, in our case, the user input gathered through co-design activities remarkably eliminated the need for designers to understand the content of the laboratory service manual.**

Saparamadu AADNS, Fernando P, Zeng P, et al. User-Centered Design Process of an mHealth App for Health Professionals: Case Study. *JMIR Mhealth Uhealth*. 2021;9(3):e18079. doi:10.2196/18079, pmid:33769297

Engaging all potential users also highlighted the need for extensive user-testing of the wording of multiple-user decision aids.

Corazza F, Snijders D, Arpone M, et al. Development and Usability of a Novel Interactive Tablet App (PediAppRREST) to Support the Management of Pediatric Cardiac Arrest: Pilot High-Fidelity Simulation-Based Study. *JMIR Mhealth Uhealth*. 2020;8(10):e19070. doi:10.2196/19070, pmid:32788142

Improved Mode of Delivery

Discussions in this study also echo findings regarding **preferred modes of health information delivery** in the SCI community. A 2010 review of physical activity information for SCI demonstrated a clear preference for face-to-face information delivery and for family, peers with SCI, and health professionals as information sources [47].

Allin S, Shepherd J, Tomasone J, et al. Participatory Design of an Online Self-Management Tool for Users With Spinal Cord Injury: Qualitative Study. *JMIR Rehabil Assist Technol*. 2018;5(1):e6. doi:10.2196/rehab.8158, pmid:29563075

The gold standard for testing complex interventions has traditionally been the randomised controlled trial (RCT). One of the methodological challenges in the field of digital health interventions is how to evaluate them when the field moves at such a swift pace. RCTs are time- and resource-intensive meaning a technology could be superseded or become obsolete before the end of a trial. In the field of mHealth, the iterative development process (with new releases and bug fixes) and personalisation of apps are not readily accommodated within the traditional RCT model whereby a rigid protocol is typically followed to assess a static intervention (Mohr et al., 2017; Murray et al., 2016; Pham et al., 2016). Thus traditional RCTs may often be an impractical evaluation approach for digital health interventions. Mohr et al. (2018), and Wilson et al. (2018) suggest we need to consider more agile and efficient approaches to mHealth development and evaluation lifecycles. Alternative frameworks and evaluation methods have been proposed that allow iterative changes to be made (West and Michie, 2016). Hybrid trial designs that combine evaluation of effectiveness and implementation potentially speed up the translation of research findings into real-world practice and increase the likelihood of successful uptake and adoption (Mohr et al., 2017). We will consider alternative, more agile methodological approaches when designing and planning future optimisation, evaluation and imple- mentation phases of MS Energise.

van Kessel K, Babbage DR, Kersten P, et al. Design considerations for a multiple sclerosis fatigue mobile app MS Energize: A pragmatic iterative approach using usability testing and resonance checks. *Internet Interv*. 2021;24:100371. doi:10.1016/j.invent.2021.100371, pmid:33614414

Behaviour Modification

Keywords: co-designing behaviour modification, multidisciplinary, personal (co-design) connections, therapeutic persuasiveness

one participant reported modifying behavior based on information shared during meetings. Such process-related benefits are common to PD, as its focus rests primarily on the development of participants and organizations; tools are seen as subsidiary

Allin S, Shepherd J, Tomasone J, et al. Participatory Design of an Online Self-Management Tool for Users With Spinal Cord Injury: Qualitative Study. *JMIR Rehabil Assist Technol*. 2018;5(1):e6. doi:10.2196/rehab.8158, pmid:29563075

During this phase, a **multidisciplinary** team should be involved from the beginning. Important inputs for our exergames design were that physiotherapists defined suitable exercises to help the mobility of seniors, developers studied the possibilities of the different tracking movement technologies such as Wii and Kinect, and game designers considered game elements, etc.

Brox E, Konstantinidis ST, Evertsen G. User-Centered Design of Serious Games for Older Adults Following 3 Years of Experience With Exergames for Seniors: A Study Design. *JMIR Serious Games*. 2017;5(1):e2. doi:10.2196/games.6254, pmid:28077348

The main strength of the current study is that end users were involved in developing FindMyApps. This is in contrast with the more traditional ‘waterfall’ method, a more top-down approach that does not include the end user in the development process, which is frequently associated with problems with usability, adoption and attrition.56

Kerkhof Y, Pelgrum-Keurhorst M, Mangiaracina F, et al. User-participatory development of FindMyApps; a tool to help people with mild dementia find supportive apps for self-management and meaningful activities. *Digit Health*. 2019;5:2055207618822942. doi:10.1177/2055207618822942, pmid:30944726

Processes and technology tools in health care tend to be designed in a silo and lack coordination with other aspects of care delivery [47]. The human-centered design method provides a structure that avoids the pitfalls common to traditional ICT development, such as lack of a deliberate process for innovation, all-in-one solutions that do not adequately meet users’needs, or solutions that are developed without user input [48,49]. For example, phase one of our interviews with case managers revealed that an ICT tool needed to focus primarily on efficiency to allow them to reach more clients. **However, phase two of our interviews with clients revealed that the personal connection is what makes the violence intervention program successful.**

Patel D, Sarlati S, Martin-Tuite P, et al. Designing an Information and Communications Technology Tool With and for Victims of Violence and Their Case Managers in San Francisco: Human-Centered Design Study. *JMIR Mhealth Uhealth*. 2020;8(8):e15866. doi:10.2196/15866, pmid:32831179

Furthermore, a recent study on design qualities that may predict user adherence to behavioral eHealth interventions in real-world use, found **therapeutic persuasiveness (defined as the incorporation of persuasive design/behavior change principles)** to be the most robust predictor of adherence (ie, duration of use and number of unique sessions),

suggesting the importance of persuasive design and behavior change techniques incorporation during the design and evaluation of digital behavioral interventions [45].

Slater H, Stinson JN, Jordan JE, et al. Evaluation of Digital Technologies Tailored to Support Young People's Self-Management of Musculoskeletal Pain: Mixed Methods Study. *J Med Internet Res*. 2020;22(6):e18315. doi:10.2196/18315, pmid:32442143

Environment

In-Situ

Keywords: lab challenges, lacks external validity,

For example, interviews were conducted in a place chosen by the interviewee. This ensured that each interviewee was in familiar surroundings and felt comfortable, which facilitat- ed more genuine, meaningful interactions and responses.

Andersson SR, Hassanen S, Momanyi AM, et al. Using Human-Centered Design to Adapt Supply Chains and Digital Solutions for Community Health Volunteers in Nomadic Communities of Northern Kenya. *Glob Health Sci Pract*. 2021;9(Suppl 1):S151-S167. doi:10.9745/GHSP-D-20-00378, pmid:33727327

P2P usability testing was conducted in a laboratory setting, **albeit in meeting rooms without built-in usability or simulation equipment** (eg, a control room, multicamera recording, eye tracking). Although this setting was adequate in most cases, it was at times inconvenient. The laboratory setting was more challenging because it required participants to test prototypes in a time and place dissimilar from the intended context of use.

Participants spent 30 min using a prototype technology meant to be used for weeks, Evaluating mHealth prototypes in a laboratory setting offers ideal conditions for detecting product software usability issues, such as navigation or layout issues. Such evaluation however **lacks external validity** in reproducing the context of the use of the mHealth product

Cornet VP, Toscos T, Bolchini D, et al. Untold Stories in User-Centered Design of Mobile Health: Practical Challenges and Strategies Learned From the Design and Evaluation of an App for Older Adults With Heart Failure. *JMIR Mhealth Uhealth*. 2020;8(7):e17703. doi:10.2196/17703, pmid:32706745

To the best of our knowledge, this paper is one of the first to describe the full process of design and development **of an app in hospital settings** using an SDLC model and to report its benefits and limitations.

Ehrler F, Lovis C, Blondon K. A Mobile Phone App for Bedside Nursing Care: Design and Development Using an Adapted Software Development Life Cycle Model. *JMIR Mhealth Uhealth*. 2019;7(4):e12551. doi:10.2196/12551, pmid:30973339

Firstly, rapid prototyping (i.e. the use of paper proto- types) proved to be of additional value to the needs assessment interviews. Patients (but also clinicians) often have difficulty conceptualizing what a PtDA is and how it should look and function, which might limit them in expressing their needs. With the use of rapid prototyp- ing, it was easier for users to express their wishes and needs and to give critical input. For this reason, we recommend using rapid prototyping in the development process of future PtDAs.

Nota I, Drossaert CHC, Melissant HC, et al. Development of a web-based patient decision aid for initiating disease modifying anti-rheumatic drugs using user-centred design methods. *BMC Med Inform Decis Mak*. 2017;17(1):51. doi:10.1186/s12911-017-0433-5, pmid:28441950

In addition, the sizable potential impact of this tool is currently limited by our knowledge of its effectiveness in real-world settings as well as its potential scalability through digital (web and mobile)

Ramsey AT, Bray M, Laker PA, et al. Participatory Design of a Personalized Genetic Risk Tool to Promote Behavioral Health. *Cancer Prev Res* . 2020;13(7):583-592. doi:10.1158/1940-6207.CAPR-20-0029, pmid:32209550

Pain Points

Participatory Co-Design

Keywords: novelty, (lack of) confidence, culture clash, iteration vs RCT, need for hybridisation, pragmatism, contextual barriers, uncomfortable, design method influence?, flash points, priorities, lost in the sauce,

Collaboration

An important finding was that user perspectives and usability priorities shifted with sustained use of the exciteBCI technology.

Alder G, Taylor D, Rashid U, et al. A Brain Computer Interface Neuromodulatory Device for Stroke Rehabilitation: Iterative User-Centered Design Approach. *JMIR Rehabil Assist Technol*. 2023;10(1). https://www.scopus.com/inward/record.uri?eid=2-s2.0-85180942123&doi=10.2196%2f49702&partnerID=40&md5=971587da81ea3e6deaa629234db24c7c

A significant strength of the present development process is the inclusion of children and young people at every stage. Owing to existing collaborations, we were able to involve young advisors (aged 12-19 years) in the early stages of our project, which had numerous benefits. However, the inclusion of slightly older participants in the PPI events may have also contributed to some of the issues we experienced in the workshops with younger children. We believe that the work with young advisors was very valuable but want to highlight that the involvement

Moltrecht B, Patalay P, Bear HA, Deighton J, Edbrooke-Childs J. A Transdiagnostic, Emotion Regulation App (Eda) for Children: Design, Development, and Lessons Learned. *JMIR Form Res*. 2022;6(1). https://www.scopus.com/inward/record.uri?eid=2-s2.0-85124142054&doi=10.2196%2f28300&partnerID=40&md5=b30e8fd39812b9b35473d717e36e6731

The role of clinician guidance and support is of considerable importance 73 and highlights the importance of clinician involve- ment and co-design.

Deady M, Collins D, Gayed A, Harvey SB, Bryant R. The development of a smartphone app to enhance post-traumatic stress disorder treatment in high-risk workers. *Digit Health*. 2023;9. https://www.scopus.com/inward/record.uri?eid=2-s2.0-85150077712&doi=10.1177%2f20552076231155680&partnerID=40&md5=807953d32125a0a6272cb256f2965726

we learned in our co-design workshops (flexibility, engagement, socialization, and a minimalistic design). Furthermore, our work extends previous work on planning successful co-design workshops for health-related apps [36,37], particularly for internet-based co-design workshops. Näkki and Antikainen [63] found that using web-based tools can make it easier and cheaper to include users as co-designers. People with disabilities benefit from being able to work in remote settings, which therefore promotes a more inclusive environment [64]. The Zoom-based co-design workshops, despite their limitations, provided flexibility for the survivor scientists in terms of geographical limitations and participants with disabilities being comfortable and able to work out of their own homes. In our work, to address RQ2, we found that successful co-design workshops should be engaging, inclusive, provide more time for participants to speak up, use smaller participant groups, and let participants think big (provide a universe where anything is possible).

Adler RF, Morales P, Sotelo J, Magasi S. Developing an mHealth App for Empowering Cancer Survivors with Disabilities: Co-design Study. *JMIR Form Res*. 2022;6(7). https://www.scopus.com/inward/record.uri?eid=2-s2.0-85136875442&doi=10.2196%2f37706&partnerID=40&md5=6ebe69d5f4cfa4cb6d021976bca38ebc

Participants did not choose to draw their own graphics and images. **It is possible that due to the novelty of the percent pacing data,** participants may have not felt confident enough about the subject matter to draw their own representations.

Ahmed R, Toscos T, Rohani Ghahari R, et al. Visualization of Cardiac Implantable Electronic Device Data for Older Adults Using Participatory Design. *Appl Clin Inform*. 2019;10(4):707-718. doi:10.1055/s-0039-1695794, pmid:31533172

Meaningful patient involvement in our co-design process led to an intuitive and functional prototype. Our website architecture was logical to patients and caregivers and allowed users to follow their own personal journey, where they could have different paths depending on their needs.

Donald M, Beanlands H, Straus SE, et al. A Web-Based Self-Management Support Prototype for Adults With Chronic Kidney Disease (My Kidneys My Health): Co-Design and Usability Testing. *JMIR Form Res*. 2021;5(2):e22220. doi:10.2196/22220, pmid:33560245

Design thinking was an effective method to develop an innovation in a **bottom-up approach**. However, it is possible that a different product would have been developed if another user-centered method had been used.

Ector GI, Westerweel PE, Hermens RP, et al. The Development of a Web-Based, Patient-Centered Intervention for Patients With Chronic Myeloid Leukemia (CMyLife): Design Thinking Development Approach. *J Med Internet Res*. 2020;22(5):e15895. doi:10.2196/15895, pmid:32412424

The technical team also benefited from the proximity with end users, as it allowed them to suggest and develop functionalities that corresponded better to the workflow or to usual practice.

Ehrler F, Lovis C, Blondon K. A Mobile Phone App for Bedside Nursing Care: Design and Development Using an Adapted Software Development Life Cycle Model. *JMIR Mhealth Uhealth*. 2019;7(4):e12551. doi:10.2196/12551, pmid:30973339

However, **mutual learning and shared understanding are core concepts** within participatory design, as this is the only way to ensure mutual respect between stakeholders, enabling everyone to take part in the shared decision-making process [56]. **Patients are not eHealth experts and do not necessarily have the language to articulate what they need from an eHealth intervention.** Consequently, using the same sample of participants and giving them enough knowledge about design and development processes may have made it easier for the participating patients to take an active part in development discussions. However, this study did also include new and naïve patients with chronic pain to add to previously collected qualitative data.

Ledel Solem IK, Varsi C, Eide H, et al. A User-Centered Approach to an Evidence-Based Electronic Health Pain Management Intervention for People With Chronic Pain: Design and Development of EPIO. *J Med Internet Res*. 2020;22(1):e15889. doi:10.2196/15889, pmid:31961331

Adaptations That Were Integrated Into the Tool Because of Disagreements With the Prototype

Several desirable interactive features were added to the final version of the tool, including the addition of mental health feedback scores. The participants were provided with an email regarding their depression and stress levels. This was deemed important as, in our previous qualitative analysis, it was identified that employees who did not have a formal diagnosis had little knowledge of whether their mental health symptoms would meet diagnostic criteria [9].

Stratton E, Choi I, Peters D, Calvo RA, Harvey SB, Glozier N. Co-Designing a Web-Based Decision Aid Tool for Employees Disclosure of Mental Health Conditions: A Participatory Study Design Using Employee and Organizational Preferences. *JMIR Form Res*. 2020;4(11):e23337. doi:10.2196/23337, pmid:33155982

Although strengths include the participation of a large number of people with a low educational level and the inclusion of clients from each sector, a **weakness is the low participation rate of clients from the nursing and care of the elderly sector**

van Leersum CM, Moser A, van Steenkiste B, et al. What matters to me - a web-based preference elicitation tool for clients in long-term care: a user-centred design. *BMC Med Inform Decis Mak*. 2020;20(1):57. doi:10.1186/s12911-020-1067-6, pmid:32183786

Culture Clash (Digital/Health/End User)

Keywords: tension, control vs iteration, disease management vs health and well being vs fun, balance agendas, know your audience, overwhelmingness, authority, hierarchical cultural structure, intimidation, disagreement, challenges, methodological and cultural misalignment, design bias, oversimplification, clinical reality, standardisation vs iteration, mismatched terminology, conflicting views, competing priorities, different reasons, different agendas, privacy (conceptually), blue sky vs empirical data, power roles, merging interests, indifference (healthcare professionals), paternalistic approach, asymmetrical relationship, divergence and convergence

The Language of Digital Health Co-Design

Particularly in a web-based co-design workshop, dreaming big could be even more difficult for participants with limited technical skills.

Adler RF, Morales P, Sotelo J, Magasi S. Developing an mHealth App for Empowering Cancer Survivors with Disabilities: Co-design Study. *JMIR Form Res*. 2022;6(7). https://www.scopus.com/inward/record.uri?eid=2-s2.0-85136875442&doi=10.2196%2f37706&partnerID=40&md5=6ebe69d5f4cfa4cb6d021976bca38ebc

Tailoring the phrasing and word choice of the application content to effectively describe the intent of the application to the end-user is a major priority. **The semantic mismatch we observed in patient/care partner understanding of the phrase “safety concern” is a great**

example of how there can be impactful differences in interpretation between clinical/research staff and patients and care partner, in this case around patient safety concepts. We learned that when a patient hears "safety" they think "physical harm" that has already occurred and tended to focus primarily on medication-related events and not consider other types of near misses, such as communication concerns.

Couture B, Lilley E, Chang F, et al. Applying User-Centered Design Methods to the Development of an mHealth Application for Use in the Hospital Setting by Patients and Care Partners. *Appl Clin Inform*. 2018;9(2):302-312. doi:10.1055/s-0038-1645888, pmid:29742756

In sprint 2, the development moved toward the design aspect of the CeHRes roadmap during which the first version of the digital health intervention is communicated with end users to collect feedback. **It is recommended to initially present a prototype that does not fully resemble the final product but does include the essential features and then build on successive prototypes [9].**

Rai HK, Schneider J, Orrell M. An Individual Cognitive Stimulation Therapy App for People With Dementia: Development and Usability Study of Thinkability. *JMIR Aging*. 2020;3(2):e17105. doi:10.2196/17105, pmid:33196451

In a conventional human-centered design, a summative evaluation would have been conducted to ensure that the usability of the PPT and PPA had been improved and that there were no residual issues that could impede use or generate adverse events. However, clinicians expressed a desire to see the tool installed quickly in the PED. With respect to the intent of use of the tool (to help prioritize patients, excluding patients in a life-threatening situation, without imposing this prioritization), the potential risks arising from usability issues would be misinterpretations of the information provided, with the worst consequence being a possible increase in waiting times for some patients and a rejection of the tool by clinicians.

Schiro J, Pelayo S, Martinot A, Dubos F, Beuscart-Zéphir M-C, Marcilly R. Applying a Human-Centered Design to Develop a Patient Prioritization Tool for a Pediatric Emergency Department: Detailed Case Study of First Iterations. *JMIR Human Factors*. 2020;7(3):e18427. Accessed February 8, 2022. http://humanfactors.jmir.org/2020/3/e18427/

(iv) It is characterized by quick iterations of de- signing, testing, and improving again, which in- volves rather frequent involvement of the target group and close cooperation between designers and software developers. **However, it may take a lot of time for the older adults to get acquainted with and committed to the topic and the project team, and to understand the way of work- ing.** The group synergy would benefit from the participatory design team consisting of the same members throughout the entire process, rather than involving new members for each iteration.

Also, when actually carrying out participatory design sessions with older adults, the following practical issues should be considered:

(i) Reserve time for participants to get to know each other and to chitchat about other topics than the project at hand. Be aware of the fact that participation in the sessions is a social activ- ity in itself;

Verhoeven F, Cremers A, Schoone M, Van Dijk J. Mobiles for mobility: Participatory design of a “Happy walker” that stimulates mobility among older people. *Gerontechnology*. 2016;15(1). https://journal.gerontechnology.org/currentIssueContent.aspx?aid=2248

Competing Interests

In addition, it seemed difficult for some co-designers (both caregivers, HSSPs, and community workers) to think in numerical terms, especially at the beginning of the process, during brainstorming activities to create functionalities targeted to meeting needs (CoD1 to CoD4). On several occasions, **the solutions or reflection focused on the health system and current services rather than on the use of digital technology to meet the caregiver’s needs.** The research team repeatedly refocused the co-designers on the objective of developing a digital tool targeted to meet the needs of caregivers

Latulippe K, Hamel C, Giroux D. Co-Design to Support the Development of Inclusive eHealth Tools for Caregivers of Functionally Dependent Older Persons: Social Justice Design. *J Med Internet Res*. 2020;22(11):e18399. doi:10.2196/18399, pmid:33164905

The research or multimedia teams were involved throughout the project, and it is possible that their personal views could have influenced the process of development.

Bevan Jones R, Thapar A, Rice F, et al. A Web-Based Psychoeducational Intervention for Adolescent Depression: Design and Development of MoodHwb. *JMIR Ment Health*. 2018;5(1):e13. doi:10.2196/mental.8894, pmid:29449202

Thirdly, **we recommend not only asking health professionals about their perception of patients’ needs, but also asking them about their own needs and thoughts on implementing a PtDA into their practice.** Their practical and expert knowledge on the decision-making process can be of great value for the integration of a PtDA into thepatientpathwayanddailyworkflowofhealthprofes- sionals, and consequently enhance the adoption and imple- mentation of the PtDA.

The adoption and implementation of PtDAs using a referral model (i.e. health professionals inviting eligible patients to use the PtDA) is often chal- lenged by **indifference** on the part of health professionals [43]. This indifference may stem from a lack of confidence in the content of the PtDAs and concerns about disruption of established workflows [43

This indicates that the iterative and extensive involvement of health professionals and the acknowledgement of their needs for the PtDA were important in **creating ownership.**

Nota I, Drossaert CHC, Melissant HC, et al. Development of a web-based patient decision aid for initiating disease modifying anti-rheumatic drugs using user-centred design methods. *BMC Med Inform Decis Mak*. 2017;17(1):51. doi:10.1186/s12911-017-0433-5, pmid:28441950

There is a need to balance the financial and time cost of such upgrades with continuing with the study as planned and budgeted. There can also be tension between the study team and the vendor when there is disagreement about the best features and how to modify them.

Schwartz LA, Psihogios AM, Henry-Moss D, et al. Iterative development of a tailored mHealth intervention for adolescent and young adult survivors of childhood cancer. *Clin Pract Pediatr Psychol*. 2019;7(1):31-43. http://doi.apa.org/getdoi.cfm?doi=10.1037/cpp0000272

It was clear that all stakeholders shared the common vision of improving quality and safety through patient feedback but there were **conflicting views** about how this could be achieved. This appeared to stem from the competing pressures and priorities experienced by those involved.

It also became apparent that incorporating a separate co-designed intervention into an established IT system created limitations in functionality, which sometimes prevented the system being a true representation of the key principles and components that stakeholders established as being essential. Consequently, there was a tension between the views of the patient-led steering group and those responsible for the technical specifications of the existing system.

Consideration therefore needed to be given to whether the **competing priorities** of 'sharing thoughts' and RPV could co-exist in one system. In order to successfully merge with an existing system (RPV), careful consideration needs to be given to the environment and context in which it currently exists. Therefore, future work should consider the socio-political context in which these systems exist and are developed within (Kincheloe & McClaren 2000). Although the IT experts were engaged with the design process, 'sharing thoughts' was one of a number of studies with which they were involved. We, therefore, suggest that researchers need to consider this during the initial planning stage to ensure that IT experts are able to play a more central role in the collaborative process.

Patients were keen to have a truly anonymous system, whereas **healthcare professionals felt that this compromised their ability to investigate concerns**. By focussing on the need to identify individuals and investigate individual issues raised, the staff view conflicted with the systems approach to the investigation of error (Reason 2000). The patients were more in favour of taking a general overview of common concerns with a view to addressing them as a group rather individually. This was also driven by a fear of

the consequences, recognised as a theme by Robert Francis QC in the poor management of patient complaints (Francis 2013), and further reported in a recent review of the NHS Complaints System (Clwyd & Hart 2013).

The design and development process in this study did reveal some disagreements between what was considered important by health care providers and other collaborating partners versus what was considered important by some of the patients.

Although health care providers emphasized the need for available, evidence-based, and trustworthy information given to patients, seeing eHealth technology as a positive option for providing patients with such knowledge, patients expressed some conflicting views on the topic. Patients generally agreed that information and content should be trustworthy, yet they kept emphasizing during workshops as well as usability testing that they did not want too much information, that they already knew a lot about pain and the theory behind pain management, and that they first and foremost wanted effective and quick exercises that could help in their daily lives.

On the basis of these findings, it was important to find a balance in the use of design elements, with the final EPIO program including some of these types of elements, such as **trophies for progress and continued use; and an avatar/buddy**, the bird EPIOS; and providing users with content summaries and brief motivational messages.

Ledel Solem IK, Varsi C, Eide H, et al. A User-Centered Approach to an Evidence-Based Electronic Health Pain Management Intervention for People With Chronic Pain: Design and Development of EPIO. *J Med Internet Res*. 2020;22(1):e15889. doi:10.2196/15889, pmid:31961331

Giles SJ, Reynolds C, Heyhoe J, Armitage G. Developing a patient-led electronic feedback system for quality and safety within Renal PatientView. *J Ren Care*. 2017;43(1):37-49. doi:10.1111/jorc.12186, pmid:27990782

Owing to their status, they may, however, **overshadow the experience and needs of patients** and push the focus on **managing the disease versus the promotion of health and well-being of a person overall in the long term**. Bringing too much medical content may reduce the fun and impact of a game.

Balli F. Developing Digital Games to Address Airway Clearance Therapy in Children With Cystic Fibrosis: Participatory Design Process. *JMIR Serious Games*. 2018;6(4):e18. doi:10.2196/games.8964, pmid:30463835

Participants have their own **agendas** when participating in a workshop, e.g. specialists spent more time explaining the situation in their clinics and their views of what patients need in general, than expected and often did not respond directly to the question asked.

Plan for participants to take time to explain their situation. This provides more **context** for their **perceptions and expectations** of the situation, allows the research team to better understand their needs, and may provide additional and unexpected relevant information.

Know your audience - What you see as important to the core purpose of the project may not be relevant for the participants.

Do not overwhelm participants with information, especially at the beginning when their priority is to get settled in and comfortable. **Test out your explanation on someone completely unrelated to the project,** e.g. a family member or friend, and ask that they point out the confusing or unnecessary details.

Engaging and creative activities were planned based off of research and online "toolkits" available from several difference organizations. Despites attempts to make instructions as straightforward and clear as possible, participants felt the need to clarify several times because the instructions were either too detailed and complicated or not understandable.

5. During the joint sessions, ensure that both patient and healthcare provider participants feel comfortable and safe to share their opinions, despite the difference in perceived **"authority level"**. We expected to need to reiterate that everyone's opinion is their own and should be respected. However, possibly due to the less **hierarchical cultural structure** in Norway, we did not need to reinforce this concept. Participants were respectful and listened without having to be directed.

7. Allow for the participants to drive the conversation and tell the research team what they need and ideas for the systems' design Some participants seemed unfamiliar and **uncomfortable** with suggesting creative solutions for a future system. Instead they wished for us to present prototypes and then form a discussion based off of existing ideas.

Bradway M, Morris RL, Giordanengo A, Årsand E. How mHealth can facilitate collaboration in diabetes care: qualitative analysis of co-design workshops. *BMC Health Serv Res*. 2020;20(1):1104. doi:10.1186/s12913-020-05955-3, pmid:33256732

Although eHealth CKD self-management support interventions are burgeoning, interventions rarely consider a behavioral theoretical framework to investigate individual behavior change [7,34].

Donald M, Beanlands H, Straus SE, et al. A Web-Based Self-Management Support Prototype for Adults With Chronic Kidney Disease (My Kidneys My Health): Co-Design and Usability Testing. *JMIR Form Res*. 2021;5(2):e22220. doi:10.2196/22220, pmid:33560245

The development of the app, using a codesign process, is a significant advance on many of the currently available apps (and other technology-based interventions) that have been designed to address mental ill health because of the incorporation of the youth perspective. **The inclusion of both clinicians and young people in a codesign process highlighted disparate needs, motivations, and intentions for the app, and by incorporating the views of both, the app has promise as a tool to assist both clinicians and young people in the management of depression and suicide-related behaviors**

Although our app conforms to clinical practice guideline recommendations with regard to routine monitoring of symptoms (depression) and medication side effects (suicidal ideation) and its prototype has been beta-tested [10], **there is a need to robustly test the app for efficacy and safety,** including testing that the innovative mood rating function is a reliable and valid measure of mood compared with validated measures such as the PHQ-9 and our 3-item suicide risk screener [10].

Hetrick SE, Robinson J, Burge E, et al. Youth Codesign of a Mobile Phone App to Facilitate Self-Monitoring and Management of Mood Symptoms in Young People With Major Depression, Suicidal Ideation, and Self-Harm. *JMIR Ment Health*. 2018;5(1):e9. doi:10.2196/mental.9041, pmid:29362208

Furthermore, actively engaging stakeholders in the iterative design process were challenging to achieve and require experienced facilitators. One of the participants left the project due to a **lack of interest.**

Naeemabadi M, Søndergaard JH, Klastrup A, et al. Development of an individualized asynchronous sensor-based telerehabilitation program for patients undergoing total knee replacement: Participatory design. *Health Informatics J*. 2020;26(4):2492-2511. doi:10.1177/1460458220909779, pmid:32175788

There were 2 main areas where misalignment appeared between the workers and organizational preferences and the prototype developed by experts. The first was the suggested use of an avatar by the developers. Avatars have previously been used in medical settings. The developers were initially interested in including an avatar into the intervention to assist those with lower literacy levels. Previous studies utilizing avatars to deliver medical information to patients with low literacy indicated that the avatar provided an additional authoritative source for their medical information and a majority preferred receiving the information via the avatar compared with reading the information themselves [29]. An avatar assisted veterans with postdeployment distress in help-seeking decisions. Those randomized to an avatar group exhibited significantly greater likelihood of recognizing their symptoms and seeking help for their mental health concerns compared with a control [30]. However, in this scenario, with such a strong negative response from the worker preference discussions, it was decided not to include the avatar to minimize potential harm as participants suggested that the use of a cartoon may be downplaying the seriousness of mental ill health.

Stratton E, Choi I, Peters D, Calvo RA, Harvey SB, Glozier N. Co-Designing a Web-Based Decision Aid Tool for Employees Disclosure of Mental Health Conditions: A Participatory Study Design Using Employee and Organizational Preferences. *JMIR Form Res*. 2020;4(11):e23337. doi:10.2196/23337, pmid:33155982

Research about clinical decision support in PD focuses largely on early detection and diagnosis of PD [41,42], but the participants in our study stressed the value of clinical decision support and self-care recommendations related to the management of already diagnosed PD. **A European Union–funded project has reported on the design of**

a clinical decision support system for PD that takes a holistic approach, which is in line with the functionalities suggested in this study [45-47].

Verhoeven F, Cremers A, Schoone M, Van Dijk J. Mobiles for mobility: Participatory design of a "Happy walker" that stimulates mobility among older people. *Gerontechnology*. 2016;15(1). https://journal.gerontechnology.org/currentIssueContent.aspx?aid=2248

In this study, we found a discrepancy in the perspectives of health care professionals and patients with regard to the consideration of gaming elements. Health care professionals were open to the inclusion of gaming elements, whereas patients were cautious in considering gaming elements. It is extremely important to take into consideration the needs of patients, as they are the eventual final users. Allowing patients' perspectives to take dominance over health care and academic perspective makes sense, given that it is increasingly recognized that patients bring unique insights and knowledge into the co-designing process [42]. **This discrepancy in viewpoints could potentially be mitigated if health care professionals and patients are both included in the same co-design workshop. Unfortunately, this was not permitted by our ethical board, as there are concerns that patient participants might not be as vocal due to the presence of health care professionals.**

Zhang M, Heng S, Song G, Fung DS, Smith HE. Co-designing a Mobile Gamified Attention Bias Modification Intervention for Substance Use Disorders: Participatory Research Study. *JMIR Mhealth Uhealth*. 2019;7(10):e15871. doi:10.2196/15871, pmid:31584003

Furthermore, most e-Health systems are used by different kind of users with separate needs and/or roles (e.g., nurses and clinicians or patients and caregivers) what leads to the imperative of considering their requirements separately.

Curtis KE, Lahiri S, Brown KE. Targeting Parents for Childhood Weight Management: Development of a Theory-Driven and User-Centered Healthy Eating App. *JMIR Mhealth Uhealth*. 2015;3(2):e69. doi:10.2196/mhealth.3857, pmid:26088692

Hierarchical vs Democratic Design

Paternalistic attitudes are no longer desirable, as they increase the asymmetry in the relationship and finally lead individuals to agree with the health professional's decisions [92,93].

Ospina-Pinillos L, Davenport TA, Navarro-Mancilla AA, et al. Involving End Users in Adapting a Spanish Version of a Web-Based Mental Health Clinic for Young People in Colombia: Exploratory Study Using Participatory Design Methodologies. *JMIR Ment Health*. 2020;7(2):e15914. doi:10.2196/15914, pmid:32027313

Despite the hierarchy that is observed in the medical culture [23], we observed more collaborative work and interdependency during design activities and discussions. However, it was observed that mostly doctors started leading the group during the technical discussions including information architecture, and nurses were eager to get the doctors involved. During wireframe testing and participatory design activities, the nurses were more inclined to pen down their ideas, and doctors were more collaborative despite leading the group. Disagreements between group members were dealt with through ongoing discussions; however, in certain instances we observed outlying ideas being rejected by doctors and experienced nurses without their merits being carefully examined.

Saparamadu AADNS, Fernando P, Zeng P, et al. User-Centered Design Process of an mHealth App for Health Professionals: Case Study. *JMIR Mhealth Uhealth*. 2021;9(3):e18079. doi:10.2196/18079, pmid:33769297

During the workshop, the varied backgrounds of participants contributed to producing innovative ideas that we, as researchers and designers, would not have considered alone without their insights. **However, while stakeholders' input about their challenges, preferences, needs, and values are crucial, creating successful new tools and systems also requires the specific knowledge and expertise of experts (eg, designers and researchers) to make sure the functional purpose, existing evidence, and the standards of good design are followed and addressed [42].**

Mirkovic J, Jessen S, Kristjansdottir OB, Krogseth T, Koricho AT, Ruland CM. Developing Technology to Mobilize Personal Strengths in People with Chronic Illness: Positive Codesign Approach. *JMIR Form Res*. 2018;2(1):e10774. doi:10.2196/10774, pmid:30684404

Mixing participants in heterogenous groups during group work activities could potentially have introduced power disbalance in the groups and affect participants' open participation and engagement during workshop. For example, **a health care provider is usually seen by patients as having a power role, and as a result, patients may not feel as comfortable reporting dissatisfaction or frustration** with their health care providers and whole health care systems in the presence of other health care providers. **However, if organized in the right manner, heterogenous groups can be highly effective and constructive by letting the participants exchange stories and build on different knowledge and needs stemming from their different backgrounds and experiences [71].** Therefore, to minimize the potential limitations of heterogenous group work in our study, different measures were applied: the rules of conduct that promote joined work and cooperation were clearly defined at the start of the workshop; the group work and activities was designed to promote participation, collaboration, and sharing ideas; and facilitators were trained to facilitate openness and active participation of all participants.

Mirkovic J, Jessen S, Kristjansdottir OB, Krogseth T, Koricho AT, Ruland CM. Developing Technology to Mobilize Personal Strengths in People with Chronic Illness: Positive Codesign Approach. *JMIR Form Res*. 2018;2(1):e10774. doi:10.2196/10774, pmid:30684404

Although full and equal coleadership was a fundamental element of our participatory stakeholder co-design approach, we found that this was often not clear to people outside of the immediate design and development team. External to the team, it was necessary to continuously insist that all 3 coleads were equal. **The tendency in the hierarchical health care system is to assume that a project must be led by a clinician, and it is commonly assumed that the patient participant is a token member of the team.** Overcoming this required **persistence**, it ensured that the team was truly coled and it broke new ground within the hospital by demonstrating the benefits of full and equal patient involvement. **Equal coleadership was vital to the success of the project.**

Kerkhof Y, Pelgrum-Keurhorst M, Mangiaracina F, et al. User-participatory development of FindMyApps; a tool to help people with mild dementia find supportive apps for self-management and meaningful activities. *Digit Health*. 2019;5:2055207618822942. doi:10.1177/2055207618822942, pmid:30944726

There are also limitations of the study that need to be mentioned. One limitation is that the development team had to make choices based on a limited amount of data. Establishing a useful group hierarchy and selecting suitable icons representing the main and subcategories could have been a separate study, instead of part of the current study. The development team struggled to choose the most suitable icons for people with dementia.

Kerkhof Y, Pelgrum-Keurhorst M, Mangiaracina F, et al. User-participatory development of FindMyApps; a tool to help people with mild dementia find supportive apps for self-management and meaningful activities. *Digit Health*. 2019;5:2055207618822942. doi:10.1177/2055207618822942, pmid:30944726

Furthermore, most e-Health systems are used by **different kind of users** with **separate needs and/or roles** (e.g., nurses and clinicians or patients and caregivers) what l**eads to the imperative of considering their requirements separately.**

Curtis KE, Lahiri S, Brown KE. Targeting Parents for Childhood Weight Management: Development of a Theory-Driven and User-Centered Healthy Eating App. *JMIR Mhealth Uhealth*. 2015;3(2):e69. doi:10.2196/mhealth.3857, pmid:26088692

Every effort was made to carefully facilitate focus groups, but it is possible that **one or two people may have dominated the conversation**, or that unknown organizational hierarchies limited the contribution of some individuals relative to others.

Carr EC, Babione JN, Marshall D. Translating research into practice through user-centered design: An application for osteoarthritis healthcare planning. *Int J Med Inform*. 2017;104:31-37. doi:10.1016/j.ijmedinf.2017.05.007, pmid:28599814

Designer bias is difficult to overcome, even when UCD methods are used to collect contradictory evidence. The sequence of design following formative research means

some assumptions are not tested or contradicted until the testing phase, by which time the assumptions may have greatly influenced the design.

Furthermore, designers should **be judicious in the use of design techniques, such as personas, which can lead to oversimplification** and encourage misleading assumptions about end users [82].

In our experience, designers, clinician stakeholders, and patient stakeholders were divided on what was possible for and needed from the product being designed. Generally, clinicians were more conservative, preferring to replicate existing practices and avoid less studied or riskier options. For instance, clinicians were more conservative than designers about how much unedited information and control over its interpretation to offer patients. Another point of contention was whether to integrate the product into other health information systems, including electronic medical records. Patients preferred integration, whereas designers were divided on leveraging those systems at the expense of their practical limitations and regulatory constraints**. Innovation also conflicted with clinical** reality, a case where a patient or designer might envision something that is not technically possible or clinically relevant [32].

For example, the design team assumed an ability to predict heart failure events through CIED data that were beyond publicly available scientific knowledge. **Designers' innovative ideas could also be mismatched with what patient end users were used to and could comfortably perform.** This may have been the case with patients' dislike of rewards or reluctance to rate their health using standard online rating conventions (eg, out of 5 stars). In general, end users tend to have more conventional preferences than designers [26]. In mHealth projects, patients may be unaware of or reluctant to suggest all the technological possibilities granted by smartphones [29], such as push notifications [84] or smartphone sensors [27].

In conversations with innovators, UCD professionals often hear the statement attributed to Henry Ford, **"If I had asked people what they wanted, they would have said faster horses." The broader challenge is maintaining the innovation equilibrium: allowing innovators to innovate, while also allowing stakeholders to influence or evaluate their design, especially when it comes to usability, safety, and privacy.**

what Cornet et al [32] call type 2 design error, which **"occurs when designers do not accommodate the clinical reality, including biomedical knowledge, clinical workflows, and organizational requirements."**

Although standardized methods ensure scientific reproducibility, rigidity in the UCD process can undermine the goal of iteratively improving a product, which often requires flexibility and experimentation [65]. I

Cornet VP, Toscos T, Bolchini D, et al. Untold Stories in User-Centered Design of Mobile Health: Practical Challenges and Strategies Learned From the Design and Evaluation of an App for Older Adults With Heart Failure. *JMIR Mhealth Uhealth*. 2020;8(7):e17703. doi:10.2196/17703, pmid:32706745

We identified these same concerns in our qualitative research, **which reinforced the fact that an ICT tool that replicates current social media forums is both inadequate and potentially dangerous**. A social networking tool on an ICT application for victims of violence needs features to create secured, anonymous, and moderated conversation forums.

Patel D, Sarlati S, Martin-Tuite P, et al. Designing an Information and Communications Technology Tool With and for Victims of Violence and Their Case Managers in San Francisco: Human-Centered Design Study. *JMIR Mhealth Uhealth*. 2020;8(8):e15866. doi:10.2196/15866, pmid:32831179

Nevertheless, all study phases involved potential users, following key guiding participatory design principles such as . A more targeted approach allowed us to focus on a tangible solution to increase potential success with academic grants. This approach combined a substantial number of tools and techniques into a coherent design process. Applicable results were pursued to move relatively quickly and test a solution in a clinical context.

Yet, welcoming the expression of all needs during designing (ie, explicit, observable, tacit and latent) and looking for what people say, do, make, test, and dream [25,33] poses the challenge of **prioritizing** the (endless) possibilities during a (non-eternal) research study.

One study limitation results from the fact that stakeholders were not left to fend for themselves if a problem occurred during usability testing.

Guay M, Labbé M, Séguin-Tremblay N, et al. Adapting a Person’s Home in 3D Using a Mobile App (MapIt): Participatory Design Framework Investigating the App's Acceptability. *JMIR Rehabil Assist Technol*. 2021;8(2):e24669. doi:10.2196/24669, pmid:33973867

Several challenges were inevitably experienced during the design process. As expected, the psychologists struggled at times to receive feedback that could be used to change the content more specifically. This mostly occurred when the PRPs did not feel like they related to that specific section of the intervention. However, since no group of patients is homogenous, it was not unexpected that the group would present with diverse problems and experiences.

Humphries SM, Rondung E, Norlund F, et al. Designing a Web-Based Psychological Intervention for Patients With Myocardial Infarction With Nonobstructive Coronary Arteries: User-Centered Design Approach. *J Med Internet Res*. 2020;22(9):e19066. doi:10.2196/19066, pmid:32940615

When involving patients and clinicians in a participatory design process, **it is important to be aware of the fact that they are engaged for different reasons and with different agendas**. For the women in our study, the overarching goal of participating in the design and development phase was to become more familiar with their disease. For the healthcare professionals, in contrast, the primary goal was to be part of the transformation of healthcare services. However, it is important to be aware of the relevance of engaging all stakeholders in the field, and that users are not only patients but also healthcare professionals

Ravn Jakobsen P, Hermann AP, Søndergaard J, Wiil UK, Clemensen J. Development of an mHealth Application for Women Newly Diagnosed with Osteoporosis without Preceding Fractures: A Participatory Design Approach. *Int J Environ Res Public Health*. 2018;15(2). doi:10.3390/ijerph15020330, pmid:29438343

For instance, during the co-design workshop, multiple participants talked about how **one's strengths were a very personal thing** and something one might not want to share with anyone. Similarly, in the idea-generating workshop, several participants also voiced concerns about **privacy** and suggested using nicknames in communication with others. Clearly not wanting to share this information about themselves, one participant simply said, **"I would never put something like this on Facebook"** As such, we can surmise that although people living with chronic illnesses, for instance, often are active in support and interest groups on social media [79], they should also likely be the ones to control what, if anything, to share.

Fourth, regulations concerning privacy and data security made us decide to drop the inclusion of social functionalities in the MyStrengths app. Although necessary for us to do, this also meant that we discarded one of the more popular features suggested by participants throughout all our activities. Similarly, because of unforeseen challenges at our end as well as for our external collaborators, we were never able to implement the redesign of the daily log part of the app.

Jessen S, Mirkovic J, Nes LS. MyStrengths, a Strengths-Focused Mobile Health Tool: Participatory Design and Development. *JMIR Form Res*. 2020;4(7):e18049. doi:10.2196/18049, pmid:32706651

Finally , in alignment with the design thinking step 1 of understanding and empathizing with the audience, we put the primary focus on **ensuring all stakeholders felt a part of the process and opened up about their experiences without feeling judged.**

Marko-Holguin M, Cordel SL, Van Voorhees BW, et al. A Two-Way Interactive Text Messaging Application for Low-Income Patients with Chronic Medical Conditions: Design-Thinking Development Approach. *JMIR Mhealth Uhealth*. 2019;7(5):e11833. doi:10.2196/11833, pmid:31042152

We identified active membership in the CoP as a vehicle for the young adults users to re-discover themselves as somebody who "can do it" and someone with "power". In this sense, the CoP served as the basis of an **'identity changer' from being a 'receiver' to**

being a 'giver' or from being a patient in need, to being a designer of need. The study illuminated that learning in a CoP does not 'just happen'. Rather learning is an assisted and managed practice that heavily depends on a fertile environment as well as generative tools and techniques for generating insights and co-constructing knowledge.

Terp M, Laursen BS, Jørgensen R, Mainz J, Bjørnes CD. A room for design: Through participatory design young adults with schizophrenia become strong collaborators. *Int J Ment Health Nurs*. 2016;25(6):496-506. doi:10.1111/inm.12231, pmid:27293176

Methodological Misalignment

It was encouraging that participants liked our mock-up design concept and provided positive feedback. **However, the results of the first iteration of our UCD process revealed several challenges and opportunities to achieve an effective and easy to use tracking system for those caring for children with AD.**

Kim J-E, Lee J, Kwon H, Bessho M, Sakamura K. Towards Supporting Childhood Atopic Dermatitis Management: A User-Centered Design Approach. In: *2020 IEEE 33rd International Symposium on Computer-Based Medical Systems (CBMS)*. ieeexplore.ieee.org; 2020:368-373. http://dx.doi.org/10.1109/CBMS49503.2020.00075

However, with **disciplinary diversity comes disagreement, communication difficulty, and differences in assumptions**, and although these are all desirable elements, they require efforts to manage, for example, by frequently asking team members to state their assumptions.

Multidisciplinary collaboration is often encouraged in UCD [6,65,66], including partnerships between designers and clinicians [67,68]. Those who have attempted such collaborations are aware of the **methodological and cultural misalignmen**t or divergent goals between Human-Computer Interaction technologists and clinicians [65,69].

Cornet VP, Toscos T, Bolchini D, et al. Untold Stories in User-Centered Design of Mobile Health: Practical Challenges and Strategies Learned From the Design and Evaluation of an App for Older Adults With Heart Failure. *JMIR Mhealth Uhealth*. 2020;8(7):e17703. doi:10.2196/17703, pmid:32706745

For patients, their own familiarity with technology, alongside access to a computer and the Internet, was a barrier. For health professionals supporting the introduction and use of PainCheck in the community, barriers included a lack of confidence and familiarity with PainCheck, and HIT generally, which influenced decision making around whether they introduced the system to patients.

The development of PainCheck highlighted **a tension between the continuous, iterative development of HIT systems by software developers and the controlled processes of formal evaluation in research.** Approaches to evaluation that incorporate, for example, **randomized controlled trials** are only recommended when the intervention and its delivery package are stable. These can be implemented with high fidelity, and there is a reasonable likelihood that the overall benefits will be clinically meaningful (ie, improved outcomes or equivalent outcomes at less cost). Within current clinical trial design, there is not sufficient scope for ongoing, iterative development of HIT-based interventions. This issue requires attention to ensure that the develop- ment and evaluation of e-health tools for cancer care keep pace with efforts to increase the use of ever-evolving HIT systems.

Allsop MJ, Johnson O, Taylor S, et al. Multidisciplinary Software Design for the Routine Monitoring and Assessment of Pain in Palliative Care Services: The Development of PainCheck. *JCO Clin Cancer Inform*. 2019;3:1-17. doi:10.1200/CCI.18.00120, pmid:31577449

Insights generated from interacting with a variety of stakeholders, not just health care providers, highlighted **contextual barriers** that affect nomadic CHVs and the recurring challenges that influence the supply chain system generally.

Andersson SR, Hassanen S, Momanyi AM, et al. Using Human-Centered Design to Adapt Supply Chains and Digital Solutions for Community Health Volunteers in Nomadic Communities of Northern Kenya. *Glob Health Sci Pract*. 2021;9(Suppl 1):S151-S167. doi:10.9745/GHSP-D-20-00378, pmid:33727327

For example, one group suggested awarding 100 points for each of the 8 completed small tasks during a day, for a total of 800 points as a score for completion. Although rewarding points are one of the more popular gameful design elements [6,7], it is also known that one does not engage users more by inflating the rewards by as suggested, giving 100 instead of a single point [17]. **Therefore, even though involving users is both important and valuable, one must still make sure design decisions are made in accordance with relevant literature and evidence concerning both the design and content of the tool that is being made.** However, giving the participants the freedom to interpret their own tasks also allowed them to veer in directions that can be unproductive (as with the example of awarding 100 points at a time) or impossible to implement. At the same time, it is hard to correct participants when they veer outside our topic without seeming critical or negative, and in these few cases, we mostly let them continue.

Jessen S, Mirkovic J, Ruland CM. Creating Gameful Design in mHealth: A Participatory Co-Design Approach. *JMIR Mhealth Uhealth*. 2018;6(12):e11579. doi:10.2196/11579, pmid:30552080

The current platform also prioritizes certain clinician and researcher needs. First, Clinicians meeting a patient with long-standing MS must often spend valuable encounter time reviewing copious historical and administrative records to extract key MS-related clinical information. Second, Neurologists who are not MS experts also benefit from benchmarking a given patient's current function against the UCSF MS

research cohort, a form of virtual cohort to inform expectations about patient course. Third, patients who are more informed about treatment decisions, such as weighing risks and benefits of therapies or what to expect at various life stages, are better able to participate in the clinical decision-making process.

Schleimer E, Pearce J, Barnecut A, et al. A Precision Medicine Tool for Patients With Multiple Sclerosis (the Open MS BioScreen): Human-Centered Design and Development. *J Med Internet Res*. 2020;22(7):e15605. doi:10.2196/15605, pmid:32628124

Cultural Sensitivity

Keywords: cultural preservation, relevance, (avoiding) addictiveness, cultural variation

Defining the target user population and understanding their device requirements early in the design and development process may resolve acceptability issues and minimize the need for significant design changes later in the process [90]. This approach has the potential to not only mitigate issues of acceptability and usability but also reduce the impact on development time and costs, while simultaneously increasing the chances of successful adoption and sustained use of the technology [45,91].

Alder G, Taylor D, Rashid U, et al. A Brain Computer Interface Neuromodulatory Device for Stroke Rehabilitation: Iterative User-Centered Design Approach. *JMIR Rehabil Assist Technol*. 2023;10(1). https://www.scopus.com/inward/record.uri?eid=2-s2.0-85180942123&doi=10.2196%2f49702&partnerID=40&md5=971587da81ea3e6deaa629234db24c7c

In identifying the way the HopScotch game would look like, emphasis was made to ensure it is **culturally-preserving and relevant**. Most Himba communities have limited infrastructure including a lack of electricity.

Abubakar_A_Gamifying the unspoken Designing to Resolve Myths on Contraceptives among the Himba people of Namibia.pdf. http://dx.doi.org/10.1145/3334480.XXXXXXX

Participants indicated that they wanted information using a variety of formats (text, visuals, audio, and video) to address sensory needs (eg, vision and hearing deficits) and to be culturally sensitive.

Donald M, Beanlands H, Straus SE, et al. A Web-Based Self-Management Support Prototype for Adults With Chronic Kidney Disease (My Kidneys My Health): Co-Design and Usability Testing. *JMIR Form Res*. 2021;5(2):e22220. doi:10.2196/22220, pmid:33560245

In eastern culture, people use a circle to represent the notion that everything is copasetic. Women with breast cancer may use the notion to pray for a smooth process during their cancer treatment. The design thinking approach and use of the illustration activity helped discover the user's interface preferences and increased the programmer's sensitivity to their needs.

Hou I-C, Lan M-F, Shen S-H, et al. The Development of a Mobile Health App for Breast Cancer Self-Management Support in Taiwan: Design Thinking Approach. *JMIR Mhealth Uhealth*. 2020;8(4):e15780. doi:10.2196/15780, pmid:32352390

Still, through activities involving both users and experts, it was repeatedly suggested to be cautious with such elements, and during the co-design workshops, **users voiced a specific dislike for designs with reward schemes that facilitate addictive use.**

We had to abandon the implementation of social features, primarily because of restrictions from the privacy officers at our institution. Although we could have foreseen this issue and removed the possibility of social features in our activities, we might have restricted the participants' creativity and range of possibilities. Furthermore, by working without restrictions during the workshops, participants also give important information and feedback that, for instance, are relevant not only to social features but also to mHealth tools as a whole. It may seem more efficient concerning time and money to keep participatory activities focused on what is possible or advisable to create. However, we still recommend allowing for free creativity and ideation in such activities, as this can yield not only interesting ideas and concepts but also valuable insights into the user group and their needs and wishes.

Jessen S, Mirkovic J, Nes LS. MyStrengths, a Strengths-Focused Mobile Health Tool: Participatory Design and Development. *JMIR Form Res*. 2020;4(7):e18049. doi:10.2196/18049, pmid:32706651

Although the MHeC-S was comprehensible to our Colombian participants, many changes were requested. In agreement with other authors [87], we strongly advocate for the need to adapt HITs beyond language by considering cultural variations. The same authors suggest adapting or designing HITs to acknowledge cultural differences in 4 main dimensions: **content, functionality, technology platform, and user interface** [44]. **However, the methodology needed to achieve this has not been conceptualized.**

Ospina-Pinillos L, Davenport TA, Navarro-Mancilla AA, et al. Involving End Users in Adapting a Spanish Version of a Web-Based Mental Health Clinic for Young People in Colombia: Exploratory Study Using Participatory Design Methodologies. *JMIR Ment Health*. 2020;7(2):e15914. doi:10.2196/15914, pmid:32027313

Previous research has highlighted the need to tailor HIT interventions beyond content and language, by including culture [39]. One of the strengths of this study was the incorporation of the cultural framework as a cornerstone of the research and development cycle. As a consequence, we obtained information about the participants' cultural

preferences for the prototype's interface and functionality, as well as the development of culturally appropriate content and features. Performing data collection and analysis in the original language reduced the risk of losing relevant information (or meaning), and decreased research time and costs [114].

Ospina-Pinillos L, Davenport T, Mendoza Diaz A, Navarro-Mancilla A, Scott EM, Hickie IB. Using Participatory Design Methodologies to Co-Design and Culturally Adapt the Spanish Version of the Mental Health eClinic: Qualitative Study. *J Med Internet Res*. 2019;21(8):e14127. doi:10.2196/14127, pmid:31376271

A similar approach to testing cultural appropriateness, usability, and the need for adaptation of the iCanCope with Pain app has recently been undertaken in Norway [32], with preliminary outcomes indicating high levels of acceptability and usability, the only adaptations being the need for optimizing user interaction of the social support feature [32].

Slater H, Stinson JN, Jordan JE, et al. Evaluation of Digital Technologies Tailored to Support Young People's Self-Management of Musculoskeletal Pain: Mixed Methods Study. *J Med Internet Res*. 2020;22(6):e18315. doi:10.2196/18315, pmid:32442143

We recognize that the cultural diversity of individuals speaking other languages may correspond with different needs that the app will need to meet. Future research may address these limitations.

Tobias G, Spanier AB. Developing a Mobile App (iGAM) to Promote Gingival Health by Professional Monitoring of Dental Selfies: User-Centered Design Approach. *JMIR Mhealth Uhealth*. 2020;8(8):e19433. doi:10.2196/19433, pmid:3279598

It was also evident in the prototype testing that participants struggled to grasp the overall concept of the app before it was explicitly described. This may be explained by the fact that the participants were presented the pilot app somewhat out of context; for example, it was not suggested by a friend or health worker as might be the case in a real-world scenario. **This suggests that dissemination of future apps needs to be contextually embedded, with many potential avenues available.**

Tonkin E, Jeffs L, Wycherley TP, et al. A Smartphone App to Reduce Sugar-Sweetened Beverage Consumption Among Young Adults in Australian Remote Indigenous Communities: Design, Formative Evaluation and User-Testing. *JMIR Mhealth Uhealth*. 2017;5(12):e192. doi:10.2196/mhealth.8651, pmid:29233803

As has been shown in previous research, poor eHealth literacy and acceptance could lead to disparities [53]. In particular, factors such as age and disabilities have been negatively associated with the digital divide [54]. The median age of PD onset is 60 years [2]. In comparison, the median age of the PwP in our study was 73 years.

Verhoeven F, Cremers A, Schoone M, Van Dijk J. Mobiles for mobility: Participatory design of a "Happy walker" that stimulates mobility among older people. *Gerontechnology*. 2016;15(1). https://journal.gerontechnology.org/currentIssueContent.aspx?aid=2248

Cost and Scale

Keywords: more participants, field visits,

Funding could be made available for local and international researchers and students to continue the iterative process on the project and fulfil the vision of widespread adoption and use.

Abubakar_A_Gamifying the unspoken Designing to Resolve Myths on Contraceptives among the Himba people of Namibia.pdf. http://dx.doi.org/10.1145/3334480.XXXXXXX

Keywords: scale, support services, mHealth integration into health training, blind spots, rapid testing vs longitudinal study, balancing wants and needs

Lastly, future work is necessary in simulating similar, repeated events at a larger scale to encourage open discourses and discussions on patient information systems designed specifically for children.

Aufegger L, Bùi KH, Bicknell C, Darzi A. Designing a paediatric hospital information tool with children, parents, and healthcare staff: a UX study. *BMC Pediatr*. 2020;20(1):469. doi:10.1186/s12887-020-02361-w, pmid:33032549

To address healthcare providers' concerns of their own preparedness and workload capacity, healthcare sys- tems should consider developing **support services** and resources surrounding mHealth and PGD integration, such as topic-specific education.

Brox E, Konstantinidis ST, Evertsen G. User-Centered Design of Serious Games for Older Adults Following 3 Years of Experience With Exergames for Seniors: A Study Design. *JMIR Serious Games*. 2017;5(1):e2. doi:10.2196/games.6254, pmid:28077348

However, we recognize that in the health service context, **attempts to apply PD have been mainly restricted to the design of small-scale isolated systems for a limited set of users in a specific organizational unit** [10], with a call for larger-scale PD approaches.

Calvillo-Arbizu J, Roa-Romero LM, Estudillo-Valderrama MA, et al. User-centred design for developing e-Health system for renal patients at home (AppNephro). *Int J Med Inform*. 2019;125:47-54. doi:10.1016/j.ijmedinf.2019.02.007, pmid:30914180

Although we recruited a diverse group of participants, we may have missed some perspectives. One limitation is the absence of **larger scale beta-testing** prior to deployment in Phase 2.

Another challenge of UCD is balancing implementation of suggestions and prolonging the timeline of app development.

Chan NH-M, Merali HS, Mistry N, et al. Development of a novel mobile application, HBB Prompt, with human factors and user-centred design for Helping Babies Breathe skills retention in Uganda. *BMC Med Inform Decis Mak*. 2021;21(1):39. doi:10.1186/s12911-021-01406-z, pmid:33541340

The forms of involvement vary from collecting extensive data to asking individuals to assess early products [65]. The level of involvement can also be adjusted between informing (as in interviews), advising (as in reviewing concepts), and doing (as in having stakeholders co-perform research or design work) [71]. **However, more active or laborious stakeholder involvement risks asking individuals to do more than what is realistic, reasonable, or affordable** [21,72]. **This is often the case when individuals are asked to be co-designers without adequate training in design, compensation for their contribution, or understanding of the problem space. Although some involvement is essential to UCD, more is not always better**

Although stakeholder involvement is essential to UCD [1,76], it is predicated on stakeholders having unique knowledge or insights that designers do not have. **However, stakeholders too have limited knowledge and represent primarily the communities to which they belong, meaning even with stakeholder involvement, there may exist multiple blind spots.**

In practice, however, **few design studies have the opportunity to conduct formative research with large samples** representative of the population, whereas increasing sample size exacerbates formative research challenge 1 ("When and how much to involve stakeholders"), as discussed above.

Participants spent 30 min using a prototype technology meant to be used for weeks, months, and years. They were then asked to project how they would use the technology in practice.

Laboratory evaluation is appropriate to quickly iterate on designs and address usability issues before in-the-wild testing to avoid fielding a poorly designed product. However, **in-the-wild testing is expensive and time-intensive and may not be possible in every project.**

Cornet VP, Toscos T, Bolchini D, et al. Untold Stories in User-Centered Design of Mobile Health: Practical Challenges and Strategies Learned From the Design and Evaluation of an App for Older Adults With Heart Failure. *JMIR Mhealth Uhealth*. 2020;8(7):e17703. doi:10.2196/17703, pmid:32706745

Consequently , by the time mHealth interventions are implemented and tested, the technology may have potentially moved on. Secondly, systematically developing a health promotion app intervention can also be resource intensive. In this study,

incorporating UCD revealed the need for further refinement of app features which is a much needed step. At the same time, implementing the changes requires additional resources.

Curtis KE, Lahiri S, Brown KE. Targeting Parents for Childhood Weight Management: Development of a Theory-Driven and User-Centered Healthy Eating App. *JMIR Mhealth Uhealth*. 2015;3(2):e69. doi:10.2196/mhealth.3857, pmid:26088692

First, developing an mHealth intervention by deploying UCD methodology is rather time- and resource-intensive [9-47]. Some refinements of the features developed need to be implemented before a future large-scale deployment of our app, and going through the different phases of our intervention design took much longer than the typical design time frame of commercial apps for health promotion [48].

Gabrielli S, Dianti M, Maimone R, et al. Design of a Mobile App for Nutrition Education (TreC-LifeStyle) and Formative Evaluation With Families of Overweight Children. *JMIR Mhealth Uhealth*. 2017;5(4):e48. doi:10.2196/mhealth.7080, pmid:28408361

For example, **the request of OTs to be able in the future to delete, add, or move** architectural elements (eg, cabinets in the bathroom) on a scan **must be balanced with the cost of such a technological development,** in a context where other available technologies already address this need [16-21].

Guay M, Labbé M, Séguin-Tremblay N, et al. Adapting a Person's Home in 3D Using a Mobile App (MapIt): Participatory Design Framework Investigating the App's Acceptability. *JMIR Rehabil Assist Technol*. 2021;8(2):e24669. doi:10.2196/24669, pmid:33973867

Environment and Context

Untested In-Situ

Keywords: community, context, generalisability, environment

First, we were unable to visit the community due to resource challenges, we only came about the participants through a conference. **A visit to the community will have added more value to the process.**

Abubakar_A_Gamifying the unspoken Designing to Resolve Myths on Contraceptives among the Himba people of Namibia.pdf. http://dx.doi.org/10.1145/3334480.XXXXXXX

User-centered design should focus on the **context of use,** functionality, terminology, and user interface evaluation for mHealth applications.

Afrizal SH, Hidayanto AN, Hakiem N, Sartono A, Priyambodo S, Eryando T. Design of mHealth Application for Integrating Antenatal Care Service in Primary Health Care: A User-Centered Approach. *2019 Fourth International Conference on Informatics and Computing (ICIC)*. Published online 2019. http://dx.doi.org/10.1109/icic47613.2019.8985911

Context that helps patients understand what to do with their data is important. Over the course of treatment, patients had experienced different situations that guided their infor- mation needs, such as life-saving high-voltage shocks without a warning or shocks in error when not necessary.

Ahmed R, Toscos T, Rohani Ghahari R, et al. Visualization of Cardiac Implantable Electronic Device Data for Older Adults Using Participatory Design. Appl Clin Inform. 2019;10(4):707-718. doi:10.1055/s-0039-1695794, pmid:31533172

Consideration therefore needed to be given to whether the competing priorities of 'sharing thoughts' and RPV could co-exist in one system. In order to successfully merge with an existing system (RPV), **careful consideration needs to be given to the environment and context in which it currently exists.** Therefore, future work should consider the socio-political context in which these systems exist and are developed within (Kincheloe & McClaren 2000). Although the IT experts were engaged with the design process, 'sharing thoughts' was one of a number of studies with which they were involved. We, therefore, suggest that researchers need to consider this during the initial planning stage to ensure that IT experts are able to play a more central role in the collaborative process.

Giles SJ, Reynolds C, Heyhoe J, Armitage G. Developing a patient-led electronic feedback system for quality and safety within Renal PatientView. *J Ren Care*. 2017;43(1):37-49. doi:10.1111/jorc.12186, pmid:27990782

Second, the usability of Pain Squad+ in adolescent environments other than the hospital (eg, home and school) and longitu- dinally was not assessed.

Jibb LA, Cafazzo JA, Nathan PC, et al. Development of a mHealth Real-Time Pain Self-Management App for Adolescents With Cancer: An Iterative Usability Testing Study [Formula: see text]. *J Pediatr Oncol Nurs*. 2017;34(4):283-294. doi:10.1177/1043454217697022, pmid:28376666

We acknowledge that the following limitations exist regarding this study: 1) Tic–Tac-Training was playtested only at one work- place by adults, thus we do not know how it is received at other workplaces (or between workplaces) and user groups (e.g., chil- dren and seniors);

Laine TH, Normark J, Lindvall H, Lindqvist A-K, Rutberg S. A Distributed Multiplayer Game to Promote Active Transport at Workplaces: User-Centered Design, Implementation, and Lessons Learned. *IEEE Trans Comput Intell AI Games*. 2020;12(4):386-397. http://dx.doi.org/10.1109/TG.2020.3021728

Additional research is also needed to develop the MHeC-C and test its engagement, efficacy, and effectiveness in real-world settings and engage other stakeholders, such as administration and management, peers, nongovernmental organizations, other community organizations, and senior health professionals with diverse degrees of technology literacy.

Ospina-Pinillos L, Davenport TA, Navarro-Mancilla AA, et al. Involving End Users in Adapting a Spanish Version of a Web-Based Mental Health Clinic for Young People in Colombia: Exploratory Study Using Participatory Design Methodologies. *JMIR Ment Health*. 2020;7(2):e15914. doi:10.2196/15914, pmid:32027313

Further research is needed to understand the acceptability and usability of the system, as well as to validate all the components in real-world settings.

Ospina-Pinillos L, Davenport TA, Ricci CS, Milton AC, Scott EM, Hickie IB. Developing a Mental Health eClinic to Improve Access to and Quality of Mental Health Care for Young People: Using Participatory Design as Research Methodologies. *J Med Internet Res*. 2018;20(5):e188. doi:10.2196/jmir.9716, pmid:29807878

Importantly, additional steps are needed to evaluate the engagement, efficacy, and effectiveness of the MHeC-S in real-world settings.

Ospina-Pinillos L, Davenport T, Mendoza Diaz A, Navarro-Mancilla A, Scott EM, Hickie IB. Using Participatory Design Methodologies to Co-Design and Culturally Adapt the Spanish Version of the Mental Health eClinic: Qualitative Study. *J Med Internet Res*. 2019;21(8):e14127. doi:10.2196/14127, pmid:31376271

The features of the ICT tool we have described here are specific to our population and environment and represent early prototypes that will be iterated on in the next phase of the design process. The wireframes and their associated features are preliminary ideas developed by the design researchers in order to represent the design opportunity visually. The content of the wireframes is based on quotes and insights from our users. These wireframes have not been tested with users.

Patel D, Sarlati S, Martin-Tuite P, et al. Designing an Information and Communications Technology Tool With and for Victims of Violence and Their Case Managers in San Francisco: Human-Centered Design Study. *JMIR Mhealth Uhealth*. 2020;8(8):e15866. doi:10.2196/15866, pmid:32831179

It is also important to highlight that the participants' views were based on only visualizing a prototype intervention aimed to be delivered during holidays.

It would have been useful to have a group of users that tried out the app on their own, followed by an interview about their experiences (ie process evaluation built into a pilot acceptability and feasibility study). This would provide further insight into how people perceived and used the app in their own time, which may be different from when a researcher is present [49].

Rodrigues AM, Sniehotta FF, Birch-Machin MA, Olivier P, Araújo-Soares V. Systematic and Iterative Development of a Smartphone App to Promote Sun-Protection Among Holidaymakers: Design of a Prototype

and Results of Usability and Acceptability Testing. *JMIR Res Protoc*. 2017;6(6):e112. doi:10.2196/resprot.7172, pmid:28606892

Apart from this, the application has not been implemented and tested in real-life situations, which will be the next step of our research to answer questions on the outcome (e.g., correctness of the documentation) and the performance of the system.

Sedlmayr B, Schöffler J, Prokosch H-U, Sedlmayr M. User-centered design of a mobile medication management. *Inform Health Soc Care*. 2019;44(2):152-163. doi:10.1080/17538157.2018.1437042, pmid:29504838

Several lessons were learned during the UCD process that can be transferable for the development of other clin- ical systems (RQ2). Firstly, the creation of clinical systems requires active involvement of all target user groups in the design and continuous evaluation of the solution and, when possible, in a high-fidelity simulation environment that realistically recreates the context of use.

Smaradottir B, Gerdes M, Martinez S, Fensli R. The EU-project United4Health: User-centred design of an information system for a Norwegian telemedicine service. *J Telemed Telecare*. 2016;22(7):422-429. doi:10.1177/1357633X15615048, pmid:26541347

For the RQ2 about the lessons applicable in real-world scenarios, the study has shown that a fully-implemented system based on the prototype presented, potentially avoids the risks associated to paper-based procedures.

Instead, this study might be seen as a necessary step for the validation of the controlled conditions that should be carried out before the use of the system in real clinical settings.

Smaradottir B, Holen-Rabbersvik E, Thygesen E, Fensli R, Martinez S. User-centred design of the user interface of a collaborative information system for inter-municipal dementia team. In: *Proceedings of the International Conference on Health Informatics*. SCITEPRESS - Science and and Technology Publications; 2015. http://www.scitepress.org/DigitalLibrary/Link.aspx?doi=10.5220/0005222704460453

Although the intervention program was developed using a participatory design approach to support the likelihood of acceptability, usability, and feasibility, acceptability will need to be further tested and established in a future pilot test study before instigating efficacy studies.

Ledel Solem IK, Varsi C, Eide H, et al. A User-Centered Approach to an Evidence-Based Electronic Health Pain Management Intervention for People With Chronic Pain: Design and Development of EPIO. *J Med Internet Res*. 2020;22(1):e15889. doi:10.2196/15889, pmid:31961331

A limitation of this study is that we did not assess performance of the exercises to check patients were performing them correctly and we do not know if including modifying the SARAH programme exercises for the mobile app results in the same clinical out- comes as the full SARAH programme.

Tonga E, Williamson E, Srikesavan C, Özen T, Sarıtaş F, Lamb SE. A hand exercise mobile app for people with rheumatoid arthritis in Turkey: design, development and usability study. *Rheumatol Int*. 2021;41(6):1151-1160. doi:10.1007/s00296-021-04860-0, pmid:33870452

In addition, the evaluation was conducted in a **controlled environment,** which is not fully representative of the final intended user environment.

Future testing should involve participants taking the system home, enabling the collection of user satisfaction data based on use in an actual home setting.

Uddin AA, Morita PP, Tallevi K, et al. Development of a Wearable Cardiac Monitoring System for Behavioral Neurocardiac Training: A Usability Study. *JMIR Mhealth Uhealth*. 2016;4(2):e45. doi:10.2196/mhealth.5288, pmid:27106171

A limitation of this study is that the prototyped eHealth service has not been implemented and evaluated in clinical practice. Thus, our study does not allow us to draw conclusions about the actual value of the desired eHealth functionalities for co-care, including cost-effectiveness and clinical outcomes.

Verhoeven F, Cremers A, Schoone M, Van Dijk J. Mobiles for mobility: Participatory design of a “Happy walker” that stimulates mobility among older people. *Gerontechnology*. 2016;15(1). https://journal.gerontechnology.org/currentIssueContent.aspx?aid=2248

Multiplicity of User Types

Keywords: passive users, user types, informal carers, portability, transferability, generalisability

Our study has shown that older people must be included in the analysis and design of their own in-home interface to incorporate their contextual knowledge, preferences, and needs. Co-design methods have been incorporated in many studies to investigate older people’s perceptions of smart home technologies. However, it may be time to consider older people as designers rather than simply informers.

Future work could involve health care professionals and relatives of end users in the design process; investigate accessibility features, such as voice control and sound alerts; and explore the next steps in the design of the interface and evaluate such a system in situ. We would also encourage future research to consider how to maximize inclusion and diversity in the research design and potential participants, in terms of sociodemographic characteristics, ethnicity, sex, and gender, as well as the cognition and experience of technology.

Ghorayeb A, Comber R, Gooberman-Hill R. Development of a Smart Home Interface With Older Adults: Multi-Method Co-Design Study. *JMIR Aging*. 2023;6.

https://www.scopus.com/inward/record.uri?eid=2-s2.0-85164453372&doi=10.2196%2f44439&partnerID=40&md5=626088a361c675c6599b6a11b3eb73e0

We suggest that we do not just focus on designing for the relative as a user but also explore designing for relatives with other roles and responsibilities (eg, relatives managing a career and relatives with young children). We need to make sure that we develop online support that allows relatives to retain their identity rather than just focusing on caring.

Honary M, Fisher NR, McNaney R, Lobban F. A Web-Based Intervention for Relatives of People Experiencing Psychosis or Bipolar Disorder: Design Study Using a User-Centered Approach. *JMIR Ment Health*. 2018;5(4):e11473. doi:10.2196/11473, pmid:30530457

First, informal carers were less involved in the pilots. However, **the role of informal carers** is becoming in- creasingly relevant and they are also able to stimulate the acceptance of technology by people with disabili- ties [36]. Scherer et al. (2005) stress it is also important to consider the user's environment and support system as key elements for the successful integration of assis- tive technologies [37].

Kerkhof YJF, den Ouden MEM, Soeteman S, Scholten A, Ben Allouch S, Willems CG. Development of a memory application for structuring and supporting daily activities of clients with intellectual disabilities. *Technol Disabil*. 2017;29(1-2):77-89. https://www.medra.org/servlet/aliasResolver?alias=iospress&doi=10.3233/TAD-160164

Then designing a mobile app or a game promoting health behavior change, it is important to connect the mechanisms to an appropriate theory to be successful. Hoj et al. [43] found that apps that were built on behavior change theories had a more favorable impact on self-reported app engagement. Bandura's [44] social cognitive theory describes several principal functions that operates on the self-regulative mechanism; self-monitoring, goal-setting of one's behavior, judgement of one's behavior and affective self-reaction. Self-monitoring and goal setting have been found to be beneficial for assisting health behavior change bymobileapplicationinterventions[45],[46].Inaddition,Epton et al. [47] stated that setting group goals is favored, especially when it is monitored by another person. Therefore, designing a game that favor group goals and allows other players (both team- mates and opponents) to monitor the progress could be favor- able to receive behavior change. Although Tic–Tac-Training's design is not strictly based on a specific behavior change theory, its gamification features are aligned with the aforementioned functions, such as group goals, progress/self-monitoring, and goal-setting (customized tasks).

Laine TH, Normark J, Lindvall H, Lindqvist A-K, Rutberg S. A Distributed Multiplayer Game to Promote Active Transport at Workplaces: User-Centered Design, Implementation, and Lessons Learned. *IEEE Trans Comput Intell AI Games*. 2020;12(4):386-397. http://dx.doi.org/10.1109/TG.2020.3021728

This test result provides a solid basis for a subsequent test of the application under real-life conditions, associated with the high stress of an emergency situation.

The development of the cognitive aid needs to focus primarily on the main user of the application, which in the context of the German health care system would be the physician anesthesiologist. In contrast, anesthesia nurses as part of the anesthesia team play an assisting role and are considered as secondary users. However, the usage requirements identified were largely identical for both user groups. Major differences between both user groups only exist with regard to the handling of the cognitive aid.

Schild S, Sedlmayr B, Schumacher A-K, et al. A Digital Cognitive Aid for Anesthesia to Support Intraoperative Crisis Management: Results of the User-Centered Design Process. *JMIR Mhealth Uhealth*. 2019;7(4):e13226. doi:10.2196/13226, pmid:31033445

Secondly, interoperability problems,28,29 are common within clinical environ- ments, so the execution of the field trial provided valuable insights into the interactions between the technologies involved, and a continuous long-term feedback of users' interactions with these technologies

Smaradottir B, Gerdes M, Martinez S, Fensli R. The EU-project United4Health: User-centred design of an information system for a Norwegian telemedicine service. *J Telemed Telecare*. 2016;22(7):422-429. doi:10.1177/1357633X15615048, pmid:26541347

Perceptions of informal caregivers and professionals are important because people with dementia relate to them strongly. They are the key to inclusion and need to take responsibility by taking into account the opinions and preferences of people with dementia [39].

Span M, Hettinga M, Groen-van de Ven L, et al. Involving people with dementia in developing an interactive web tool for shared decision-making: experiences with a participatory design approach. *Disabil Rehabil*. 2018;40(12):1410-1420. doi:10.1080/09638288.2017.1298162, pmid:28286969

Testing

Keywords: more participants, field visits, comprehension, condensed (workshops), conceptual, culture, education, generalisability, (access to) technology,

Lack of Subjects

Keywords: selection bias

A limitation of the study is that the end users who were involved in designing and testing the platform were mHealth researchers; this may limit the generalizability of our findings beyond this population.

Due to our limited sample size during usability testing, **it remains unclear how these features would be used in a larger group of users**. Future studies are warranted.

Liu S, La H, Willms A, Rhodes RE. A “No-Code” App Design Platform for Mobile Health Research: Development and Usability Study. *JMIR Formative Research*. 2022;6(8):e38737. Accessed April 15, 2024. https://formative.jmir.org/2022/8/e38737

Future research should explore usability testing over periods of time with a range of different users.

Alder G, Taylor D, Rashid U, et al. A Brain Computer Interface Neuromodulatory Device for Stroke Rehabilitation: Iterative User-Centered Design Approach. *JMIR Rehabil Assist Technol*. 2023;10(1). https://www.scopus.com/inward/record.uri?eid=2-s2.0-85180942123&doi=10.2196%2f49702&partnerID=40&md5=971587da81ea3e6deaa629234db24c7c

Further research with both quantitative and qualitative design should be conducted to substantiate the findings of the study with diverse populations.

Mak WWS, Ng SM, Leung FHT. A Web-Based Stratified Stepped Care Platform for Mental Well-being (TourHeart+): User-Centered Research and Design. *JMIR Form Res*. 2023;7. https://www.scopus.com/inward/record.uri?eid=2-s2.0-85151375096&doi=10.2196%2f38504&partnerID=40&md5=4e50099456169bcbcf57a54414ad32d5

The study was limited by its small sample size, which limits the generalizability of the findings. However, other studies reported similar sample sizes [75-77].

Brown M, Lord E, John A. Adaptation of ACTivate Your Wellbeing, a Digital Health and Well-being Program for Young Persons: Co-design Approach. *JMIR Form Res*. 2023;7. https://www.scopus.com/inward/record.uri?eid=2-s2.0-85154563972&doi=10.2196%2f39913&partnerID=40&md5=48e49523d172380017982792d2f88e9f

Limitations of this study include generalizability due to the small sample size within a single training environment experiencing a particular instance of telehealth (e.g., rapid expansion during a global pandemic);

Lawrence K, Cho J, Torres C, Alfaro-arias V. Building Virtual Health Training Tools for Residents: A Design Thinking Approach. *Front Digit Health*. 2022;4. https://www.scopus.com/inward/record.uri?eid=2-s2.0-85133449755&doi=10.3389%2ffdgth.2022.861579&partnerID=40&md5=6fefdef880dd090a3360def1c9c291bc

Following consultations with experts in clinical care and experts in technological innovation design, it was decided that we would have more targeted information if we focused on the younger cohort alone, and it was likely that the intervention would have

had different requirements and feedback from older children (13-17 years inclusive). Another study for the older cohort is planned.

More research is required with a larger sample size to evaluate the intervention's usability in the clinical setting.

O'Connor A, Tai A, Brinn M, Thuc Hien Hoang N, Cataldi D, Carson-Chahhoud K. Co-design of an Augmented Reality Asthma Inhaler Educational Intervention for Children: Development and Usability Study. *JMIR Pediatr Parent*. 2023;6. https://www.scopus.com/inward/record.uri?eid=2-s2.0-85167715240&doi=10.2196%2f40219&partnerID=40&md5=cc349ac6ff0a82035b84d2018702298b

Although this study highlights the importance of involving end users in the design process, we acknowledge several potential limitations in our approach. We leveraged highly engaged clinical sites, care teams, and patients and families and used convenience sampling for development and testing; therefore, the perspectives we gained may not be representative of all pediatric rheumatology practices or populations. We engaged 4 diverse clinical sites, including small and large centers, in various locations across the United States. As we proceed to the next phase of building an electronic version of the dashboard, we will have the opportunity to test the dashboard and assess its usability and utility across a larger target population.

Taxter A, Johnson L, Tabussi D, et al. Co-design of an Electronic Dashboard to Support the Coproduction of Care in Pediatric Rheumatic Disease: Human-Centered Design and Usability Testing. *J Particip Med*. 2022;14(1). https://www.scopus.com/inward/record.uri?eid=2-s2.0-85129955234&doi=10.2196%2f34735&partnerID=40&md5=113020f7fb8362b49ba88e848b4a0cd2

A large sample size with a diverse range of stakeholders represented only one employer in the entire sample. Thus, insights from employers are limited, affecting our findings' transferability [60], which is a subject for future research.

Engdahl P, Svedberg P, Lexén A, Tjörnstrand C, Strid C, Bejerholm U. Co-design Process of a Digital Return-to-Work Solution for People with Common Mental Disorders: Stakeholder Perception Study. *JMIR Form Res*. 2023;7. https://www.scopus.com/inward/record.uri?eid=2-s2.0-85148997580&doi=10.2196%2f39422&partnerID=40&md5=f2fd80a075457b2c9d33f2ab2fdaf5bf

Several limitations of the GDM-DH app and its development process are worth mentioning. First, the number of participants in our usability study was limited. Additionally, during usability testing, we may have observed the best-case scenario for comfort and confidence in using the app, leading us to overestimate the true usability and technological proficiency. Second, we did not use a structured framework, such as the Delphi method [75], to organize and structure the discussions to guide our GDM-DH app and intervention development, which will make it difficult for others to replicate our study procedures.

Shanmugavel A, Shakya PR, Shrestha A, et al. Designing and Developing a Mobile App for Management and Treatment of Gestational Diabetes in Nepal: User-Centered Design Study. *JMIR Form Res*. 2024;8. https://www.scopus.com/inward/record.uri?eid=2-s2.0-85193327055&doi=10.2196%2f50823&partnerID=40&md5=4fc125fa968a296014d6ffe224c48538

We acknowledge that this study will **benefit from more participants and field visits** to generalise its findings.

Abubakar_A_Gamifying the unspoken Designing to Resolve Myths on Contraceptives among the Himba people of Namibia.pdf. http://dx.doi.org/10.1145/3334480.XXXXXXX

As the number of participants and the length of the use of the concept were limited in our qualitative research, **broad generalizations** based on the results presented here are not relevant in this phase. However, most of the design implications got support from the findings of both user studies, which strengthen the validity of the results.

Ahtinen A, Andrejeff E, Vuolle M, Väänänen K. Walk as You Work: User Study and Design Implications for Mobile Walking Meetings. In: *Proceedings of the 9th Nordic Conference on Human-Computer Interaction*. NordiCHI '16. Association for Computing Machinery; 2016:1-10. Accessed February 8, 2022. https://doi.org/10.1145/2971485.2971510

There are several limitations to results. Most notably, those involved in the participatory process were **small in number;** experiences or perceptions of the group therefore cannot be guaranteed to **generalize** to the experiences and perceptions of a broader community of users with SCI or SCI stakeholders.

Allin S, Shepherd J, Tomasone J, et al. Participatory Design of an Online Self-Management Tool for Users With Spinal Cord Injury: Qualitative Study. *JMIR Rehabil Assist Technol*. 2018;5(1):e6. doi:10.2196/rehab.8158, pmid:29563075

Findings are limited in terms of its generalisation [58]. While we believe that results can be transferred to different settings within the hospital admission and treatment processes, ranging from general health check-ups to elective surgeries, where hospital information on the infrastructure and roles and responsibilities of healthcare professionals apply irrespective of the type of health care delivered, **future studies are encouraged to gather more in-depth user requirements** [59].

Aufegger L, Bùi KH, Bicknell C, Darzi A. Designing a paediatric hospital information tool with children, parents, and healthcare staff: a UX study. *BMC Pediatr*. 2020;20(1):469. doi:10.1186/s12887-020-02361-w, pmid:33032549

To sum up, although **the number of representatives of end- user groups was limited**, the different tests were carried out successfully, showing that we have accomplished our main goal of creating a useful, intuitive and versatile tool not only for researchers but for clinicians in the different proposed scenarios too.

Ahufinger S, Pequeno E, Herrero P. Mob-EEG: A user-centered design for wireless electroencephalogram in intensive care units. In: *2019 IEEE 32nd International Symposium on Computer-Based Medical Systems (CBMS)*. IEEE; 2019. https://ieeexplore.ieee.org/document/8787513/

First, data were collected from patients treated at academic institutions and members of a community-based organization, all in Memphis, as a convenience sample of adults with SCD, which might limit the **generalizability** of our results to other sickle cell centers.

Alberts NM, Badawy SM, Hodges J, et al. Development of the InCharge Health Mobile App to Improve Adherence to Hydroxyurea in Patients With Sickle Cell Disease: User-Centered Design Approach. *JMIR Mhealth Uhealth*. 2020;8(5):e14884. doi:10.2196/14884, pmid:32383683

Second, the usability testing included a limited number of participants, which is, however, not uncommon for iterative design processes [49]. Based on the above, it is possible that the usability testing of StressProffen so far captured only some of the potential barriers to continuous use over time.

Børøsund E, Mirkovic J, Clark MM, et al. A Stress Management App Intervention for Cancer Survivors: Design, Development, and Usability Testing. *JMIR Form Res*. 2018;2(2):e19. doi:10.2196/formative.9954, pmid:30684438

Our study has several limitations. Despite strong recruitment efforts, several focus groups had fewer participants than expected.

Dannenberg MD, Bienvenida JCM, Bruce ML, et al. End-user views of an electronic encounter decision aid linked to routine depression screening. *Patient Educ Couns*. 2019;102(3):555-563. doi:10.1016/j.pec.2018.10.002, pmid:30497800

Overall limitations of many mHealth applications thus far include limited generalisability of study samples, short study duration (median time=18 months) and **small sample sizes** (median n=28).

Erguera XA, Johnson MO, Neilands TB, et al. WYZ: a pilot study protocol for designing and developing a mobile health application for engagement in HIV care and medication adherence in youth and young adults living with HIV. *BMJ Open*. 2019;9(5):e030473. doi:10.1136/bmjopen-2019-030473, pmid:31061063

Another limitation is that our pilot study involved a small group of participants (parent participants were mainly mothers), which may not reflect the need of other families of overweight children in our target population.

Gabrielli S, Dianti M, Maimone R, et al. Design of a Mobile App for Nutrition Education (TreC-LifeStyle) and Formative Evaluation With Families of Overweight Children. *JMIR Mhealth Uhealth*. 2017;5(4):e48. doi:10.2196/mhealth.7080, pmid:28408361

Working with a larger number of participants who had experienced mental health difficulties or experienced counseling may have strengthened the prototype development.

Gonsalves PP, Hodgson ES, Kumar A, et al. Design and Development of the “POD Adventures” Smartphone Game: A Blended Problem-Solving Intervention for Adolescent Mental Health in India. *Front Public Health*. 2019;7:238. doi:10.3389/fpubh.2019.00238, pmid:31508404

Another limitation comes from sample size. While it includes different user groups, the sample is small which limits generalization.

Guay M, Labbé M, Séguin-Tremblay N, et al. Adapting a Person’s Home in 3D Using a Mobile App (MapIt): Participatory Design Framework Investigating the App's Acceptability. *JMIR Rehabil Assist Technol*. 2021;8(2):e24669. doi:10.2196/24669, pmid:33973867

Furthermore, while the work with PRPs ensured that patients with MINOCA were able to contribute their specific views and experiences towards the intervention, it should be noted that the transferability to current and future patients with MINOCA might be limited due to the sample being recruited from one main city region in Sweden.

Humphries SM, Rondung E, Norlund F, et al. Designing a Web-Based Psychological Intervention for Patients With Myocardial Infarction With Nonobstructive Coronary Arteries: User-Centered Design Approach. *J Med Internet Res*. 2020;22(9):e19066. doi:10.2196/19066, pmid:32940615

First, this study was conducted at a single tertiary pediatric center using a relatively small number of adolescents who were generally newly diagnosed with cancer, which could represent a threat to the generalizability of the results.

Jibb LA, Cafazzo JA, Nathan PC, et al. Development of a mHealth Real-Time Pain Self-Management App for Adolescents With Cancer: An Iterative Usability Testing Study [Formula: see text]. *J Pediatr Oncol Nurs*. 2017;34(4):283-294. doi:10.1177/1043454217697022, pmid:28376666

However, further research with a larger sample and study period is necessary to generalise the results of the final pilot in terms of effectivity of the application.

Kerkhof YJF, den Ouden MEM, Soeteman S, Scholten A, Ben Allouch S, Willems CG. Development of a memory application for structuring and supporting daily activities of clients with intellectual disabilities. *Technol Disabil*. 2017;29(1-2):77-89. https://www.medra.org/servlet/aliasResolver?alias=iospress&doi=10.3233/TAD-160164

Even though our samples were small, we had rich and meaningful discussions.

Marien S, Legrand D, Ramdoyal R, et al. A User-Centered design and usability testing of a web-based medication reconciliation application integrated in an eHealth network. *Int J Med Inform*. 2019;126:138-146. doi:10.1016/j.ijmedinf.2019.03.013, pmid:31029255

Limitations of this work are inherent in the small sample size, and single site investigations in addition to the short time frame for exploitations.

Martin S, Armstrong E, Thomson E, et al. A qualitative study adopting a user-centered approach to design and validate a brain computer interface for cognitive rehabilitation for people with brain injury. *Assist Technol*. 2018;30(5):233-241. doi:10.1080/10400435.2017.1317675, pmid:28708963

Although the number of participants was similar to the sample sizes reported in other related studies, the results were obtained as part of one workshop in one specific context.

Mirkovic J, Jessen S, Kristjansdottir OB, Krogseth T, Koricho AT, Ruland CM. Developing Technology to Mobilize Personal Strengths in People with Chronic Illness: Positive Codesign Approach. *JMIR Form Res*. 2018;2(1):e10774. doi:10.2196/10774, pmid:30684404

This study had four limitations. First, there was a limited number of stakeholders participating in the study (eight patients and four healthcare professionals) and test (four patients) processes.

Naeemabadi M, Søndergaard JH, Klastrup A, et al. Development of an individualized asynchronous sensor-based telerehabilitation program for patients undergoing total knee replacement: Participatory design. *Health Informatics J*. 2020;26(4):2492-2511. doi:10.1177/1460458220909779, pmid:32175788

One of the limitations of this research is the small sample size of participants. .is sample size only allowed us to detect correlations with large effect sizes

Navarro-Alamán J, Lacuesta R, García-Magariño I, Gallardo J, Ibarz E, Lloret J. Close2U: An App for Monitoring Cancer Patients with Enriched Information from Interaction Patterns. *J Healthc Eng*. 2020;2020:3057032. doi:10.1155/2020/3057032, pmid:32733661

The results of MindClimb implementation are derived from a small sample of adolescents and therapists and should be interpreted with this limitation in mind.

Newton A, Bagnell A, Rosychuk R, et al. A Mobile Phone-Based App for Use During Cognitive Behavioral Therapy for Adolescents With Anxiety (MindClimb): User-Centered Design and Usability Study. *JMIR Mhealth Uhealth*. 2020;8(12):e18439. doi:10.2196/18439, pmid:33289671

However, the input from end-users was not possible during every sprint and the total sample size of involved end-users was small.

Noordman J, Driesenaar JA, van Bruinessen IR, van Dulmen S. ListeningTime; participatory development of a web-based preparatory communication tool for elderly cancer patients and their healthcare providers. *Internet Interv*. 2017;9:51-56. doi:10.1016/j.invent.2017.05.002, pmid:30135837

Another limitation was the relatively small sample size, although this number still enabled us to collect sufficient information for an analysis in the framework and reach a saturation point.

Ospina-Pinillos L, Davenport TA, Navarro-Mancilla AA, et al. Involving End Users in Adapting a Spanish Version of a Web-Based Mental Health Clinic for Young People in Colombia: Exploratory Study Using Participatory Design Methodologies. *JMIR Ment Health*. 2020;7(2):e15914. doi:10.2196/15914, pmid:32027313

Our sample size in Phase 3 (one-on-one consultation with end users) was in the lower range of the average numbers for this type of study (between 6 and 12 participants) [89].

However, our sample size still enabled us to collect sufficient information for analysis in the framework and reach a saturation point.

Ospina-Pinillos L, Davenport TA, Ricci CS, Milton AC, Scott EM, Hickie IB. Developing a Mental Health eClinic to Improve Access to and Quality of Mental Health Care for Young People: Using Participatory Design as Research Methodologies. *J Med Internet Res*. 2018;20(5):e188. doi:10.2196/jmir.9716, pmid:29807878

Lastly, we used a purposeful sampling methodology in order to capture a diverse set of experiences in a time-efficient way, which is an essential part of the rapid iteration process at the heart of human-centered design. However, this approach yields a relatively small sample size. For example, this study only included one peer and one parent/guardian representative of WAP clients. While this is not uncommon in human-centered design, we acknowledge that this small number of users may limit the **generalizability of those findings to other communities and settings.**

Patel D, Sarlati S, Martin-Tuite P, et al. Designing an Information and Communications Technology Tool With and for Victims of Violence and Their Case Managers in San Francisco: Human-Centered Design Study. *JMIR Mhealth Uhealth*. 2020;8(8):e15866. doi:10.2196/15866, pmid:32831179

There are some limitations to this study. Only 14 users tested the app, and results cannot be generalized to all individuals with newly diagnosed type 2 diabetes.

Petersen M, Hempler NF. Development and testing of a mobile application to support diabetes self-management for people with newly diagnosed type 2 diabetes: a design thinking case study. *BMC Med Inform Decis Mak*. 2017;17(1):91. doi:10.1186/s12911-017-0493-6, pmid:28651639

First, having a small number of members from a single research group participate in our evaluation was a major limitation and may have introduced bias, given the likelihood of shared perspectives. Second, our decision to build APEEE using the Elastic Stack exposes the platform to open-source updates made by the community of Elastic developers.

Pham Q, Graham G, Lalloo C, et al. An Analytics Platform to Evaluate Effective Engagement With Pediatric Mobile Health Apps: Design, Development, and Formative Evaluation. *JMIR Mhealth Uhealth*. 2018;6(12):e11447. doi:10.2196/11447, pmid:30578179

An additional challenge in recruitment was that the PPI consultation meeting contained a small sample size, potentially leading to insufficient data and feedback.

Rai HK, Schneider J, Orrell M. An Individual Cognitive Stimulation Therapy App for People With Dementia: Development and Usability Study of Thinkability. *JMIR Aging*. 2020;3(2):e17105. doi:10.2196/17105, pmid:33196451

While the views of the 17 participants were coherent and data saturation was achieved, not all groups of potential users were similarly represented and it is possible that a more extensive engagement of potential users would lead to further improvements in acceptability and usability.

Rodrigues AM, Sniehotta FF, Birch-Machin MA, Olivier P, Araújo-Soares V. Systematic and Iterative Development of a Smartphone App to Promote Sun-Protection Among Holidaymakers: Design of a Prototype and Results of Usability and Acceptability Testing. *JMIR Res Protoc*. 2017;6(6):e112. doi:10.2196/resprot.7172, pmid:28606892

One noteworthy limitation was our small sample of stakeholders for the pilot that was mostly female and survivors of leukemia or lymphoma, thus calling into question the gen- eralizability of the feedback.

Schwartz LA, Psihogios AM, Henry-Moss D, et al. Iterative development of a tailored mHealth intervention for adolescent and young adult survivors of childhood cancer. *Clin Pract Pediatr Psychol*. 2019;7(1):31-43. http://doi.apa.org/getdoi.cfm?doi=10.1037/cpp0000272

A limitation of the study is that the application was designed only for one persona (“employee”). Further development and usability tests for the other personas are still to be made.

Sedlmayr B, Schöffler J, Prokosch H-U, Sedlmayr M. User-centered design of a mobile medication management. *Inform Health Soc Care*. 2019;44(2):152-163. doi:10.1080/17538157.2018.1437042, pmid:29504838

our sample of carers was limited by size and representativeness.

Sin J, Henderson C, Woodham LA, Sesé Hernández A, Gillard S. A Multicomponent eHealth Intervention for Family Carers for People Affected by Psychosis: A Coproduced Design and Build Study. *J Med Internet Res*. 2019;21(8):e14374. doi:10.2196/14374, pmid:31389333

Secondly, the reduced number of participants in the UCD process might be seen as an impediment of the applicability of the findings in a larger scale.

Smaradottir B, Holen-Rabbersvik E, Thygesen E, Fensli R, Martinez S. User-centred design of the user interface of a collaborative information system for inter-municipal dementia team. In: *Proceedings of the International Conference on Health Informatics*. SCITEPRESS - Science and and Technology Publications; 2015. http://www.scitepress.org/DigitalLibrary/Link.aspx?doi=10.5220/0005222704460453

This study presents some limitations that need to be considered. First, the limited number of male and younger patients (mean age 51 years) might limit the representativeness of the study.

Ledel Solem IK, Varsi C, Eide H, et al. A User-Centered Approach to an Evidence-Based Electronic Health Pain Management Intervention for People With Chronic Pain: Design and Development of EPIO. *J Med Internet Res*. 2020;22(1):e15889. doi:10.2196/15889, pmid:31961331

As our research was qualitative in nature, we have engaged a relatively small number of participants.

Stawarz K, Preist C, Tallon D, et al. Design Considerations for the Integrated Delivery of Cognitive Behavioral Therapy for Depression: User-Centered Design Study. *JMIR Ment Health*. 2020;7(9):e15972. doi:10.2196/15972, pmid:32880580

The limitations of this study are that only eight active midwives took part in a single region of Sweden and additional opinions may have been obtained if there had been a larger number of participants.

Stevenson JE, Oscarsson M. User-centred iterative design to develop an evidence-based communication application for maternity care. *Health Informatics J*. 2021;27(2):14604582211014579. doi:10.1177/14604582211014579, pmid:34030521

There was a sample size of 6 participants based on the fact that the majority of usability issues can be detected with 5 participants [16].

Uddin AA, Morita PP, Tallevi K, et al. Development of a Wearable Cardiac Monitoring System for Behavioral Neurocardiac Training: A Usability Study. *JMIR Mhealth Uhealth*. 2016;4(2):e45. doi:10.2196/mhealth.5288, pmid:27106171

Usability testing included 11 users across two countries; this was an adequate number given five users are considered optimal for usability testing (Nielsen, 2000), **although more users means greater confidence** (Faulkner, 2003). At the same time the number of users in the current study may not have been enough given the recommendation that mHealth technology design need to consider a range of variations in physical ability and MS symptoms (Simblett et al., 2019). **Nielsen also highlights that the more difficulties users have, the more participants you need** (Nielsen, 2000).

van Kessel K, Babbage DR, Kersten P, et al. Design considerations for a multiple sclerosis fatigue mobile app MS Energize: A pragmatic iterative approach using usability testing and resonance checks. *Internet Interv*. 2021;24:100371. doi:10.1016/j.invent.2021.100371, pmid:33614414

This study had several limitations. First, the number of patients with SMI in the expert panel group (n=2) and the usability testing study (n=5) could have been small, leaving to question whether a larger sample of people with SMI could have led to more feedback and opportunities for innovation. **There is debate among user-centered design researchers about the most cost-efficient number of subjects to identify usability errors [101,102], with the most traditional approach suggesting a sample size of 5 [101].**

Vilardaga R, Rizo J, Zeng E, et al. User-Centered Design of Learn to Quit, a Smoking Cessation Smartphone App for People With Serious Mental Illness. *JMIR Serious Games*. 2018;6(1):e2. doi:10.2196/games.8881, pmid:29339346

This study has some limitations. In view of the modest sam- ple size (especially in the direct observation and the needs part)

Willard S, Cremers G, Man YP, van Rossum E, Spreeuwenberg M, de Witte L. Development and testing of an online community care platform for frail older adults in the Netherlands: a user-centred design. *BMC Geriatr*. 2018;18(1):87. doi:10.1186/s12877-018-0774-7, pmid:29625562

Our preliminary results, although based on a very limited sample size, highlighted the potential benefits, as well as the drawbacks, of using the app to guide resuscitation.

Corazza F, Snijders D, Arpone M, et al. Development and Usability of a Novel Interactive Tablet App (PediAppRREST) to Support the Management of Pediatric Cardiac Arrest: Pilot High-Fidelity Simulation-Based Study. *JMIR Mhealth Uhealth*. 2020;8(10):e19070. doi:10.2196/19070, pmid:32788142

Quality of Subjects

Keywords: cultural differences (in users), small number (of users), super users vs users (test sampling), diversity of subjects,

Additionally, this was a relatively short session with only nine individuals total, seven of which were patients. The sample was overwhelmingly white and at least partially college educated (n ¼7), and **thus does not necessarily represent viewpoints of patients with different cultural or educational backgrounds**, the latter of which may be particularly relevant with regard to interpreting data.

Ahmed R, Toscos T, Rohani Ghahari R, et al. Visualization of Cardiac Implantable Electronic Device Data for Older Adults Using Participatory Design. *Appl Clin Inform*. 2019;10(4):707-718. doi:10.1055/s-0039-1695794, pmid:31533172

Despite collaborations with patient associations, we were able to mobilize only one CF family through all of our events. To address this gap, patients with CF have been recruited by our partner hospital for an upcoming event in France.

Balli F. Developing Digital Games to Address Airway Clearance Therapy in Children With Cystic Fibrosis: Participatory Design Process. *JMIR Serious Games*. 2018;6(4):e18. doi:10.2196/games.8964, pmid:30463835

The limited interactions experienced with study participants may also have missed any potential **“super-users”** – a category of system users that know more or are more technologically savvy than the average target user.

Carr EC, Babione JN, Marshall D. Translating research into practice through user-centered design: An application for osteoarthritis healthcare planning. *Int J Med Inform*. 2017;104:31-37. doi:10.1016/j.ijmedinf.2017.05.007, pmid:28599814

P2P was fortunate to obtain input from patients sampled from a pool of current patients; volunteer advisors who were willing to meet repeatedly with the design team; and various clinicians, some of whom also offered access to their clinic and protocols. Not every design team can easily access stakeholders for formative research in a timely manner, far less multiple stakeholder groups, especially when the stakeholders include busy professionals. **Some researchers resort to gathering data from less representative**

convenience samples, including online services offering access to paid volunteers, such as Amazon's Mechanical Turk or the Qualtrics Panel [73-75].

Patient advisors were more likely to be educated and engaged in their health than peers, consistent with the general trend that **patient advisors are rarely ordinary people.**

Cornet VP, Toscos T, Bolchini D, et al. Untold Stories in User-Centered Design of Mobile Health: Practical Challenges and Strategies Learned From the Design and Evaluation of an App for Older Adults With Heart Failure. *JMIR Mhealth Uhealth*. 2020;8(7):e17703. doi:10.2196/17703, pmid:32706745

The findings of our study reinforce the importance of considering the unique users of the technology we are proposing.

Davies A, Mueller J, Hennings J, Caress A-L, Jay C. Recommendations for Developing Support Tools With People Suffering From Chronic Obstructive Pulmonary Disease: Co-Design and Pilot Testing of a Mobile Health Prototype. *JMIR Hum Factors*. 2020;7(2):e16289. doi:10.2196/16289, pmid:32410730

The patient participants were from local CKD and general nephrology clinics, and the majority were older (aged >65 years) with less severe CKD. The website appears to be useful and accessible to older adults; however, the participants do not represent younger adults with CKD.

Donald M, Beanlands H, Straus SE, et al. A Web-Based Self-Management Support Prototype for Adults With Chronic Kidney Disease (My Kidneys My Health): Co-Design and Usability Testing. *JMIR Form Res*. 2021;5(2):e22220. doi:10.2196/22220, pmid:33560245

Other stakeholders involved in the home adaptation process, such as paying authorities, builders, and interdisciplinary health care team members [51], were not solicited. Broader perspectives of stakeholders might have enhanced the participatory design even more. Still, a design team comprising OTs, engineers, clinicians, and students provided ongoing input from the start of the study, as suggested by participatory design, and input from lay OTs, older adults, and their relatives was added during testing rounds, which is coherent with a user-centered process for the development of an eHealth technology [24].

Guay M, Labbé M, Séguin-Tremblay N, et al. Adapting a Person's Home in 3D Using a Mobile App (MapIt): Participatory Design Framework Investigating the App's Acceptability. *JMIR Rehabil Assist Technol*. 2021;8(2):e24669. doi:10.2196/24669, pmid:33973867

The main weakness of the study is the use of a convenience sample that was skewed toward older females aged over 65 years, with limited computer literacy. The sample was recruited through existing links and local carer groups and is, therefore, not representative of relatives supporting people with mental health problems or those likely to engage with Web-based interventions.

Honary M, Fisher NR, McNaney R, Lobban F. A Web-Based Intervention for Relatives of People Experiencing Psychosis or Bipolar Disorder: Design Study Using a User-Centered Approach. *JMIR Ment Health*. 2018;5(4):e11473. doi:10.2196/11473, pmid:30530457

First, all users and stakeholders participating in the study volunteered to participate on their own or were contacted by the project team or collaborating institutions. **As such, the participating groups are likely biased toward being more motivated, resourceful, and managing their life with chronic illness well. Thus, they may not perfectly represent the entire user group. However, this is too common for this kind of research**. Using other means for recruitment, such as social media [102], might have eased access to harder-to-reach users, but this was not within the project's mandate.

Jessen S, Mirkovic J, Nes LS. MyStrengths, a Strengths-Focused Mobile Health Tool: Participatory Design and Development. *JMIR Form Res*. 2020;4(7):e18049. doi:10.2196/18049, pmid:32706651

It appears that the co-design approach can be used with the involvement of a greater number of participants but, more importantly, **with a greater diversity of participants** to limit the risk of creating an overspecialized eHealth tool.

Latulippe K, Hamel C, Giroux D. Co-Design to Support the Development of Inclusive eHealth Tools for Caregivers of Functionally Dependent Older Persons: Social Justice Design. *J Med Internet Res*. 2020;22(11):e18399. doi:10.2196/18399, pmid:33164905

Our study has several limitations. Firstly, although a UCD with a purposive and diverse sample can be considered as a valuable design, we recruited volunteers, who maybe more interested in the tool and more experienced in health informatics. Therefore comments may have been more positive or more negative than if participants were randomly selected [56].

Marien S, Legrand D, Ramdoyal R, et al. A User-Centered design and usability testing of a web-based medication reconciliation application integrated in an eHealth network. *Int J Med Inform*. 2019;126:138-146. doi:10.1016/j.ijmedinf.2019.03.013, pmid:31029255

Additionally, the sample of participants was self-selected and some of the participants took part in earlier studies on personal strengths, which could have influenced participants' engagement and interest with the theme of the workshop and the methods that we used.

Mirkovic J, Jessen S, Kristjansdottir OB, Krogseth T, Koricho AT, Ruland CM. Developing Technology to Mobilize Personal Strengths in People with Chronic Illness: Positive Codesign Approach. *JMIR Form Res*. 2018;2(1):e10774. doi:10.2196/10774, pmid:30684404

A limitation of this study is that we included mainly male (former) patients with prostate cancer, and also the more empowered patients are probably overrepresented.

Noordman J, Driesenaar JA, van Bruinessen IR, van Dulmen S. ListeningTime; participatory development of a web-based preparatory communication tool for elderly cancer patients and their healthcare providers. *Internet Interv*. 2017;9:51-56. doi:10.1016/j.invent.2017.05.002, pmid:30135837

In examining multiple high-risk health behaviors, our participatory codesign samples included but did not focus on populations with characteristics of current smoking, high-risk drinking, or high-risk weight.

Ramsey AT, Bray M, Laker PA, et al. Participatory Design of a Personalized Genetic Risk Tool to Promote Behavioral Health. *Cancer Prev Res* . 2020;13(7):583-592. doi:10.1158/1940-6207.CAPR-20-0029, pmid:32209550

Furthermore, the sample that initially provided feedback (Stage II) consisted of more recent survivors, while the pilot (Stage III) included only long-term survi- vors, thus limiting input from survivors across the broad spectrum of early and later survivor- ship at each stage.

Schwartz LA, Psihogios AM, Henry-Moss D, et al. Iterative development of a tailored mHealth intervention for adolescent and young adult survivors of childhood cancer. *Clin Pract Pediatr Psychol*. 2019;7(1):31-43. http://doi.apa.org/getdoi.cfm?doi=10.1037/cpp0000272

our sample of carers was limited by size and representativeness.

Sin J, Henderson C, Woodham LA, Sesé Hernández A, Gillard S. A Multicomponent eHealth Intervention for Family Carers for People Affected by Psychosis: A Coproduced Design and Build Study. *J Med Internet Res*. 2019;21(8):e14374. doi:10.2196/14374, pmid:31389333

There were some limitations associated to this study, such as a reduced number of end-users and a simulated test environment.

Smaradottir B, Gerdes M, Martinez S, Fensli R. The EU-project United4Health: User-centred design of an information system for a Norwegian telemedicine service. *J Telemed Telecare*. 2016;22(7):422-429. doi:10.1177/1357633X15615048, pmid:26541347

The majority of our participants were women. This may be because women are more likely than men to seek mental health treatment [56] and the IAPT workforce is predominantly female [52].

Stawarz K, Preist C, Tallon D, et al. Design Considerations for the Integrated Delivery of Cognitive Behavioral Therapy for Depression: User-Centered Design Study. *JMIR Ment Health*. 2020;7(9):e15972. doi:10.2196/15972, pmid:32880580

Despite efforts to capture diverse experiences, the participant sample was relatively homogenous. Most participants identified as female, urban residents, educated, White, living with parents or guardians, and single.

Stoyanov SR, Zelenko O, Staneva A, et al. Development of the Niggle App for Supporting Young People on Their Dynamic Journey to Well-being: Co-design and Qualitative Research Study. *JMIR Mhealth Uhealth*. 2021;9(4):e21085. doi:10.2196/21085, pmid:33877050

The inclusion criterion for the pilot study was individuals aged 18 years and older; however, the ages of the volunteers all fell into the range of 18-45 years. This type of app may not be applicable for older people or those with poor literacy.

Tobias G, Spanier AB. Developing a Mobile App (iGAM) to Promote Gingival Health by Professional Monitoring of Dental Selfies: User-Centered Design Approach. *JMIR Mhealth Uhealth*. 2020;8(8):e19433. doi:10.2196/19433, pmid:32795985

The sample of individuals involved in the design of this decision aid focused on rural health and is representative of the dissemination area; however, a more racially/ethnically diverse sample will be needed to inform scalability.

Corazza F, Snijders D, Arpone M, et al. Development and Usability of a Novel Interactive Tablet App (PediAppRREST) to Support the Management of Pediatric Cardiac Arrest: Pilot High-Fidelity Simulation-Based Study. *JMIR Mhealth Uhealth*. 2020;8(10):e19070. doi:10.2196/19070, pmid:32788142

Like any study, this work has some limitations. First, the sample of older adults that participated in the design sessions had a slight overrepresentation of healthy older adults. As a result, the participants' views on assistive technology for people with cognitive decline may be too negative.

van Velsen L, van Weering MD-, Luub F, Neven A. Travelling with my SOULMATE: Participatory Design of an mHealth Travel Companion for Older Adults. In: *5th International Conference on Information and Communication Technologies for Ageing Well and E-Health*. unknown; 2019:38-47. Accessed February 8, 2022. https://www.researchgate.net/publication/333416006_Travelling_with_my_SOULMATE_Participatory_Design_of_an_mHealth_Travel_Companion_for_Older_Adults

Condensed Time Frame

Keywords: managing innovation equilibrium, duration, not blue sky (clash), lack of (clinician involvement, burdensome (elderly and ailing participants),

PD is generally conducted as an **iterative process involving multiple sessions situated to address specific and sequential design goals. The PD session presented in this article belongs to a more comprehensive iterative design study and thereby introduces the limitation of lacking full context from conception to validation.**

Ahmed R, Toscos T, Rohani Ghahari R, et al. Visualization of Cardiac Implantable Electronic Device Data for Older Adults Using Participatory Design. *Appl Clin Inform*. 2019;10(4):707-718. doi:10.1055/s-0039-1695794, pmid:31533172

Despite the co-design approach used in this study, there were still usability issues that **emerged related to the clinician portion of this application**. Further research on how this application can be better integrated into day-to-day practice is key to address the needs

of all stakeholders involved [11]. **These usability issues may have arisen owing to the nature of the research study, which was performed in addition to all regular day-to-day. Full integration of the application would require senior leadership support and a change in practice in order to be truly embedded into practice.**

Backman C, Harley A, Kuziemsky C, Mercer J, Peyton L. MyPath to Home Web-Based Application for the Geriatric Rehabilitation Program at Bruyère Continuing Care: User-Centered Design and Feasibility Testing Study. *JMIR Form Res*. 2020;4(9):e18169. doi:10.2196/18169, pmid:32924953

We identified 3 challenges related to the design phase of UCD: overcoming designers' assumptions with empirical research findings (design challenge 1), **managing project scope and complexity and avoiding scope creep** (design challenge 2), and **maintaining the innovation equilibrium** by balancing new ideas with outside constraints (design challenge 3).

Cornet VP, Toscos T, Bolchini D, et al. Untold Stories in User-Centered Design of Mobile Health: Practical Challenges and Strategies Learned From the Design and Evaluation of an App for Older Adults With Heart Failure. *JMIR Mhealth Uhealth*. 2020;8(7):e17703. doi:10.2196/17703, pmid:32706745

During the sessions, participants kept interacting with the game even when it was not completely ready in its final version. If participants had only interacted with the final version of the game, the results might have been different.

Da Silva Júnior JLA, Biduski D, Bellei EA, et al. A Bowling Exergame to Improve Functional Capacity in Older Adults: Co-Design, Development, and Testing to Compare the Progress of Playing Alone Versus Playing With Peers. *JMIR Serious Games*. 2021;9(1):e23423. doi:10.2196/23423, pmid:33512319

Overall limitations of many mHealth applications thus far include limited generalisability of study samples, **short study duration** (median time=18 months) and small sample sizes (median n=28).

Erguera XA, Johnson MO, Neilands TB, et al. WYZ: a pilot study protocol for designing and developing a mobile health application for engagement in HIV care and medication adherence in youth and young adults living with HIV. *BMJ Open*. 2019;9(5):e030473. doi:10.1136/bmjopen-2019-030473, pmid:31061063

A possible explanation is that after the first two weeks of use, patients started to change their treatments to optimize the health outcomes.

Stratification of patients according to their IT literacy and performance on communications, access and use objective metrics could lead to a better performance in terms of personalized medicine

We consider that a longer duration of the study would not have yielded any different findings in the observed metrics, and the differences on the use and communication values would continue as the trends observed in week 4.

Fico G, Martinez-Millana A, Leuteritz J-P, et al. User Centered Design to Improve Information Exchange in Diabetes Care Through eHealth : Results from a Small Scale Exploratory Study. *J Med Syst*. 2019;44(1):2. doi:10.1007/s10916-019-1472-5, pmid:31741069

Further investigation needs to be done correlating the technical results with the clinical and usability outcomes for longer periods of usage.

Fleisher L, Bass SB, Shwarz M, et al. Using theory and user-centered design in digital health: The development of the mychoice communication tool to prepare patients and improve informed decision making regarding clinical trial participation. *Psychooncology*. 2020;29(1):114-122. doi:10.1002/pon.5254, pmid:31654442

It was clear from this study that we were able to get a diverse range of stakeholders involved which included a variety of healthcare professionals, different types of patients, carers/relatives and researchers. Whilst in theory, this promoted a collaborative approach and formally embedded review points (in line with Catwell and Sheikh's model) that encouraged constant reflection, **the translation of this model in practice was challenging.** Although it was clear that stakeholders were fully engaged in the process, **it was often difficult to bring them together due to clinical commitments of the healthcare professionals and the fluctuating health of the patients involved.**

Giles SJ, Reynolds C, Heyhoe J, Armitage G. Developing a patient-led electronic feedback system for quality and safety within Renal PatientView. *J Ren Care*. 2017;43(1):37-49. doi:10.1111/jorc.12186, pmid:27990782

However, this method has a set of challenges. Indeed, the co-designers had to agree to build the tool based on previous decisions that were not their own. The research team had to take about 30 min at each CoD to explain the progress of the project and the decisions made by the previous groups. In addition, this method did not allow the co-designers to develop their co-creative skills over the long term and limits the possibility of developing a long-term relationship of trust.

The second was when the time allocated for the project ran out. At that point, some issues had to be left out of CoDs. The lack of time prompted the team to discern what was essential to designing an inclusive tool versus decisions that did not have a democratic stake.

Latulippe K, Hamel C, Giroux D. Co-Design to Support the Development of Inclusive eHealth Tools for Caregivers of Functionally Dependent Older Persons: Social Justice Design. *J Med Internet Res*. 2020;22(11):e18399. doi:10.2196/18399, pmid:33164905

Thirdly, in phase 3, due to time-constraints, a limited number of HCPs reconciled the medication list in absence of a participating patient.

Marien S, Legrand D, Ramdoyal R, et al. A User-Centered design and usability testing of a web-based medication reconciliation application integrated in an eHealth network. *Int J Med Inform*. 2019;126:138-146. doi:10.1016/j.ijmedinf.2019.03.013, pmid:31029255

Limitations of this work are inherent in the small sample size, and single site investigations in addition to the short time frame for exploitations.

Martin S, Armstrong E, Thomson E, et al. A qualitative study adopting a user-centered approach to design and validate a brain computer interface for cognitive rehabilitation for people with brain injury. *Assist Technol*. 2018;30(5):233-241. doi:10.1080/10400435.2017.1317675, pmid:28708963

Second, the TRP was tested for only a short period (2 weeks), and patients might have different experiences if followed over an extended period.

Naeemabadi M, Søndergaard JH, Klastrup A, et al. Development of an individualized asynchronous sensor-based telerehabilitation program for patients undergoing total knee replacement: Participatory design. *Health Informatics J*. 2020;26(4):2492-2511. doi:10.1177/1460458220909779, pmid:32175788

After a few sprints, it appeared that sprints of two weeks were not feasible and sprint duration was adapted to three weeks. Constantly involving elderly cancer patients and their HCPs is challenging. Not only because of time constraints (for all parties), but also the burden for patients to regularly participate when being seriously ill and/or elderly.

In addition, it was very di fficult to include oncological care providers, especially oncologists, because of their busy schedule.

Noordman J, Driesenaar JA, van Bruinessen IR, van Dulmen S. ListeningTime; participatory development of a web-based preparatory communication tool for elderly cancer patients and their healthcare providers. *Internet Interv*. 2017;9:51-56. doi:10.1016/j.invent.2017.05.002, pmid:30135837

For instance, the lack of time caused the evaluation of prototype version 1.0 in sprint 2 to be less in depth.

Rai HK, Schneider J, Orrell M. An Individual Cognitive Stimulation Therapy App for People With Dementia: Development and Usability Study of Thinkability. *JMIR Aging*. 2020;3(2):e17105. doi:10.2196/17105, pmid:33196451

In fact, we did not collect data on demographic or substance use in these samples for two key reasons: (i) the unique time con- straints of convenience sampling on busy pedestrian thorough- fares, which demanded expediency among uncompensated participants,

Ramsey AT, Bray M, Laker PA, et al. Participatory Design of a Personalized Genetic Risk Tool to Promote Behavioral Health. *Cancer Prev Res* . 2020;13(7):583-592. doi:10.1158/1940-6207.CAPR-20-0029, pmid:32209550

The time-consuming development was partly because of the inclusion of all the development phases but was also related to developing an mHealth intervention from a scientific environment. Developing an intervention from science involves completing an empirical cycle at each development phase and often includes an

extensive review of a study protocol by a medical ethics research committee. **Pursuing the rules of science during the development process of an mHealth intervention has slowed down the process at certain points in time.** Furthermore, development from a scientific environment generally means **less focus on business modeling and entrepreneurship, which could delay the process of bringing the app to the market.** Finally, a limitation in this study was the restricted budget available for the creative design and development of the mHealth intervention, which required us to make a selection in the development of intervention components.

Korpershoek YJG, Hermsen S, Schoonhoven L. User-centered design of a mobile health intervention to enhance exacerbation-related self-management in patients with chronic obstructive pulmonary …. *Journal of medical*. Published online 2020. https://www.jmir.org/2020/6/e15449/

Comprehension

Keywords: comprehension, linking experiences and objectives,

One such challenge is ensuring that the new IC tool improves patient **comprehension**. This study focused on testing the feasibility of developing and evaluating such a tool.

Abujarad F, Alfano S, Bright TJ, et al. Building an Informed Consent Tool Starting with the Patient: The Patient-Centered Virtual Multimedia Interactive Informed Consent (VIC). *AMIA Annu Symp Proc*. 2017;2017:374-383. https://www.ncbi.nlm.nih.gov/pubmed/29854101

Although the majority of co-designers contributed to the development of the prototype, 2 caregivers were unable to participate in the design activity. Despite the efforts of the research team members to clarify the objectives of the working session, these **2 individuals did not seem to be able to make the link between their experiences and the objectives of the session.**

Latulippe K, Hamel C, Giroux D. Co-Design to Support the Development of Inclusive eHealth Tools for Caregivers of Functionally Dependent Older Persons: Social Justice Design. *J Med Internet Res*. 2020;22(11):e18399. doi:10.2196/18399, pmid:33164905

Technology

Keywords: infrastructure, tools

Finally, we did not examine patients' access to **different personal technology tools** (eg, iPhone or Android mobile phones, tablets, or laptops).

Alberts NM, Badawy SM, Hodges J, et al. Development of the InCharge Health Mobile App to Improve Adherence to Hydroxyurea in Patients With Sickle Cell Disease: User-Centered Design Approach. *JMIR Mhealth Uhealth*. 2020;8(5):e14884. doi:10.2196/14884, pmid:32383683

In addition, participant selection was **biased** as all participants have regular access to high-speed internet. However, research indicates a significant proportion of the SCI community in North America (between 30% and 40%) do not have this kind of regular access, and almost 20% have never referred to the internet for health information

Allin S, Shepherd J, Tomasone J, et al. Participatory Design of an Online Self-Management Tool for Users With Spinal Cord Injury: Qualitative Study. *JMIR Rehabil Assist Technol*. 2018;5(1):e6. doi:10.2196/rehab.8158, pmid:29563075

The convenience sampling also relied on recruiting patients who used the in-house developed Diabetes Diary app and were therefore already engaged in mHealth for diabetes. The relevance of convenience sampling for mHealth studies is to recruit those who have experience and therefore experience-based suggestions for how to address the call for mHealth integration into clinical

Bradway M, Morris RL, Giordanengo A, Årsand E. How mHealth can facilitate collaboration in diabetes care: qualitative analysis of co-design workshops. *BMC Health Serv Res*. 2020;20(1):1104. doi:10.1186/s12913-020-05955-3, pmid:33256732

We found that many of the issues affecting our work with participants were similar to those faced by O'Connor et al [25], who attempted to co-design an app with dementia patients and their caregivers, including unfamiliarity with technology and incorrect perceptions about how users would interact with the technology.

So far, very little qualitative research regarding the perception of patients and their caregivers and how they experience technology has been carried out [26].

Besides the physical impairments, our study highlights the importance of considering lack of familiarity and confidence in technology use

Davies A, Mueller J, Hennings J, Caress A-L, Jay C. Recommendations for Developing Support Tools With People Suffering From Chronic Obstructive Pulmonary Disease: Co-Design and Pilot Testing of a Mobile Health Prototype. *JMIR Hum Factors*. 2020;7(2):e16289. doi:10.2196/16289, pmid:32410730

Another reason is nurses' attitudes towards technology and lack of perceived usefulness [7, 26]. In the CANA design process, we observed that nurses, especially older ones, are often cautious about using new technology. We often heard comments such as "I don't know how to use this phone", "do they charge me if I open the application in my mobile?", or "what happens if I make a mistake?"

By making the user interface simple (e.g., only 4 main categories) and friendlier (e.g., adding appropriate pictures for icons), we aimed to overcome some uncertainties about the application acceptance.

Dirin M, Dirin A, Laine TH. User-centered design of a context-aware nurse assistant (CANA) at Finnish elderly houses. In: *Proceedings of the 9th International Conference on Ubiquitous Information Management and Communication*. IMCOM '15. Association for Computing Machinery; 2015:1-8. Accessed August 17, 2022. https://doi.org/10.1145/2701126.2701225

As supported by related research work (Giles et al. 2013), this study demonstrates that patients are willing and able to feedback on a number of quality and safety issues relevant to their care. However, a number of barriers were evident, particularly around computer access and literacy and concerns about the consequences of identifying patient safety issues. Including the option to provide positive feedback was thought to mitigate some of these concerns to some extent. These barriers could be a reflection of the general concern about sharing electronic health data (Perera et al. 2011).

Giles SJ, Reynolds C, Heyhoe J, Armitage G. Developing a patient-led electronic feedback system for quality and safety within Renal PatientView. *J Ren Care*. 2017;43(1):37-49. doi:10.1111/jorc.12186, pmid:27990782

Local infrastructural limitations, particularly the lack of online functionality, **limited design decisions** that might harness the full range of benefits of mobile health interventions for this population, such as "on-the- go" use to facilitate practice (40, 41) or the ability to connect with other users (12, 31).

Gonsalves PP, Hodgson ES, Kumar A, et al. Design and Development of the "POD Adventures" Smartphone Game: A Blended Problem-Solving Intervention for Adolescent Mental Health in India. *Front Public Health*. 2019;7:238. doi:10.3389/fpubh.2019.00238, pmid:31508404

Gaps in the Literature

Pragmatic Hybridised Framework

Keywords: nuance, hybridised, pragmatic, mutuality, (current) breakdown, constraints (health care, digital), cross-staffing, method adaptation, infancy, digital vs health, attrition rates, broader ecosystem, minimising risk, throughout cycles, longitudinal iterative methodology, vast variance (design, metrics, outcomes), Interdisciplinary consensual coleadership, necessarily mixed-methods (and why), agile (by necessity), combining/tailoring methodologies, psychological wellbeing, human potential, co-designers or co-researchers?, contextual adaptation, whole-of-system

Value of Mixed-Method Approaches

Further research with both quantitative and qualitative design should be conducted to substantiate the findings of the study with diverse populations.

Mak WWS, Ng SM, Leung FHT. A Web-Based Stratified Stepped Care Platform for Mental Well-being (TourHeart+): User-Centered Research and Design. *JMIR Form Res*. 2023;7. https://www.scopus.com/inward/record.uri?eid=2-s2.0-85151375096&doi=10.2196%2f38504&partnerID=40&md5=4e50099456169bcbcf57a54414ad32d5

Another strength of this study is the use of quantitative methods for personas-construction. This allowed for a systematic personas- construction approach, such as identifying distinct groups of users through clustering followed by examining differences between personas post clustering. As a result, we were able to detect important differences in tool use between users that helped inform requirements for the design of the optimized tool. However, a limitation of employing a quantitative approach to personas-construction, is that our personas could not pro- vide insight into how these requirements can be operationalized in the tool, such as how to enhance the tool's adaptability across different usage contexts for an improved user experience. Future research may benefit from a mixed-methods approach to personas-construction including both quantitative and qualitative methods.

In this study, we matched the identified intrinsic user characteristics of personas with behavior change tech- niques using the taxonomy of behavior change methods [25,42] and translated these techniques into implementation strategies that could be integrated into a training module for the 'Valanalyse.'

Tyo MB, Desroches ML. Content analysis of qualitative interviews for user-centered design of a prototype mobile health app for direct support professionals' resilience. *Disabil Health J*. 2024;17(2). https://www.scopus.com/inward/record.uri?eid=2-s2.0-85177225810&doi=10.1016%2fj.dhjo.2023.101544&partnerID=40&md5=ad6621e38b05f3aef7ee0def2ddb4bef

In phase 1, the older adult end users tended to be very optimistic about how they would handle the system and the smartphone interface, overall giving higher scores in response to Likert statements and for the overall SUS score. Experts tended to be more pessimistic but this was probably due to their vast experience with older adults and technology. Most experts conceded that the use case analysis was a hypothetical one and that the capabilities of the older adult population are extremely variable, however, they felt that it was an extremely useful exercise in identifying major potential problems and addressing them early in the design process. Despite the difference in outlook between the experts and older adults, both groups reached agreement on most problems, particularly about the perceived difficulty of the login process and the lack of clear feedback when checking the system status and during the data upload process.

Harte R, Quinlan LR, Glynn L, et al. Human-Centered Design Study: Enhancing the Usability of a Mobile Phone App in an Integrated Falls Risk Detection System for Use by Older Adult Users. *JMIR Mhealth Uhealth*. 2017;5(5):e71. doi:10.2196/mhealth.7046, pmid:28559227

Another important insight from our work reflects on the validity of user feedback. Features that users appeared to struggle with according to observation notes were nevertheless described as fine by users, suggesting a social desirability bias [30]. This underscores the importance of developing apps not only based on users' verbalizations and responses to specific questions (such as standardized usability scales) but on observations of user behavior while engaging with the app.

Attrition rates are a known issue in digital health interventions [33]. The high level of attrition seen in our study is, therefore, not entirely unexpected. An understanding of why people discontinue use is important and worthy of further research given that retention is key for the management of chronic conditions over time [33].

The usage data indicate that the app was used as intended with most of the activity surrounding the recording and viewing of symptom data, which was the app's principal purpose. The low usage of the help feature may suggest that the app was also fairly intuitive for most people. The biggest issue surrounds the lack of continued use of the app over time. More research is needed to identify the intrinsic and extrinsic reasons behind the attrition in this context. **Longer-term analysis of app usage** before dropout using in the wild (deployed in the real world) event capture may help to shed light on some of these reasons.

The combination of qualitative data analysis and data collected from open-source event capture tools also served to offer a further insight into app usage and dropout.

Davies A, Mueller J, Hennings J, Caress A-L, Jay C. Recommendations for Developing Support Tools With People Suffering From Chronic Obstructive Pulmonary Disease: Co-Design and Pilot Testing of a Mobile Health Prototype. *JMIR Hum Factors*. 2020;7(2):e16289. doi:10.2196/16289, pmid:32410730

Results reject our initial hypothesis, as T2DM participants achieved a comparable level of use and communication metrics, and the differences between the first and the second half of the study on these indicators are smaller than the differences observed in the T1DM group.

Fico G, Martinez-Millana A, Leuteritz J-P, et al. User Centered Design to Improve Information Exchange in Diabetes Care Through eHealth : Results from a Small Scale Exploratory Study. *J Med Syst*. 2019;44(1):2. doi:10.1007/s10916-019-1472-5, pmid:31741069

In our research, we have tried to identify the benefits of user centered design in order to design, deliver and test a tailored solution for diabetes management. Furthermore, the integration of standard sensors for bio- signal acquisition is an essential element in order to implement a reliable information workflow, as

confirmed by [32]. The designed system fills the gap identified in a recent review on T2DM mobile apps [13]: from the 89 apps analysed, a majority was of high quality with respect to a single dimension of the disease but only 4 out of 89 apps integrated all six dimensions, and less than half integrated at least four of them.

Fico G, Martinez-Millana A, Leuteritz J-P, et al. User Centered Design to Improve Information Exchange in Diabetes Care Through eHealth : Results from a Small Scale Exploratory Study. *J Med Syst*. 2019;44(1):2. doi:10.1007/s10916-019-1472-5, pmid:31741069

Nevertheless, simply adding all functionalities requested by end-users without due consideration could bring severe usability issues and impair user experience. The SUS score in our second design iteration dropped significantly to 52.2 ± 11.0 from 63.75 ± 7.2 mainly due to these issues. For example, based on user requests, we added a number of tuto- rials hoping that they will help users quickly get a sense of the different functionalities of the system. However, in the usability evaluation focus group of the second design iteration, a number of participants ignored the newly added tutorials. When asked, they expressed that "I am confident that I can learn how to use the system myself." However, compared to other resources that users used to find relevant health information such as Google search engine and WebMD, a graph-based visualization tool is signifi- cantly different; and thus, it is not easy for users from the general public to get familiar with quickly.

He X, Zhang R, Rizvi R, et al. ALOHA: developing an interactive graph-based visualization for dietary supplement knowledge graph through user-centered design. *BMC Med Inform Decis Mak*. 2019;19(Suppl 4):150. doi:10.1186/s12911-019-0857-1, pmid:31391091

Similar to previous studies, our design and testing process found that **using a combination of methodologie**s (in our case several different types of qualitative inquiry) produced more robust results than a single user-centered approach alone [21,22]. For example, if we had relied on only patient one-on-one interviews, we would have missed key provider perspectives about how to focus in on their new patients for whom the tool could be particularly beneficial.

Second, we did not do more formal usability testing (such as with validated usability ratings) [18], relying instead on more general prototype iterations to improve the layout.

Lyles CR, Altschuler A, Chawla N, et al. User-Centered Design of a Tablet Waiting Room Tool for Complex Patients to Prioritize Discussion Topics for Primary Care Visits. *JMIR Mhealth Uhealth*. 2016;4(3):e108. doi:10.2196/mhealth.6187, pmid:27627965

The quantitative and qualitative data on usability and usefulness were somewhat contrasting but highly complementary. The scores derived from the 2 questionnaires showed that most patients were satisfied and that most believed the app could improve

the accuracy of medication lists and communication and reduce the time spent on reconciling medications. **Nonetheless, several scores slightly decreased between the beginning and end of the study**. It is possible that some patients were disappointed because of technical errors that occurred and because HCPs did not use the list generated by patients.

In the context of a future study, 63 it would be useful to evaluate how patient health, eHealth, computer literacy, and levels and patterns of use influence research results.

Fifth, it was not feasible to evaluate efficiency in a quantita- tive way. This should be done in the future, as a tool will not be adopted if a burdensome number of clicks or too much time is needed to achieve the desired goal.

Marien S, Legrand D, Ramdoyal R, et al. A web application to involve patients in the medication reconciliation process: a user-centered usability and usefulness study. *J Am Med Inform Assoc*. 2018;25(11):1488-1500. doi:10.1093/jamia/ocy107, pmid:30137331

Additionally, the need for both technical and therapeutic stakeholders in the development and design of new technology is essential to harness the new opportunities offered through these innovations. (How et al., 2015).

The evaluation of the system with a control group and with people living with TBI suggested the potential for BCI as a platform for a cognitive rehabilitation application, and despite the technical issues that emerged, all participants were able to complete each of the cognitive rehabilitation tasks in excess of the recommended 70% threshold for BCI accuracy (Nijboer et al., 2008).

Martin S, Armstrong E, Thomson E, et al. A qualitative study adopting a user-centered approach to design and validate a brain computer interface for cognitive rehabilitation for people with brain injury. *Assist Technol*. 2018;30(5):233-241. doi:10.1080/10400435.2017.1317675, pmid:28708963

A shift toward adopting data-driven research methods would mark a significant development for the field, which has historically been “data-rich but evidence-poor” [26,27]. We posit that the adaptation of pediatric mHealth apps at the right time and under the right circumstances can accelerate evaluative practice and improve health outcomes.

Pham Q, Graham G, Lalloo C, et al. An Analytics Platform to Evaluate Effective Engagement With Pediatric Mobile Health Apps: Design, Development, and Formative Evaluation. *JMIR Mhealth Uhealth*. 2018;6(12):e11447. doi:10.2196/11447, pmid:30578179

The evaluation of the SUS scores of the first and third prototype proved that the iterative process described above improved usability.

Schild S, Sedlmayr B, Schumacher A-K, et al. A Digital Cognitive Aid for Anesthesia to Support Intraoperative Crisis Management: Results of the User-Centered Design Process. *JMIR Mhealth Uhealth*. 2019;7(4):e13226. doi:10.2196/13226, pmid:31033445

However, our results are descriptive in nature and limited in establishing the usability or acceptability of the intervention,

Sin J, Henderson C, Woodham LA, Sesé Hernández A, Gillard S. A Multicomponent eHealth Intervention for Family Carers for People Affected by Psychosis: A Coproduced Design and Build Study. *J Med Internet Res*. 2019;21(8):e14374. doi:10.2196/14374, pmid:31389333

We have conducted comprehensive testing with input from both therapists and patients unlike many apps that are devel- oped for patients. **Testing was over 4–6 weeks of use allow ing users time to test it thoroughly.**

Another limitation of this study is that we had hoped to be able to monitor exercise adherence using user activation tracking system, but this would only be possible if patients continued to use the app.

Tonga E, Williamson E, Srikesavan C, Özen T, Sarıtaş F, Lamb SE. A hand exercise mobile app for people with rheumatoid arthritis in Turkey: design, development and usability study. *Rheumatol Int*. 2021;41(6):1151-1160. doi:10.1007/s00296-021-04860-0, pmid:33870452

Organic Co-Design

If health care organizations invite patients to co-design, health care professionals must share power with them and work responsibly to overcome barriers on equal terms [41]. On the basis of the results of this study, we cannot determine whether a remote co-design initiative with older persons supports patient safety.

Holmqvist M, Ros A, Lindenfalk B, Thor J, Johansson L. How Older Persons and Health Care Professionals Co-designed a Medication Plan Prototype Remotely to Promote Patient Safety: Case Study. JMIR Aging. 2023;6.
https://www.scopus.com/inward/record.uri?eid=2-s2.0-85159816805&doi=10.2196%2f41950&partnerID=40&md5=55360546f0c961180d1448530932d72b

User involvement in design and evaluation stages is common in the health domain, but **user contributions are often dismissed in the implementation process what results in systems facing user refusal since they are not aligned enough to the healthcare delivery or present a limited usability.**

There is an increasing understanding of the relevance of adopting user-centred design and development techniques in the health sector. **However, users are often assumed to be a homogeneous group with the same set of requirements.** In this work, the study case of an e-Health system for renal patients at home has been presented, where users are categorized in four different groups (i.e., digital patients/caregivers, non-digital patients/caregivers, clinicians and nurse). User requirements of each group have been analysed and different modules have been developed to coping with them, resulting in a multi-faceted e- Health system currently in piloting. Identification and continuous in-

volvement of users allow their needs to be properly understood and addressed by technology, raising user acceptance of the final product.

Curtis KE, Lahiri S, Brown KE. Targeting Parents for Childhood Weight Management: Development of a Theory-Driven and User-Centered Healthy Eating App. *JMIR Mhealth Uhealth*. 2015;3(2):e69. doi:10.2196/mhealth.3857, pmid:26088692

The different stages of evaluation are designed to maximize the usefulness of the users' involvement. Indeed, it is critical to make the best use of the care providers' time in the implementation process because including participants is often difficult due to limited time. In addition, performing evaluations of growing ecological validity allowed us to **minimize the risks associated with HIT in health care [50].**

By starting with a low ecological validity evaluation, our model helps resolve many issues at an early stage without the risk of compromising the integrity of data.

We were also particularly fortunate to have support from both top- and bottom-level stakeholders, who were involved at all stages. **We emphasize the importance of user involvement and feedback in iterative cycles throughout the process to help ensure that the design and development are as tailored to the needs and workflow as possible.**

Each step of this process is necessary to ensure the creation of a useful and effective app that can truly support user needs, within a given workflow process. Although a close collaboration with clinicians throughout the entire project facilitated the development of a tailored solution, it was also important to involve all stakeholders, in particular, the IT security officers.

Ehrler F, Lovis C, Blondon K. A Mobile Phone App for Bedside Nursing Care: Design and Development Using an Adapted Software Development Life Cycle Model. *JMIR Mhealth Uhealth*. 2019;7(4):e12551. doi:10.2196/12551, pmid:30973339

The research team's impartiality meant that they were able to facilitate an agreed way forward.

Giles SJ, Reynolds C, Heyhoe J, Armitage G. Developing a patient-led electronic feedback system for quality and safety within Renal PatientView. *J Ren Care*. 2017;43(1):37-49. doi:10.1111/jorc.12186, pmid:27990782

Designing an eHealth technology such as MapIt with potential users **and evaluating it in a clinical contex**t allowed to **consider its acceptability from the start,** as suggested by the user-centered design [24]. However, contrary to participatory design principles, the study did not truly start at the fuzzy front-end of a co-designing process [48], exploring in detail the unmet needs of people [31] involved in improving the health and well-being through home adaptations. Indeed, to determine what was to be designed or not, a participatory design process was steered toward the team members' a priori, relying on their past research, technical, and clinical experiences. More ambiguity at the start might have led to other different solutions, whether technological or not.

Interdisciplinary consensual coleadership has probably contributed to dealing efficiently with inherent tensions and encouraged open creative thinking, while focusing on getting to a clinical hands-on solution.

Guay M, Labbé M, Séguin-Tremblay N, et al. Adapting a Person's Home in 3D Using a Mobile App (MapIt): Participatory Design Framework Investigating the App's Acceptability. *JMIR Rehabil Assist Technol*. 2021;8(2):e24669. doi:10.2196/24669, pmid:33973867

The development of the app, using a codesign process, is a significant advance on many of the currently available apps (and other technology-based interventions) that have been designed to address mental ill health because of the incorporation of the youth perspective. **The inclusion of both clinicians and young people in a codesign process highlighted disparate needs, motivations, and intentions for the app, and by incorporating the views of both, the app has promise as a tool to assist both clinicians and young people in the management of depression and suicide-related behaviors.**

Hetrick SE, Robinson J, Burge E, et al. Youth Codesign of a Mobile Phone App to Facilitate Self-Monitoring and Management of Mood Symptoms in Young People With Major Depression, Suicidal Ideation, and Self-Harm. *JMIR Ment Health*. 2018;5(1):e9. doi:10.2196/mental.9041, pmid:29362208

In general, user inputs and concerns were maintained through 3 different strategies: (1) having a user representative, who is equal to everyone else in the project group (this person, it is important to highlight, works as a nurse and has a medical or nursing background, something that may also reduce the hierarchical distance between them and the researchers); (2) using the outcome from the co-design workshops and user evaluations in the discussions; and (3) having all project members and researchers do the same design tasks as the participants in the co-design workshop. This third strategy also supported the important task of communicating and creating a deeper understanding of the users' needs and requirements within the project group [99]. This allowed the group to see not only the outcomes of the participants' work but also to go through the process of creating similar types of work themselves.

When projects clear the proverbial fuzzy front end [54] and finally start building the actual product, the pace of the work tends to increase. In earlier phases of the project, recruiting participants to activities was often done in collaboration with the institutions participating in the project's activities. **By recruiting only for specific activities, we had to reach out again during later phases and re-recruit the same participants**. This made organizing the recruitment and activities for participation time consuming and thus more challenging than envisioned.**we would recommend creating a systematic plan for recruitment and participation for the entirety of the project.**

We had to abandon the implementation of social features, primarily because of restrictions from the privacy officers at our institution. Although we could have foreseen this issue and removed the possibility of social features in our activities, we might have

restricted the participants' creativity and range of possibilities. Furthermore, by working without restrictions during the workshops, participants also give important information and feedback that, for instance, are relevant not only to social features but also to mHealth tools as a whole. It may seem more efficient concerning time and money to keep participatory activities focused on what is possible or advisable to create. However, we still recommend allowing for free creativity and ideation in such activities, as this can yield not only interesting ideas and concepts but also valuable insights into the user group and their needs and wishes.

Jessen S, Mirkovic J, Nes LS. MyStrengths, a Strengths-Focused Mobile Health Tool: Participatory Design and Development. *JMIR Form Res*. 2020;4(7):e18049. doi:10.2196/18049, pmid:32706651

Previous work on serious games has experienced similar variation in the output and value of end user involvement throughout the design process [40]. A concept introduced by Khaled and Vasalou [41] suggests a finer approach to participant selection in co-design, differentiating contributions in terms of domain expertise and procedural aspects to overcome some of the limitations that hinder current contributions to the design process; however, this approach has not been extensively tested.

Focus groups and questionnaires were used for collecting design ideas and feedback on the usability and acceptability of the PEGASO F4F technology, and feedback guided the development of the apps and serious game. Although the use of focus groups and questionnaires reflects a solid approach to ascertain usability, additional methods need to be considered when refining the PEGASO F4F apps and serious game in future research.

Second, collection and interpretation of system usage data can provide valuable information in terms of measuring the level of engagement with mHealth technology [43]. A mixed method approach is recommended during mHealth technology development in which quantitative data (eg, back-end system data) reflect objective usage and qualitative data (eg, semistructured interview) provide more insight into reasons for playing or using, or more importantly, not playing the game or using apps [43]. This would give designers and researchers a better and a more concrete understanding on which elements to adapt, and how to translate adolescents feedback into engaging age-appropriate app and game design [5].

Martin A, Caon M, Adorni F, et al. A Mobile Phone Intervention to Improve Obesity-Related Health Behaviors of Adolescents Across Europe: Iterative Co-Design and Feasibility Study. *JMIR Mhealth Uhealth*. 2020;8(3):e14118. doi:10.2196/14118, pmid:32130179

Fifth, design thinking was the primary framework used to develop the tool, **which did not take into account other considerations such as technology acceptance, behavior change, or patient engagement models.**

Marko-Holguin M, Cordel SL, Van Voorhees BW, et al. A Two-Way Interactive Text Messaging Application for Low-Income Patients with Chronic Medical Conditions: Design-Thinking Development Approach. *JMIR Mhealth Uhealth*. 2019;7(5):e11833. doi:10.2196/11833, pmid:31042152

During this study, a new theme emerged, which added the missing piece of the methodology, **the contextual adaptation**. As a result, it was possible to obtain culturally and contextually appropriate information about what is required in terms of content and functionality, as well as preferences for the prototype's interface and the technology platform. All of this was done in a participative, collaborative, and time-efficient manner. The approach enabled us to collect information, define the needs, and find solutions on how the MHeC-C would respond to these requirements.

Ospina-Pinillos L, Davenport TA, Navarro-Mancilla AA, et al. Involving End Users in Adapting a Spanish Version of a Web-Based Mental Health Clinic for Young People in Colombia: Exploratory Study Using Participatory Design Methodologies. *JMIR Ment Health*. 2020;7(2):e15914. doi:10.2196/15914, pmid:32027313

Due to highly domain-specific content and medical terminology related to the laboratory tests, the developer team and HCI consultant had to go through a steep learning curve to familiarize themselves with the context of the mHealth app. The hierarchy, organizational structure, and bureaucracy of the hospital environment also made this process somewhat challenging for these external stakeholders. To this end, having a medical professional with experience in participatory and qualitative research as a facilitator significantly helped the external parties to overcome those challenges and understand the context quickly. Provision of an overview of the organizational structure, culture, and existing workflows, coordinating with internal stakeholders, co-conducting activities, and working around cultural barriers are some examples of the roles of the facilitator.

Saparamadu AADNS, Fernando P, Zeng P, et al. User-Centered Design Process of an mHealth App for Health Professionals: Case Study. *JMIR Mhealth Uhealth*. 2021;9(3):e18079. doi:10.2196/18079, pmid:33769297

(vi) Rather than asking questions about their life, **it can be more fruitful to have older adults per- form a creative activity, e.g., fill in a diary, take pictures, or draw something (a storyboard) and let them talk meanwhile;**

Third, **technical engineers did not cooperate in the participatory design process. In order to reach commitment among partners and shared understanding, it would have been better to include system engi- neering from the beginning in joint sessions with users, designers and researchers and have them witness user evaluation sessions.** One of the most difficult challenges was how to make sure initial insights were not lost somewhere during the pro- cess.

Verhoeven F, Cremers A, Schoone M, Van Dijk J. Mobiles for mobility: Participatory design of a “Happy walker” that stimulates mobility among older people. *Gerontechnology*. 2016;15(1). https://journal.gerontechnology.org/currentIssueContent.aspx?aid=2248

Fluid Digital+Health Efficacy

Once fully developed and implemented, studies should be designed to **understand the impact of such designed information system compared to other systems**, as well as standard care alone. These may include patients' and carers' hospital experience satisfaction, changes in anxiety and postoperative maladaptive behaviour, or healthcare outcomes including recovery rate indicated by e.g. the length of hospital stay. **They should also explore how the system could be used to connect patients and parents, and how to actively involve healthcare professionals during the that process.**

Aufegger L, Bùi KH, Bicknell C, Darzi A. Designing a paediatric hospital information tool with children, parents, and healthcare staff: a UX study. *BMC Pediatr*. 2020;20(1):469. doi:10.1186/s12887-020-02361-w, pmid:33032549

Our study **was not designed to evaluate the impact of the application on health outcomes or care processes**.

Backman C, Harley A, Kuziemsky C, Mercer J, Peyton L. MyPath to Home Web-Based Application for the Geriatric Rehabilitation Program at Bruyère Continuing Care: User-Centered Design and Feasibility Testing Study. *JMIR Form Res*. 2020;4(9):e18169. doi:10.2196/18169, pmid:32924953

To benefit both of these end-user groups, the system should structure the data in a relevant and usable way, and be flexible enough to present different levels of in- formation, i.e. summarized and in-depth, and be under- standable for both patients and providers in order to. To benefit both of these end-user groups, the system should structure the data in a relevant and usable way, and be flexible enough to present different levels of in-formation, i.e. summarized and in-depth, and be understandable for both patients and providers in order to

Bradway M, Morris RL, Giordanengo A, Årsand E. How mHealth can facilitate collaboration in diabetes care: qualitative analysis of co-design workshops. *BMC Health Serv Res*. 2020;20(1):1104. doi:10.1186/s12913-020-05955-3, pmid:33256732

While our goal of soliciting input from different types of end-users uncovered many themes that were feasible to address with a mobile app, there are additional limitations to consider. **App features addressed intrinsic factors such as motivation and confidence, but not health system constraints, which were frequently cited as barriers.**

Chan NH-M, Merali HS, Mistry N, et al. Development of a novel mobile application, HBB Prompt, with human factors and user-centred design for Helping Babies Breathe skills retention in Uganda. *BMC Med Inform Decis Mak*. 2021;21(1):39. doi:10.1186/s12911-021-01406-z, pmid:33541340

Strategies to overcome these collaboration challenges include structured communication and the involvement of a multilinguistic (symphonic) conductor, a person who has learned “each team member’s discipline- or profession-specific values, norms, practices, vocabularies, theories, and methods to coordinate and translate between dissimilar members”

We attempted to promote direct interactions between designers and both patient and clinician stakeholders. Designers attended many of the formative research sessions or had direct access to the collected raw data. Furthermore, to ensure continuity, there was **cross-staffing** of formative research, design, and evaluation teams.

In the context of mHealth projects, **adapting standardized usability evaluation methods** to end users is often necessary to accommodate patient abilities and limitations. For example, most standardized usability scales have technical or difficult words [86]; thus, many studies edit these measures, for example, by changing the “cumbersome” in the SUS to “awkward” [88].

Cornet VP, Toscos T, Bolchini D, et al. Untold Stories in User-Centered Design of Mobile Health: Practical Challenges and Strategies Learned From the Design and Evaluation of an App for Older Adults With Heart Failure. *JMIR Mhealth Uhealth*. 2020;8(7):e17703. doi:10.2196/17703, pmid:32706745

Based on our findings, we recommend that participatory user-centered design of mHealth apps for patients and care partners in the hospital setting particularly **emphasize four heuristics: match between system and the real world, con-sistency and standards, flexibility and efficiency of use, and aesthetic and minimalist design.** We found the process of addressing these four heuristics with end-users improved design aspects aligned with all of Nielsen’s heuristics, and this should be confirmed with future work.

Couture B, Lilley E, Chang F, et al. Applying User-Centered Design Methods to the Development of an mHealth Application for Use in the Hospital Setting by Patients and Care Partners. *Appl Clin Inform*. 2018;9(2):302-312. doi:10.1055/s-0038-1645888, pmid:29742756

However, similar to other psychological models and health behavior change intervention frameworks, the BCW stops short of serving as a guide when it comes to translating behavior change techniques into mHealth app features due to the infancy of the mHealth field. Additionally, it’s execution relied heavily on the expertise, creativity, and judicious decision making of the design team with regards to which components should actually be implemented in the app, as well as drawing on existing evidence, practical considerations, end-users’ views, and expert advice. **Thus, it is necessary to expand on the BCW using other disciplines in design and engineering and**

collaborate with the commercial app industry for the development of behavior change interventions that is relevant for the mobile app ecosystem

Since conducting this study, a new framework, Behavioral Intervention Technology (BIT) model, has been published which attempts to integrate both conceptual and technological components of electronic health (eHealth) and mHealth interventions [60]. In particular, it offers a method for targeting distal clinical aims (eg, weight reduction) and translating behavior change strategies into an app features.

Curtis KE, Lahiri S, Brown KE. Targeting Parents for Childhood Weight Management: Development of a Theory-Driven and User-Centered Healthy Eating App. *JMIR Mhealth Uhealth*. 2015;3(2):e69. doi:10.2196/mhealth.3857, pmid:26088692

These investigations suggest technology-based interventions can improve ART medication management with nearly 65% of all mHealth studies published between 2009 and 2012 reporting positive adherence outcomes.19 **While these results are encouraging, comparisons across studies are difficult owing to differences in study design, intervention and outcome measurements,**19 and under- score the need for more standardised ways of evaluating mHealth interventions.14

In the evaluation of WYZ, **we propose to measure and report on application engagement using a combination of metrics and thresholds including feasibility** (table 2) and acceptability metrics, as well as a novel EI used in prior mHealth application development.44

Erguera XA, Johnson MO, Neilands TB, et al. WYZ: a pilot study protocol for designing and developing a mobile health application for engagement in HIV care and medication adherence in youth and young adults living with HIV. *BMJ Open*. 2019;9(5):e030473. doi:10.1136/bmjopen-2019-030473, pmid:31061063

Further investigation needs to be done **correlating the technical results with the clinical and usability outcomes for longer periods of usage.**

Fleisher L, Bass SB, Shwarz M, et al. Using theory and user-centered design in digital health: The development of the mychoice communication tool to prepare patients and improve informed decision making regarding clinical trial participation. *Psychooncology*. 2020;29(1):114-122. doi:10.1002/pon.5254, pmid:31654442

Although our app conforms to clinical practice guideline recommendations with regard to routine monitoring of symptoms (depression) and medication side effects (suicidal ideation) and its prototype has been beta-tested [10], **there is a need to robustly test the app for efficacy and safety,** including testing that the innovative mood rating function is a reliable and valid measure of mood compared with validated measures such as the PHQ-9 and our 3-item suicide risk screener [10].

Hetrick SE, Robinson J, Burge E, et al. Youth Codesign of a Mobile Phone App to Facilitate Self-Monitoring and Management of Mood Symptoms in Young People With Major Depression, Suicida

In the next phase, the effect of the mHealth intervention on the relevant patient outcomes and health care use should be evaluated. Recent studies on mHealth interventions in patients with COPD suggest the use of randomized controlled trials (RCTs) with adequately powered sample sizes and a 1-year follow-up period to be sufficient to comment on behavioral change and impact of treatment [19,20].

However, this time-consuming design may not be ideal for rapidly evolving mHealth technologies [73,76]. Using an RCT implies two or more years of research in which this mHealth intervention with high potential for effectiveness, and no expected harm will not be available for patients with COPD.

However, this time-consuming design may not be ideal for rapidly evolving mHealth technologies [73,76]. Using an RCT implies two or more years of research in which this mHealth intervention with high potential for effectiveness, and no expected harm will not be available for patients with COPD. Within these designs, it is important to evaluate self-management skills and behavior change as outcomes, and the way this is assessed should be clearly reported [19,77,78].

Korpershoek YJG, Hermsen S, Schoonhoven L. User-centered design of a mobile health intervention to enhance exacerbation-related self-management in patients with chronic obstructive pulmonary .... *Journal of medical*. Published online 2020. https://www.jmir.org/2020/6/e15449/

We presented the 2 dominant schools of thought on this question. One posits that people can contribute meaningfully by expressing their interests, attitudes, beliefs, values, and expectations, by demonstrating their capacities [10,18], and by describing the context in which they will use the eHealth tool [25]. In addition, they can identify what is useful and relevant [21]. Their participation may also lead to innovation [24]. On the other hand, skeptics of the co-design approach timidly argue that people may not be aware of their needs, be able to express their needs, or want to discuss their needs in a group setting [28]; that the technology developed can be overspecialized and relevant only to a few [28]; and that people will have difficulty contributing significantly if they do not have technological skills [15]. Our position? Somewhere between these 2 paradigms, we advocate the use of co-design while keeping a critical eye.

let us take this reflection a step further and answer the following question: **Can co-design be considered a democratic process, as described by Sen?** Can an equal distribution of decision-making power be implemented between the different co-designers? In all humility, the answer to this question is more nuanced.

In this experiment, the role of the research team in co-designing and its neutrality in the research project were ambiguous. I**ts original intent was to remain neutral to leave the decision-making power entirely in the hands of the co-designers; however, its**

position evolved to where the team defined itself as a co-designer in its own right and, finally, to become a more important decision maker, all the while respecting the decisions made by the other co-designers. The underlying reflection varied over time and between researchers. It seemed impossible for the research team to not intervene directly in the development of the tool and therefore to make decisions without debate with the co-designers. What practical impact did this choice have on the democratic process, as understood by Sen?

Could it have been otherwise? **Theoretically, a genuine sharing of power requires participants to have an enabling role. From a pragmatic standpoint, is this one of the limits of co-design, and even an epistemological limit?**

Latulippe K, Hamel C, Giroux D. Co-Design to Support the Development of Inclusive eHealth Tools for Caregivers of Functionally Dependent Older Persons: Social Justice Design. *J Med Internet Res*. 2020;22(11):e18399. doi:10.2196/18399, pmid:33164905

Besides, working in close collaboration with the ReHN gave us the possibility to work with real data, coming from multiple sources– unlikely most eMedRec tools [5, 23, 53,62, 66, 73]– and in a real clinical setting to test the high-fidelity prototype.

Marien S, Legrand D, Ramdoyal R, et al. A User-Centered design and usability testing of a web-based medication reconciliation application integrated in an eHealth network. *Int J Med Inform*. 2019;126:138-146. doi:10.1016/j.ijmedinf.2019.03.013, pmid:31029255

Second, **we did not analyze data categorizing the participant texting actual content, response sentiment (eg, positive, negative, and neutral), response frequency (ie, how often a user responded), or response time.** Future research should examine these variables to better tailor the tool for end-users.

Fifth, design thinking was the primary framework used to develop the tool, **which did not take into account other considerations such as technology acceptance, behavior change, or patient engagement models.**

Marko-Holguin M, Cordel SL, Van Voorhees BW, et al. A Two-Way Interactive Text Messaging Application for Low-Income Patients with Chronic Medical Conditions: Design-Thinking Development Approach. *JMIR Mhealth Uhealth*. 2019;7(5):e11833. doi:10.2196/11833, pmid:31042152

Furthermore, we cannot establish what the outcome would have been if we had made different design decisions or applied a different theory of behavior change.

Martin S, Armstrong E, Thomson E, et al. A qualitative study adopting a user-centered approach to design and validate a brain computer interface for cognitive rehabilitation for people with brain injury. *Assist Technol*. 2018;30(5):233-241. doi:10.1080/10400435.2017.1317675, pmid:28708963

In addition to promoting a positive user experience, supporting self-actualizing and meaningful experiences that promote **psychological wellbeing and human potential** is

one of the main principles of positive computing and an important pillar of positive technology (eudemonic level) [25-27].

Mirkovic J, Jessen S, Kristjansdottir OB, Krogseth T, Koricho AT, Ruland CM. Developing Technology to Mobilize Personal Strengths in People with Chronic Illness: Positive Codesign Approach. *JMIR Form Res*. 2018;2(1):e10774. doi:10.2196/10774, pmid:30684404

We envisage the use of a contemporary and flexible evaluation approach that moves beyond traditional effectiveness designs to consider alterative **hybrid** trial designs [51] or multidimensional and **whole-of-system evaluation approaches**, such as a **benefits evaluation** [52] that is best aligned to the Australian digital ecosystem and positions these digital tools for real-world implementation [51]. The adaptation of user-centered design and implementation science methods from inception (such as done here) can mitigate the risk of low rates of implementation and the associated research waste [19]. In this context, we gained valuable insights from young people to support and enable implementation, including taking a whole-of-health (systems, services, and clinical-level) approach to facilitate real-world dissemination and embedding.

Slater H, Stinson JN, Jordan JE, et al. Evaluation of Digital Technologies Tailored to Support Young People's Self-Management of Musculoskeletal Pain: Mixed Methods Study. *J Med Internet Res*. 2020;22(6):e18315. doi:10.2196/18315, pmid:32442143

After the pilot, participants were enthusiastic about the offline modules, but did not use the online modules as intended. Although they had come up with a solution (the LIFE exercise group), our initial idea to promote self-management in this way, did not work out well. This result emphasizes the importance of testing (parts of) the platform in a very early stage.

van Bruinessen I, van 't Klooster J-W, Boessen A, van der Heide L, Vollenbroek-Hutten M, van Dulmen S. INVOLVING END-USERS IN THE DEVELOPMENT AND IMPLEMENTATION OF A WEB-BASED, PHYSICAL ACTIVITY PLATFORM FOR ELDERLY.

Machine learning techniques and medical knowledge are used to generate alerts and suggest appropriate actions to patients, informal caregivers, and health care professionals [47].

Verhoeven F, Cremers A, Schoone M, Van Dijk J. Mobiles for mobility: Participatory design of a "Happy walker" that stimulates mobility among older people. *Gerontechnology*. 2016;15(1). https://journal.gerontechnology.org/currentIssueContent.aspx?aid=2248

However, user-centered design research could lead to stakeholder recommendations that are not consistent with evidence-based practices or theory-based principles of change. Adherence to evidence-based practices or theory-based principles of change might not always be emphasized in user-centered design research, yet it is a key activity of the design process inherent in the original user-centered design guidelines [31,32].

Vilardaga R, Rizo J, Zeng E, et al. User-Centered Design of Learn to Quit, a Smoking Cessation Smartphone App for People With Serious Mental Illness. *JMIR Serious Games*. 2018;6(1):e2. doi:10.2196/games.8881, pmid:29339346

Agile Digital Health

In the future, researchers can consider including a user experience (UX) designer to facilitate the agile process and to video record piloting sessions in order to systematically code and evaluate users' experience, similarly to the methodology applied in Mansson et al.'s(2020) co-creation of a smart phone application with older adult end users. Although analytic data were monitored to track program progress in Study 2, future avenues for research include analyzing data to further investigate any areas of navigation difficulty and whether there are any associations between analytics (e.g., average length of time between the completion of various modules) and program benefits.

Study 2 had 50 per cent attrition across the two sequential pilots. This was not unexpected, however, because online interventions tend to have higher rates of attrition than in-person interventions (Eysenbach, 2005; Kelders et al., 2012; Peels et al., 2012). High attrition rates may occur for a variety of reasons such as the fleeting or "surfing" culture of the Internet (Ahern, 2007). It may be that participants feel a greater sense of responsibility or investment when participating in person as there is more rapport established between intervention facilitators and other group members. According to Eysenbach (2005), attrition is "one of the fundamen- tal characteristics and methodological challenges in the evaluation of eHealth applications" (p. 2).

Therefore, this is an area in need of future systematic research; for example, using a feedback questionnaire designed to understand the reasons for discontinuing an online program.

Another important limitation of the described agile develop- ment cycle is the extensive use of time and resources. From the first translational phase to the completion of Study 2 (T3 Translation to Patients phase), the project spanned more than 3 years and involved a large multidisciplinary team of researchers, clinicians, e-learning designers, patient advisors, research assistants, and administrative staff.

Yusupov I, Vandermorris S, Plunkett C, Astell A, Rich JB, Troyer AK. An Agile Development Cycle of an Online Memory Program for Healthy Older Adults. *Can J Aging*. 2022;41(4):647-656. https://www.scopus.com/inward/record.uri?eid=2-s2.0-85126456288&doi=10.1017%2fS0714980821000763&partnerID=40&md5=10b83e08ed545c8f0f44eeaa25627685

Lastly, this study reports on only the design process and usability, but for the successful uptake and implementation of an intervention on a larger scale, other aspects of

the intervention must also be evaluated, such as the acceptability of its use, the barriers and facilitators to its use, the feasibility of the intervention, and its efficacy.

O'Connor A, Tai A, Brinn M, Thuc Hien Hoang N, Cataldi D, Carson-Chahhoud K. Co-design of an Augmented Reality Asthma Inhaler Educational Intervention for Children: Development and Usability Study. *JMIR Pediatr Parent*. 2023;6. https://www.scopus.com/inward/record.uri?eid=2-s2.0-85167715240&doi=10.2196%2f40219&partnerID=40&md5=cc349ac6ff0a82035b84d2018702298b

It is important to note that the ideas generated through co-design with layman users may not always be clinically sound, and additional attention should be placed on issues such as privacy, data ownership and ethics.

Liang Z, Melcer E, Khotchasing K, Hoang NH. Co-design personal sleep health technology for and with university students. *Front Digit Health*. 2024;6. https://www.scopus.com/inward/record.uri?eid=2-s2.0-85191036091&doi=10.3389%2ffdgth.2024.1371808&partnerID=40&md5=8f5ef4e6d87e2f109fef9327baa66073

Thus, although a user-centered design process that is based on theory and evidence may contribute to successful implementation, the implementation process needs to account for the multiple complexities in the healthcare system, in which unpredictability is ever present [68]. The challenges we experienced may reflect a lack of sufficient managerial support and resources for implementation, which are common challenges in the implementation of eHealth services for self-management [69].

We believe that the user needs that we identified will be applicable in other chronic care settings. However, we acknowledge that older adults are an extremely hetero-geneous group with highly varied characteristics and needs, who use, modify, and interact with technologies in rather diverse ways [70]. The merits of user involvement include learning, adjusted designs, and achieving a sense of participation among older users [49].

Ekstedt M, Kirsebom M, Lindqvist G, et al. Design and development of an ehealth service for collaborative self-management among older adults with chronic diseases: A theory-driven user-centered approach. *Int J Environ Res Public Health*. 2022;19(1). https://www.scopus.com/inward/record.uri?eid=2-s2.0-85121849256&doi=10.3390%2fijerph19010391&partnerID=40&md5=e667f87cff3dab67cfc8d6ae3cc1d125

In the domain of intervention research, end-user requirements have been mainly explored though qualitative focus groups and surveys. [42,48,49] While these studies provide us with valuable insights, their methodologies are inherently retrospective. A strength of the prospec- tive, iterative SCREAM method is the direct translation of knowledge into a solution – learn as you go – to ensure that all stakeholders can repeatedly learn from each other. [36] Truly understanding the H2H process for CMC families and the role of each stakeholder within that process is the first step towards improving it. Certain Design Thinking methods used in this study, such as a co-creation session and sprint re- views, allowed all stakeholders to share their perspectives simulta- neously. This way, their

respective roles during the H2H transition could be clarified and they were given the agency to communicate their needs through a continuous loop of communication. Another strength of this iterative method lies in its ability to generate long-term effects through the learning process itself. Each team member contributes their equally important expertise, whether grounded in scientific principles, drawn from personal experiences, or focused on the social context of the rele- vant issue – in this case the H2H transition for CMC families. As a team, we gained new insights from each other's perspectives and collectively redefined the issue, encompassing not only the medical aspects, but also the challenges of social isolation and care coordination. This type of transdisciplinary science then aims to create a sustainable outcome, that is applicable within the social context in which the problem occurs. [50,51]

Inherent to this collaboration and the implementation of the SCREAM approach by the AUAS, is the structure of a 20-week project, with sprints that last two to three weeks on average. The time constraints may have restricted the maturity of the final prototype. Yet, this limitation is inherent to an iterative approach, where development is an ongoing process without a definitive endpoint.

van de Riet L, Aris AM, Verouden NW, et al. Designing eHealth interventions for children with complex care needs requires continuous stakeholder collaboration and co-creation. *PEC Innov*. 2024;4. https://www.scopus.com/inward/record.uri?eid=2-s2.0-85189314708&doi=10.1016%2fj.pecinn.2024.100280&partnerID=40&md5=da892ff87e1dc0c8e6281027430af68e

In fact, very few studies mention the presence of designers on their teams and mostly focus on involving end users [11]. The challenge in these types of projects is how to properly incorporate users'needs into the project, redefine them properly, and bridge them while the project evolves. Design expertise leadership is a key component in achieving that goal since design practice and research focus on users' unmet needs through well-understood and applied inclusive design approaches coming from design expertise [54] In fact, very few studies mention the presence of designers on their teams and mostly focus on involving end users [11].

assess the viability of the solution, develop a business model for the app, and plan the implementation of the technology [42,58]. This points to the importance of transdisciplinary work in the development of e–mental health solutions from a human-centered design perspective [59]. This is why the Mentallys project relies on other research and development activities complementary to co-design: a full design team, including branding design experts and user experience and user interface designers; a business accelerator program and business model definition coaching; a partner technology company that cosupervises the software development; and several partner health institutions to conduct real-world testing.

Vial S, Boudhraâ S, Dumont M, Tremblay M, Riendeau S. Developing A Mobile App With a Human-Centered Design Lens to Improve Access to Mental Health Care (Mentallys Project): Protocol for an

Initial Co-Design Process. *JMIR Res Protoc*. 2023;12. https://www.scopus.com/inward/record.uri?eid=2-s2.0-85169844139&doi=10.2196%2f47220&partnerID=40&md5=712b8aba8ba36c9295129b0abe6b8efc

Despite this planning effort, CAPTAIN's new method- ology still requires multiple adaptation and revision, and a higher capability in swift problem solving

Tessarolo F, Petsani D, Conotter V, et al. Developing ambient assisted living technologies exploiting potential of user-centred co-creation and agile methodology: the CAPTAIN project experience. *J Ambient Intell Humaniz Comput*. 2024;15(5):2661-2676. https://www.scopus.com/inward/record.uri?eid=2-s2.0-85127725459&doi=10.1007%2fs12652-021-03649-0&partnerID=40&md5=de93ad22dd6812331475b53229fc9fa6

By involving survivors, health care professionals, and managers in codevelopment activities (eg, surveys, in-depth interviews or focus groups, usability tests, and field trials), interventions could be designed to address stakeholders' real needs and development could better align with their practices and contexts, thereby increasing the odds of successful implementation. Second, involving interdisciplinary teams in the development process is key to ensure that comprehensive solutions are developed and design caveats are timely anticipated, identified, and refined, thus not compromising the usefulness, usability, and feasibility of such programs.

Barros ACD, Bergmans M, Hasanaj K, et al. Evaluating the User Experience of a Smartphone-Delivered Sexual Health Promotion Program for Older Adults in the Netherlands: Single-Arm Pilot Study. JMIR Hum Factors. 2024;11(1). https://www.scopus.com/inward/record.uri?eid=2-s2.0-85192202309&doi=10.2196%2f56206&partnerID=40&md5=3f6ad460e299d8bbed78e44510ae6043

The present series of studies and the creation of a new digital health tool resulted from close collaboration between behavioral and computer scientists. This type of collaboration is becoming more common and desirable to ensure that the development of a tool to support health behavior change is informed by a range of relevant expertise [74].

In this study, although we developed the storyboard and specifications for the web application over several months and a series of iterations, potentially important navigation rules were left unspecified (ie, use of the web browser's back button, entry of spaces, or nonlanguage text as open-ended responses). These and similar oversights may be owing to behavioral scientists' implicit assumptions and lack of familiarity with the problems that such errors can cause, and thus, failing to specify all desired rules ahead of time. To avoid the need for major changes after the initial tool is built—particularly if funding for such changes is limited or uncertain—there is additional pressure on computer science team members to foresee all types of possible pitfalls. In an academic setting, where funding for the development of digital tools may be scarce, early discussions about how and when substantive, unforeseen changes will be handled are critical.

Behavioral scientists also tend to focus, from the beginning, on a process of human subjects research that involves regulatory oversight, informed consent discussions, certain types of documentation (eg, test user feedback), and multiple attempts to secure additional (limited) funding. This process is intentionally methodical and can be quite slow as a result [74]. Computer scientists may not be familiar with the IRB processes, training required to participate in studies with human participants, and delays that these may cause. Computer scientists may prefer agile and iterative software development methods with multiple rounds of user feedback, but may not realize, at the beginning, the time and effort required to obtain IRB approvals and recruit participants. For this collaboration, although we were aware of the potential regulatory challenges from the beginning, we encountered difficulties regarding questions about permissions for server housing and IRB questions about the collection of protected health information via the new web application.

Arigo D, Lobo AF, Ainsworth MC, Baga K, Pasko K. Development and Initial Testing of a Personalized, Adaptive, and Socially Focused Web Tool to Support Physical Activity among Women in Midlife: Multidisciplinary and User-Centered Design Approach. *JMIR Form Res*. 2022;6(7). https://www.scopus.com/inward/record.uri?eid=2-s2.0-85136904369&doi=10.2196%2f36280&partnerID=40&md5=f3e9451c23e67583256d653828df1b40

In our study, the participants worked to design and develop a technology for future users. During the process, the participants learned from each other's fields of expertise through the use of personas. The IT specialists and HCPs learned about patients' and family members' needs and, conversely, the patients and HCPs learned from the IT spe- cialists about possibilities in developing technology. Moreover, continually sampling new patients enabled us to validate and adjust the information system. However, as different patients were involved throughout the process, the iterations implemented by the researchers were crucial to including the perspectives of the patients in the prototype. Simonsen and Robertson highlight26 ethical reflections on PD and its usability, noting that researchers must carefully consider the involvement of par- ticipants to ensure genuine involvement and mutual learn- ing.26

In the planning stage, we had a preconception that physical participation in the workshops would be preferred due to the core elements of PD. However, we found the iterative process, which involved the researcher acting as a messenger between the workshops and the patients and family members, to be a viable alternative for implementing the codesign process. The participatory approach is also seen as a strength, as it may have improved system quality due to better, more accur- ate user requirements, thus providing a greater likelihood of the inclusion of features that users actually want.13

Østervang C, Lassen A, Schmidt T, Coyne E, Dieperink KB, Jensen CM. Development of a health information system to promote emergency care pathways: A participatory design study. *Digit Health*. 2022;8. https://www.scopus.com/inward/record.uri?eid=2-s2.0-85145049008&doi=10.1177%2f20552076221145856&partnerID=40&md5=a20dfea4e7c842fb077d606db1f924f4

Although more research is needed, emerging qualitative research with healthy and cognitively impaired populations can provide a more nuanced examination of patients', caregivers', and providers' attitudes and preferences for cognitive screening [16,71,76,77]. Older adults' perspectives on cognitive screening are multifaceted and diverse, and a human-centered approach to developing screening paradigms is imperative to prevent stigma and improve early detection [16,77].

Although more research is needed, emerging qualitative research with healthy and cognitively impaired populations can provide a more nuanced examination of patients', caregivers', and providers' attitudes and preferences for cognitive screening [16,71,76,77]. Older adults' perspectives on cognitive screening are multifaceted and diverse, and a human-centered approach to developing screening paradigms is imperative to prevent stigma and improve early detection [16,77].

Young SR, Lattie EG, Berry ABL, et al. Remote Cognitive Screening of Healthy Older Adults for Primary Care with the MyCog Mobile App: Iterative Design and Usability Evaluation. *JMIR Form Res*. 2023;7. https://www.scopus.com/inward/record.uri?eid=2-s2.0-85149135290&doi=10.2196%2f42416&partnerID=40&md5=bcc2ac67d034f8bd33110e924e3913e4

It should also be noted that there was a lack of diversity in the clinician sample (all were female and drawn from the same clinic), and this may have limited representativeness of clinician feedback.

It should also be noted that there was a lack of diversity in the clinician sample (all were female and drawn from the same clinic), and this may have limited representativeness of clinician feedback.

Deady M, Collins D, Gayed A, Harvey SB, Bryant R. The development of a smartphone app to enhance post-traumatic stress disorder treatment in high-risk workers. *Digit Health*. 2023;9. https://www.scopus.com/inward/record.uri?eid=2-s2.0-85150077712&doi=10.1177%2f20552076231155680&partnerID=40&md5=807953d32125a0a6272cb256f2965726

Digital health care interventions do not exist in isolation; they are integrated into existing clinical pathways, so designing and gaining feedback on the whole patient journey as per HCD principles is important.

As the number of individuals involved in a co-design process is often limited, there is a potential risk that design outputs can ignore important evidence-based ideas.

Donovan G, Hall N, Smith F, Ling J, Wilkes S. Two-way Automated Text Messaging Support from Community Pharmacies for Medication Taking in Multiple Long-term Conditions: Human-Centered Design with Nominal Group Technique Development Study. *JMIR Form Res*. 2022;6(12). https://www.scopus.com/inward/record.uri?eid=2-s2.0-85145550708&doi=10.2196%2f41735&partnerID=40&md5=887a766b53d9f6ca4578efdbc7362154

work is that the design requirements and evaluations were conducted with intended users from various representative underserved communities in South Texas and the result may not generalize to all underserved populations.

Bonet-Olivencia S, Carrillo-Leal J, Rao A, Sasangohar F. User-Centered Design of a Diabetes Self-Management Tool for Underserved Populations. *J Diabetes Sci Technol*. 2024;18(1):22-29. https://www.scopus.com/inward/record.uri?eid=2-s2.0-85177045933&doi=10.1177%2f19322968231212220&partnerID=40&md5=6105451b51ec2c2947207410e8392085

First, the definition of personas did not follow a structured cluster analysis as reported in other studies.42

Instead, our definition of personas was based on interviews with poten- tial app users and subsequent discussions within our inter- disciplinary team. Second, the majority of our test users reported previous CVD. Thus, the needs and interests of individuals in primary prevention might be underrepre sented in HerzFit. Third, the usability testing was per- formed with a prototype of our final version of HerzFit. Consequently, the usability evaluation results might no longer reflect HerzFit ́s current performance. Fourth, there may have been selection bias in HerzFit ́s usability evalu- ation. Study participants wanted to use the app and might have given biased feedback. Finally, there has been no stat- istical evaluation of HerzFit, its user structure, and its impact on risk factor control and lifestyle behavior so far.

Reimer LM, Nissen L, von Scheidt M, et al. User-centered development of an mHealth app for cardiovascular prevention. *Digit Health*. 2024;10. https://www.scopus.com/inward/record.uri?eid=2-s2.0-85193707138&doi=10.1177%2f20552076241249269&partnerID=40&md5=be01eb98edaf0c09ecce6f5ce5ff91d7

This study has several limitations. First, our user-centered development process was meant to solicit detailed guidance from caregivers with lived experience; study conclusions are drawn from small samples and may not generalize to all members of this population. Furthermore, given the fact that recruitment occurred primarily on the internet, participants may be drawn from a population that is already predisposed to digital tools. Second, design objectives for Bolster were constrained to some extent by predefined project goals. Bolster was a priori proposed as a self-guided mobile intervention. Thus, caregiver feedback related to intervention features that could require clinical support (eg, an expert question-and-answer feature) or social features (eg, peer forums) was incorporated into Bolster in a manner consistent with these a priori constraints (eg, providing clinician videos rather than synchronous communication and links to support websites rather than the development of new tools).

Buck B, Wingerson M, Whiting E, Snyder J, Monroe-DeVita M, Ben-Zeev D. User-Centered Development of Bolster, an mHealth Intervention for Early Psychosis Caregivers: Needs Assessment, Prototyping, and Field Trial. *JMIR Ment Health*. 2023;10. http://dx.doi.org/10.2196/50522

Despite these strengths, our development process also had several limitations, among them the inability to include all features discussed during the FGs owing to limited resources, such as 2-way communication between users and HCPs and remote monitoring of progress by HCPs. To create an app that closely resembled the overall vision of the participants, the research team, with guidance from the app development team, prioritized features that emerged from the FGs before the agile development phase .Involving stakeholders in the feature prioritization activity and sprints during agile development would allow for their direct input on decisions regarding feature prioritization and resource allocation. Most participants in FG 3 (user testing; 19/24, 79%) also participated in at least 1 of the 2 previous FGs, which may have led to bias as the features tested were those that participants had suggested previously.

Selection bias from voluntary response sampling could have led to an overrepresentation of participants who were interested in mobile health and highly motivated to respond positively to the app, as well as those with higher socioeconomic status who had the ability to travel and the time to attend the FGs.

A potential limitation of the app's feasibility is its low compliance with health behavior tracking.

With family involvement (eg, family challenges and daily challenges that involve parental support) being an integral part of our app, there is the potential for children to track their health behaviors together with their parents, especially those who do not have their own mobile device, which may promote increased adherence.

Yau KW, Tang TS, Görges M, Pinkney S, Amed S. Using Human-Centered Design and Cocreation to Create the Live 5-2-1-0 Mobile App to Promote Healthy Behaviors in Children: App Design and Development. *JMIR Pediatr Parent*. 2023;6. https://www.scopus.com/inward/record.uri?eid=2-s2.0-85159837500&doi=10.2196%2f44792&partnerID=40&md5=fd2e5c222767b1ed1614b242d8748135

It is worth noting that the use of low fidelity prototypes im- posed limitations on what we could test. Even so, a previous study comparing high and low fidelity prototypes in game de- sign did not support a difference in the number of usability is- sues identified between prototype fidelities, and recommended the use of lower fidelity prototypes to allow more rapid iteration and to keep costs comparatively low.[19]

The varied responses to the user interface design during A/B testing introduces the possibility of including a setting that al- lows users to tweak the presentation format.

Sunjaya AP, Martin A, Jenkins C. A Design Thinking Approach to Developing a Clinical Decision Support System for Breathlessness in Primary Care...18th World Congress of Medical and Health Informatics, MedInfo 2021 - One World, One Health – Global Partnership for Digital Innovation, 2-4 October, 2021. *Stud Health Technol Inform*. 2022;290:839-843.
https://search.ebscohost.com/login.aspx?direct=true&db=ccm&AN=157572072&site=ehost-live

Owing to the qualitative research design, it is not possible to quantify or generalize the usability problems identified to other health technologies and settings. In addition, translation of our results to other hospital settings or countries is limited because of the single-center design of this study and the relatively small sample size. It was not possible to draw samples randomly, which needs to be considered as a potential source of bias when interpreting the results. The comparison between design A, which was a working medical product installed in the ICU, and design B, a prototype mock-up, may be potentially unfair with a number of confounders in the 2 arms.

Poncette A-S, Mosch LK, Stablo L, et al. A Remote Patient-Monitoring System for Intensive Care Medicine: Mixed Methods Human-Centered Design and Usability Evaluation. *JMIR Hum Factors*. 2022;9(1).
https://www.scopus.com/inward/record.uri?eid=2-s2.0-85126621683&doi=10.2196%2f30655&partnerID=40&md5=cf5180ad52ea2c03786bd8871c7789c0

Thus, it is crucial to promote user acceptability testing early in the design process as it may mitigate later financial deficits because of failing to meet the users'needs and wishes for functionalities before making significant investments [22]. As the expected user population may fall short, predicting user acceptance may not only save significant losses but also guide important decisions in the design process.

Preuhs K, van Keulen H, Andree R, Wins S, van Empelen P. A Tailored Web-Based Video Intervention (ParentCoach) to Support Parents With Children With Sleeping Problems: User-Centered Design Approach. *JMIR Form Res*. 2022;6(4).
https://www.scopus.com/inward/record.uri?eid=2-s2.0-85129587847&doi=10.2196%2f33416&partnerID=40&md5=33159baa413482c0e4dbc2ee3f29d814

By combining methodologies from different fields, we adopted a highly interdisciplinary approach, the lack of which has been highlighted as a significant limitation in existing digital mental health interventions. We hope that in doing so, we increased the potential for sufficient user engagement while also providing a sound evidence base for the content of the intervention [12,15].

By combining methodologies from different fields, we adopted a highly interdisciplinary approach, the lack of which has been highlighted as a significant limitation in existing digital mental health interventions. We hope that in doing so, we increased the potential for sufficient user engagement while also providing a sound evidence base for the content of the intervention [12,15].

In relation to that, it should be noted that the research team did not collect specific demographic or other sensitive category data, which could be useful in interpreting

Throughout the development process, we noticed a tension between finding the right balance between guaranteeing an adequate evidence base for each feature of the intervention and leaving enough room for creativity and innovation of new features. We strongly agree that evidence-based and evidence-informed concepts are of significant importance; however, there seems to be a common misconception that one can only achieve evidence-based innovation by transforming evidence-based nondigital interventions into digital ones [17]. We would like to encourage the field to free itself from this notion as it can significantly hinder us from reaching the actual potential of digital mental health interventions [71].

Moltrecht B, Patalay P, Bear HA, Deighton J, Edbrooke-Childs J. A Transdiagnostic, Emotion Regulation App (Eda) for Children: Design, Development, and Lessons Learned. *JMIR Form Res*. 2022;6(1). https://www.scopus.com/inward/record.uri?eid=2-s2.0-85124142054&doi=10.2196%2f28300&partnerID=40&md5=b30e8fd39812b9b35473d717e36e6731

Community crowdsourcing to create useful and credible health information resources has been found to have more reliability when professionals or experts are present in the process of content creation.

Whether community users can judge credibility is also debated. There is research suggesting that people often report judging source credibility, but observational studies suggest otherwise [102-104].

Morse B, Soares A, Kwan BM, et al. A Transgender Health Information Resource: Participatory Design Study. *JMIR Hum Factors*. 2023;10. https://www.scopus.com/inward/record.uri?eid=2-s2.0-85164694447&doi=10.2196%2f42382&partnerID=40&md5=075a8c6eb6239d39654fdef23c12c000

Considering that the agile development cycle is adapted from the technology sector, we combined this process with the framework from the Harvard Clinical and Translational Science Center's five translational (T) phases that are necessary to execute before a health intervention can become available to the general public (Harvard Catalyst, 2021).

Yusupov I, Vandermorris S, Plunkett C, Astell A, Rich JB, Troyer AK. An Agile Development Cycle of an Online Memory Program for Healthy Older Adults. *Can J Aging*. 2022;41(4):647-656. https://www.scopus.com/inward/record.uri?eid=2-s2.0-85126456288&doi=10.1017%2fS0714980821000763&partnerID=40&md5=10b83e08ed545c8f0f44eeaa25627685

1. Anticipate the time and financial resources needed to facilitate the participation of nurses and physicians [10]. As this case study was conducted as part of the Exucheck project, associating the health care professionals as partners in the definition of the project

was key to their involvement. 2. Be available. This made it possible to conduct observations throughout the nurses' and physicians'work, regardless of their working hours. When integrating a new medical device into a department, the atmosphere and tasks differ over the 24-hour period; this can have an impact on how a device is used and on nurses'and physicians'interactions with the system. By appointing 2 usability engineers to work together on the project, a large number of situations could be observed and the results could be compared.

3. Be reactive. An evaluation kit that was always ready and transportable was an asset when conducting user tests. This kit allowed rapid access to the field to exchange views with the team on a last-minute slot and bring the device evaluation tools as close as possible to the nurses' and physicians' workplace.

4. Adapt the organization of user tests. Nurse–nurse assistant pairs were involved, which helped their projection into conceptual innovations. As a result of

shared awareness

[35] between users, new ideas were more forthcoming and converged more rapidly toward a solution through a limited number of tests. A clinical team should share their practices to help in designing technologies for the team. However, bias can occur as a result of hierarchical relationships within the team conducting tests. The usability engineers must remain vigilant during tests to ensure that they are gathering all points of view.

One of the recognized limitations of UCD is that it considers only end user feedback for design choices and addresses technical constraints in parallel. The co-design approach that we used enabled us to remain as vigilant as possible on this point and to continually confront the needs and expectations of users regarding the technical constraints. However, we note that on certain aspects, notably the attachment of the system to the patient, our methodology has not yet succeeded in proposing a convincing solution. This shows that the user does not always have answers to the problems and that the proposed solutions are sometimes technically unfeasible. Therefore, it is important to set up new design loops involving the end user and the technical team in the design choices. This is especially true as users can evolve, change their minds, gain expertise, and transform their practice. Therefore, our role is to keep a critical eye on their feedback and be open to any request for change. It is also important to bear in mind that every technological advance can call an initial user need into question and vice versa.

Schwartz-Lasfargues C, Roux-Gendron C, Edomskis P, et al. Development of a Connected Sensor System in Colorectal Surgery: User-Centered Design Case Study. *JMIR Hum Factors*. 2022;9(3). https://www.scopus.com/inward/record.uri?eid=2-s2.0-85134464461&doi=10.2196%2f31529&partnerID=40&md5=8070ba7c4a725b45ff5454d754541029

Although negotiation acted as a linchpin for shared decision-making [60], our analysis identified how articulation, lack of knowledge, unfulfilled expectations, and nonreciprocity inhibited negotiation and increased tensions in the collaborative space.

Literature highlights that negotiation is an essential component in shared health decision-making [60,96]. Our matrix analysis identified how adolescents' articulation, memory for exercises, parent's knowledge, the long trajectory of knee pain, and GP's ability to engage with adolescents inhibited negotiation and shared learning and increased tensions within the collaborative space.

Furthermore, having people working in groups may have made the process more open to say-do problems, compared with single-person interviews [103].

Johansen SK, Kanstrup AM, Haseli K, Stenmo VH, Thomsen JL, Rathleff MS. Exploring User Visions for Modeling mHealth Apps Toward Supporting Patient-Parent-Clinician Collaboration and Shared Decision-making When Treating Adolescent Knee Pain in General Practice: Workshop Study. *JMIR Hum Factors*. 2023;10. https://www.scopus.com/inward/record.uri?eid=2-s2.0-85159889608&doi=10.2196%2f44462&partnerID=40&md5=0aa75ddf6bbcef3ebeea957e3fe8d138

Other examples of co-design tools and activities suitable for young people include post-it notes, mapping activities, brainstorm clouds, whiteboards, games, card sorting and storytelling.38,42

Using a variety of methods also allows for triangulation of data,52 providing a more comprehensive under- standing of young people's needs and preferences for a digital mental health platform.

Recommendation 3: Structure co-design workshops based on participants' age. It is recommended that separate co-design workshops are conducted for different age groups. Structuring workshops by age of participants fosters an environment where participants are more likely to be matched on their comprehension and language abil- ities and psychosocial characteristics.53 It may also reduce power imbalances between younger and older participants.

While co-design methodology is anticipated to enhance end-user engagement with DMHIs, it is an assumption that this approach results in greater com- pletion of intervention content or better clinical outcomes, and therefore, future research is needed to empirically assess this supposition.

Ludlow K, Russell JK, Ryan B, et al. Co-designing a digital mental health platform, "Momentum", with young people aged 7–17: A qualitative study. *Digit Health*. 2023;9. https://www.scopus.com/inward/record.uri?eid=2-s2.0-85178199830&doi=10.1177%2f20552076231216410&partnerID=40&md5=29aecb0f62f1325861b914ce5aee4d07

Future development should set a limit on the number of UI changes that can be made after the initial designs have been approved. Scope creep is a known challenge in agile development environments [16].

Liu S, La H, Willms A, Rhodes RE. A “No-Code” App Design Platform for Mobile Health Research: Development and Usability Study. JMIR Formative Research. 2022;6(8):e38737. Accessed April 15, 2024. https://formative.jmir.org/2022/8/e38737

The development of more nuanced experimental approaches that enable evaluation alongside ongoing and continuous adaptation of systems could facilitate simultaneous development and rigorous evaluation of HIT systems. This challenge echoes literature on the development of quality improvement interventions, with the need to reconcile pragmatism (eg, the generation of HIT systems by software developers) and research rigor (eg, understanding the underlying mecha- nisms of HIT interventions and the influence of contextual factors)

Allsop MJ, Johnson O, Taylor S, et al. Multidisciplinary Software Design for the Routine Monitoring and Assessment of Pain in Palliative Care Services: The Development of PainCheck. *JCO Clin Cancer Inform*. 2019;3:1-17. doi:10.1200/CCI.18.00120, pmid:31577449

Historically, pressure to identify effective interventions in public health has led to many interventions being tested for effectiveness in **randomized controlled trials (RCTs),** with little rigorous development work being completed [46]. This has led to large amounts of resources being invested in interventions that are unlikely to be effective. **However, frameworks for the development and evaluation of complex interventions [19] stress the importance of the development phase, which is at least as important as the effectiveness evaluation stage.** Proper development involving users, relevant theory, and research evidence is more likely to produce an intervention that is acceptable and feasible to deliver and also potentially effective.

In the world of digital health, this development phase is also crucial, and user-centered design is a common approach used [20-22]. As more digital health apps and interventions are being developed, one issue that is important to consider is whether these interventions should be tested in RCTs or whether other evaluation methods should be used. Given the pace of development of digital health, evaluation methods are needed that are **more agile** and more user-centered than the standard RCT methodology, which may take too long to produce the results needed.

Bevan Jones R, Thapar A, Rice F, et al. A Web-Based Psychoeducational Intervention for Adolescent Depression: Design and Development of MoodHwb. *JMIR Ment Health*. 2018;5(1):e13. doi:10.2196/mental.8894, pmid:29449202

Use **shorter-cycle iterative research sprints with smaller sample sizes instead**.

Cornet VP, Toscos T, Bolchini D, et al. Untold Stories in User-Centered Design of Mobile Health: Practical Challenges and Strategies Learned From the Design and Evaluation of an App for Older Adults With Heart Failure. *JMIR Mhealth Uhealth*. 2020;8(7):e17703. doi:10.2196/17703, pmid:32706745

Some patients participated during several sprints and therefore became 'co-researchers' or 'research partners' in the development process, having a decisional role (Symposium Panta Rhei, TU Delft).

Most HCPs participated once, only one HCP participated twice. It would be interesting to explore the influence of these different de- grees of participating in future studies

Another question one needs to ask, when involving end-users using scrum, is if these end-users (i.e. patients and providers) are able to reflect upon a tool 'in development'. Especially early in the process, 'ListeningTime' consisted of bits of (technical) information and the lay-out was not very presentable. This made it difficult to see how a 'final' version of ListeningTime could become.

In addition, end-users may not know what kind of intervention they exactly prefer or need until it is right in front of them.

Although researchers and software developers both had the aim to develop a user-centered tool, the requirements of the software devel- opers did not always coincide with scientific ones.

The short sprints were useful for the software developers, but a challenge for the re- searchers as they had to develop and deliver all the content materials and recruit and involve end-users at the same time. In addition, for scientific research it is important to include a representative sample, as far as conceivable, of patients and providers to develop the tool.

Involved patients, HCPs and their representatives were positive about (the way of) involvement in developing the tool. Attracting in- volved end-users may be a precondition in developing a successful in- tervention (van Bruinessen et al., 2014). However, it is also important to develop an intervention that reaches out to the entire population of end-users.

Altogether, scrum may not be the best framework for developing an innovative tool like ListeningTime. This was also debated in a recent symposium, where they concluded that scrum and working agile 'hin- ders or obstructs true innovation', because of too much focus on quick development and learning by doing (Arnstein, 1969). The recently published IDEAS framework (Mummah et al., 2016), may be an interesting alternative for co-creation between non-profit scientific re- search and for-profit (development) companies, involving end-users. According to the authors: "the IDEAS framework strives to provide sufficient detail without being overly prescriptive so that it may be useful and readily applied by both investigators and industry partners in the development of their own mHealth, eHealth, and

other digital health behavior change interventions” (Mummah et al., 2016). Never- theless, the ‘pressure cooking situation’, by using scrum, resulted in a quick development process and a ‘final’ product. In contrast to tradi- tional (research) methods where more time investment is needed for perhaps a similar result.

Noordman J, Driesenaar JA, van Bruinessen IR, van Dulmen S. ListeningTime; participatory development of a web-based preparatory communication tool for elderly cancer patients and their healthcare providers. *Internet Interv*. 2017;9:51-56. doi:10.1016/j.invent.2017.05.002, pmid:30135837

Another important contribution this study made to research in the field is the documentation of a rigorous and innovative build process, which combined intervention development method and participatory research methodologies throughout its life cycle [69]. Across the spectrum of eHealth intervention build methods, **we recognize our participatory research method being in the middle ground of the 2 polar approaches: agile or technologist-driven and carer-led. Our approach establishes itself as a third way adopting the agile process and principles while integrating PPI with the technological and research core team conducting the build work as directed by the EAG.**

Sin J, Henderson C, Woodham LA, Sesé Hernández A, Gillard S. A Multicomponent eHealth Intervention for Family Carers for People Affected by Psychosis: A Coproduced Design and Build Study. *J Med Internet Res*. 2019;21(8):e14374. doi:10.2196/14374, pmid:31389333

The gold standard for testing complex interventions has traditionally been the randomised controlled trial (RCT). One of the methodological challenges in the field of digital health interventions is how to evaluate them when the field moves at such a swift pace. RCTs are time- and resource-intensive meaning a technology could be superseded or become obsolete before the end of a trial. In the field of mHealth, the iterative development process (with new releases and bug fixes) and personalisation of apps are not readily accommodated within the traditional RCT model whereby a rigid protocol is typically followed to assess a static intervention (Mohr et al., 2017; Murray et al., 2016; Pham et al., 2016). Thus traditional RCTs may often be an impractical evaluation approach for digital health interventions. Mohr et al. (2018), and Wilson et al. (2018) suggest we need to consider more agile and efficient approaches to mHealth development and evaluation lifecycles. Alternative frameworks and evaluation methods have been proposed that allow iterative changes to be made (West and Michie, 2016). Hybrid trial designs that combine evaluation of effectiveness and implementation potentially speed up the translation of research findings into real-world practice and increase the likelihood of successful uptake and adoption (Mohr et al., 2017). We will consider alternative, more agile methodological approaches when designing and planning future optimisation, evaluation and imple- mentation phases of MS Energise.

van Kessel K, Babbage DR, Kersten P, et al. Design considerations for a multiple sclerosis fatigue mobile app MS Energize: A pragmatic iterative approach using usability testing and resonance checks. *Internet Interv*. 2021;24:100371. doi:10.1016/j.invent.2021.100371, pmid:33614414

Finally, we benefited from having a project-lead who is a clinician familiar with the healthcare context and was involved in each research phase. During the software feedback cycle conducted independently with the software developer, **the project-lead acted as an advocate for patients**, family members and clinicians involved in the previous phases to ensure that the user- and clinician- requirements were addressed as the application was built. **Digital health designers need to document case studies and experiences to advance the knowledge base for in-hospital co-design of mHealth solutions.** In our example, the participatory co-design practices conducted by a clinician aimed to limit the burden on the volunteer team members but capitalise on their skills and perspectives.

Whilst end-users of consumer applications are patients themselves, healthcare teams need to lead design and development procedures in order to endorse such digital health technologies alongside current healthcare delivery.

Woods L, Cummings E, Duff J, Walker K. Conceptual Design and Iterative Development of a mHealth App by Clinicians, Patients and heir Families. http://dx.doi.org/10.3233/978-1-61499-890-7-170

Using the BCW method along with a UCD is comparable with the methodology used by Curtis et al [22] to develop a theory-driven and user-centered healthy eating app. Their work also focused on a thorough analysis of target behaviors, selection of BCTs, and exploration of user preferences to underpin the design of the app with relevant theory and evidence and ensure engagement among the target population.

From a health care and behavior change perspective, we chose to use the MRC framework for the development of complex interventions as a basis for our UCD, instead of a more general software development approach. The four iterative phases were inspired by the user-centered methodology used by Johnston et al [33] for the development of a Web-based interface for patients with COPD.

The use of a more general software development approach as a basis for the development process might have provided more specific guidance to the software development and usability phases beforehand. However, such approaches pay less attention to the activities needed to design a theory- and evidence-driven intervention, which was an important focus in our design process.

Essential in the development is that interventions are grounded in theory and evidence and that user needs and preferences are thoroughly investigated. Moreover, valorization and implementation activities should be regarded as continuous activities throughout the development process to ensure sustainable use in its intended practice. This extensive reporting of the intervention development process enhances the reproducibility of the intervention and contributes to more transparency in the development of complex interventions in health care, which is needed to strengthen the internal and external

validity of interventions and to add value to health care research [34]. All in all, it is helpful to have multiple examples and variants on how to develop evidence- and theory-driven mHealth interventions. It should be considered if the thoroughness of this UCD is needed for all mHealth interventions that will be developed in the future.

Depending on the topic, decisions should be made about which phases and steps are relevant to the topic and should be included in the development process. In addition, taking time aspects into consideration, it should be questioned how thoroughly an individual step should be executed.

Korpershoek YJG, Hermsen S, Schoonhoven L. User-centered design of a mobile health intervention to enhance exacerbation-related self-management in patients with chronic obstructive pulmonary .... *Journal of medical*. Published online 2020. https://www.jmir.org/2020/6/e15449/

Although the MHeC-S was comprehensible to our Colombian participants, many changes were requested. In agreement with other authors [87], we strongly advocate for the need to adapt HITs beyond language by considering cultural variations. The same authors suggest adapting or designing HITs to acknowledge cultural differences in 4 main dimensions: **content, functionality, technology platform, and user interface** [44]. **However, the methodology needed to achieve this has not been conceptualized.**

Ospina-Pinillos L, Davenport TA, Navarro-Mancilla AA, et al. Involving End Users in Adapting a Spanish Version of a Web-Based Mental Health Clinic for Young People in Colombia: Exploratory Study Using Participatory Design Methodologies. *JMIR Ment Health*. 2020;7(2):e15914. doi:10.2196/15914, pmid:32027313

While the agile science framework provides important guidance for relatively rapid and fluid develop- ment of mHealth interventions, there is little direction on maintaining internal validity and standardization of trials in the context of ongo- ing changes, upgrades, and modifications.

Consistent with the need for more transparency and guidance in mHealth interventions, **we also plan to analyze passive end user data.** This will provide an objective measure of what sections of the SCP, app features, and text messages were most attended to, as well as serve as a proxy for level of engagement—a potential moderator of outcomes and acceptability data.

Given the detailed input on the intervention development from multiple stakeholders described here, we will be able to gauge whether or not some of the preferences during the de- velopment stage were corroborated by reports of follow-up self-report acceptability data and end user data.

Thus, the detailed description of the agile development not only provides an ex- ample of methods for mHealth intervention de- velopment, but also may yield important information for interpreting data from the intervention.

In the future, it is important for those devel- oping similar interventions to disseminate de- tails about the development process and be transparent about challenges and glitches along the way, so that researchers can better traverse this process and identify shared difficulties and common solutions. Dissemination of such de- tails could lead to additional guidance and stan- dardization for the development and implemen- tation of such behavioral mHealth interventions. It also would generally enhance the rigor of mHealth research, and thus facilitate well- designed, meaningful interventions for end us- ers.

Schwartz LA, Psihogios AM, Henry-Moss D, et al. Iterative development of a tailored mHealth intervention for adolescent and young adult survivors of childhood cancer. *Clin Pract Pediatr Psychol*. 2019;7(1):31-43. http://doi.apa.org/getdoi.cfm?doi=10.1037/cpp0000272

This advocates that the environment in which PD unfolds should be thoughtfully and carefully planned, **and that par- ticipatory tools and techniques should not be applied strictly and by the book** (Brandt et al.2013). **Since there is no fixed recipe** for the application of PD to aid participation, it is important to be aware of what is accomplished using particular tools and techniques, and finding out how these in combination can create a format and procedures that create engagement and ownership of the results (Sanders et al. 2010).

What might work as a facilitating factor in one group of people may be a hindrance to another group working towards a different goal or under different circumstances (Ranmuthugala et al. 2011).

Terp M, Laursen BS, Jørgensen R, Mainz J, Bjørnes CD. A room for design: Through participatory design young adults with schizophrenia become strong collaborators. *Int J Ment Health Nurs*. 2016;25(6):496-506. doi:10.1111/inm.12231, pmid:27293176

Industry Implementability

Keywords: supply chain, system approach, downstream analysis, (UX changes with) fidelity

Supply Chain

Further research is required to fully understand the benefits of HCD in the design of **supply chain** and digital health solutions for this population. Its benefits might include fewer post-implementation iterations of tools, better uptake and acceptance of solutions, and improved sus- tainability.

Andersson SR, Hassanen S, Momanyi AM, et al. Using Human-Centered Design to Adapt Supply Chains and Digital Solutions for Community Health Volunteers in Nomadic Communities of Northern Kenya. *Glob Health Sci Pract*. 2021;9(Suppl 1):S151-S167. doi:10.9745/GHSP-D-20-00378, pmid:33727327

Through the process of codesign, we have been made aware of some of the **barriers** to implementing this app into face-to-face clinical care. **Further work will be required to tailor implementation of the app into various service settings and governance structures.** This is potentially challenging, given the different processes used across various services, and highlights that health providers and young people have different expectations and preferences with regard to the use of technology in mental health care.

Hetrick SE, Robinson J, Burge E, et al. Youth Codesign of a Mobile Phone App to Facilitate Self-Monitoring and Management of Mood Symptoms in Young People With Major Depression, Suicidal Ideation, and Self-Harm. *JMIR Ment Health*. 2018;5(1):e9. doi:10.2196/mental.9041, pmid:29362208

When aiming to transform healthcare by designing and developing an mHealth app, it is crucial that there is a willingness to implement the solution.

Ravn Jakobsen P, Hermann AP, Søndergaard J, Wiil UK, Clemensen J. Development of an mHealth Application for Women Newly Diagnosed with Osteoporosis without Preceding Fractures: A Participatory Design Approach. *Int J Environ Res Public Health*. 2018;15(2). doi:10.3390/ijerph15020330, pmid:29438343

Lean development methods aim to overcome some of those challenges; however, in practice, research and development projects cannot always keep to these more rigid cycles because of their exploratory nature and work in innovative spaces.

Martin A, Caon M, Adorni F, et al. A Mobile Phone Intervention to Improve Obesity-Related Health Behaviors of Adolescents Across Europe: Iterative Co-Design and Feasibility Study. *JMIR Mhealth Uhealth*. 2020;8(3):e14118. doi:10.2196/14118, pmid:32130179

Another challenge would be the integration of the MHeC-C with the current Colombian health care and benefit schedule, which is under the administration of several public and private institutions that use regulated government funds [98]. As there are many institutions that are involved in the provision of services, the MHeC-C would need to integrate with all of them to avoid perpetuating health inequities.

Ospina-Pinillos L, Davenport TA, Navarro-Mancilla AA, et al. Involving End Users in Adapting a Spanish Version of a Web-Based Mental Health Clinic for Young People in Colombia: Exploratory Study Using Participatory Design Methodologies. *JMIR Ment Health*. 2020;7(2):e15914. doi:10.2196/15914, pmid:32027313

In our study, it was evident in the preliminary workshops that a potential app would not be integrated with the separate information technology systems of GPs and community health centers because these systems are not interoperable. **In addition, no healthcare professionals were interested in a supplementary system that would operate in parallel to their existing technology, nor did health- care professionals feel competent or ready to use a mobile app in their consultations with patients.** This correlates with other studies finding that lack of human and technical skills are barriers for integration of health technology in practice [8, 10, 22]. This correlates with other studies finding that lack of human and technical skills are barriers for integration of health technology in practice [8, 10, 22].

Petersen M, Hempler NF. Development and testing of a mobile application to support diabetes self-management for people with newly diagnosed type 2 diabetes: a design thinking case study. *BMC Med Inform Decis Mak*. 2017;17(1):91. doi:10.1186/s12911-017-0493-6, pmid:28651639

We acknowledge that the implementation and adoption of PD technologies in health care require integration with clinical workflow [19].

Verhoeven F, Cremers A, Schoone M, Van Dijk J. Mobiles for mobility: Participatory design of a “Happy walker” that stimulates mobility among older people. *Gerontechnology*. 2016;15(1). https://journal.gerontechnology.org/currentIssueContent.aspx?aid=2248

Using the BCW method along with a UCD is comparable with the methodology used by Curtis et al [22] to develop a theory-driven and user-centered healthy eating app. Their work also focused on a thorough analysis of target behaviors, selection of BCTs, and exploration of user preferences to underpin the design of the app with relevant theory and evidence and ensure engagement among the target population. However, Curtis et al [22] performed no specific activities with regard to valorization and implementation of their app during their development process [22]. To make both the design and the implementation of our app value driven, we performed valorization activities throughout the development process of our app [59,72]. Business modeling helped us to identify critical success factors that will influence the sustainability and effectiveness of the app, which is often overlooked during the development process of eHealth and mHealth technologies [59].

Korpershoek YJG, Hermsen S, Schoonhoven L. User-centered design of a mobile health intervention to enhance exacerbation-related self-management in patients with chronic obstructive pulmonary …. *Journal of medical*. Published online 2020. https://www.jmir.org/2020/6/e15449/

Medical Device vs Consumer App

Keywords:,medical device vs consumer app, unfamiliarity, porting issues, usage vs acceptability, variance in evaluation,

Furthermore, integration of medication management into the app would have implications regarding classification of the app as a **medical device,** thus potentially limiting its accessibility via health care provider only instead of directly to the public via the consumer app store

Birnie KA, Campbell F, Nguyen C, et al. iCanCope PostOp: User-Centered Design of a Smartphone-Based App for Self-Management of Postoperative Pain in Children and Adolescents. *JMIR Form Res*. 2019;3(2):e12028. doi:10.2196/12028, pmid:31008704

Many design features commonly used in mobile apps are hitherto unknown to the COPD population, such as the sliders that were initially included in the app. Our research indicates that such design elements are not optimal for a COPD population.

Davies A, Mueller J, Hennings J, Caress A-L, Jay C. Recommendations for Developing Support Tools With People Suffering From Chronic Obstructive Pulmonary Disease: Co-Design and Pilot Testing of a Mobile Health Prototype. *JMIR Hum Factors*. 2020;7(2):e16289. doi:10.2196/16289, pmid:32410730

Approximately half of these mHealth studies tracked and reported usage data to demonstrate the level of application engagement. Epic Allies23 proposed to track application usage data (ie, number of log-ins, use of application features and application progress) as a proxy for engagement. A**llyQuest25 and PositiveLinks22 both tracked and reported usage data; however, neither specified thresholds that would be used to determine whether application use was feasible and acceptable.**

Erguera XA, Johnson MO, Neilands TB, et al. WYZ: a pilot study protocol for designing and developing a mobile health application for engagement in HIV care and medication adherence in youth and young adults living with HIV. *BMJ Open*. 2019;9(5):e030473. doi:10.1136/bmjopen-2019-030473, pmid:31061063

Through the process of codesign, we have been made aware of some of the barriers to implementing this app into face-to-face clinical care. Further work will be required to tailor implementation of the app into various service settings and governance structures. This is potentially challenging, given the different processes used across various services, and highlights that health providers and young people have different expectations and preferences with regard to the use of technology in mental health care.

Hetrick SE, Robinson J, Burge E, et al. Youth Codesign of a Mobile Phone App to Facilitate Self-Monitoring and Management of Mood Symptoms in Young People With Major Depression, Suicidal Ideation, and Self-Harm. *JMIR Ment Health*. 2018;5(1):e9. doi:10.2196/mental.9041, pmid:29362208

Depending on the topic, decisions should be made about which phases and steps are relevant to the topic and should be included in the development process. In addition, taking time aspects into consideration, it should be questioned how thoroughly an individual step should be executed.

Korpershoek YJG, Hermsen S, Schoonhoven L. User-centered design of a mobile health intervention to enhance exacerbation-related self-management in patients with chronic obstructive pulmonary .... *Journal of medical*. Published online 2020. https://www.jmir.org/2020/6/e15449/

Research and (no) Development (R&noD)

Keywords: (lack of) continuity, lack or rigour, methodological determinism, real-life environment?,

The biggest limitation of our work is the **continuity** of this project. It will be great to see the project further developed through more research to culminate into a product.

Abubakar_A_Gamifying the unspoken Designing to Resolve Myths on Contraceptives among the Himba people of Namibia.pdf. http://dx.doi.org/10.1145/3334480.XXXXXXX

Research has shown that 80% of all usability problems are detected with as few as 4-5 participants [52], and we are therefore inclined to believe that the number of participants in our study was sufficient to address the usability issues we wanted to evaluate.

Arvidsson S, Gilljam B-M, Nygren J, Ruland CM, Nordby-Bøe T, Svedberg P. Redesign and Validation of Sisom, an Interactive Assessment and Communication Tool for Children With Cancer. *JMIR Mhealth Uhealth*. 2016;4(2):e76. doi:10.2196/mhealth.5715, pmid:27343004

Allowing mothers to use the Mother's Milk Connection application after discharge home from the hospital in their natural setting considered the environment and social culture in which the application would be used in order to provide a real-life experience for valuable feedback on usability.

Jefferson UT, Zachary I, Majee W. Employing a User-Centered Design to Engage Mothers in the Development of a mHealth Breastfeeding Application. *Comput Inform Nurs*. 2019;37(10):522-531. doi:10.1097/CIN.0000000000000549, pmid:31414995

Second, we can - not be sure from the focus group data whether the RA dashboard will, once implemented, significantly change patients' health be- haviours, such as improving medication adherence, exercise or other self-management strategies, although some patients pre- dicted it might.

Liu LH, Garrett SB, Li J, et al. Patient and clinician perspectives on a patient-facing dashboard that visualizes patient reported outcomes in rheumatoid arthritis. *Health Expect*. 2020;23(4):846-859. doi:10.1111/hex.13057, pmid:32270591

Furthermore, this research is aligned to prototype development and lacks any capacity to deliver evidence on effectiveness.

Martin S, Armstrong E, Thomson E, et al. A qualitative study adopting a user-centered approach to design and validate a brain computer interface for cognitive rehabilitation for people with brain injury. *Assist Technol*. 2018;30(5):233-241. doi:10.1080/10400435.2017.1317675, pmid:28708963
