# Supplementary material for: Examining Challenges to Co-Design Digital Health Interventions With End Users: Systematic Review
Source: J Med Internet Res. 2025 Mar 14;27:e50178. doi: 10.2196/50178 (PMC11953610; doi:10.2196/50178)
Supplement: Multimedia Appendix 8 [file jmir_v27i1e50178_app8.pdf]

DIGITAL APPROACH

PRELIMINARY RESEARCH

Demographically driven
market analysis and
product comparison
largely from the
perspective of HCI, UX
and marketing.

TESTING

Rapid, iterative,
qualitative based
workshops that often
involve A/B testing and
co-design methods
largely influenced by
agile methodology. A
non-linear adaptive
process.

EFFECTIVENESS

Largely qualitative
interviews, thinkalouds
and observations
(qualitative), sometimes
combined with analytic
and usage statistics
(quantitative) to
determine user
acceptance.

END USER INVOLVEMENT
DIGITAL HEALTH INTERVENTIONS

HEALTH APPROACH

PRELIMINARY RESEARCH

Demographically driven
reviews, often
systematic or rapid that
seek to establish a
research gap for and
subsequent research
question(s).

TESTING

Hypothesis driven
quantitative research
questions that often
involve RCTs to validate
safety and effectiveness
using statistical
measures to prove or
disapprove initial
assumptions. A linear
quantifiable process.

EFFECTIVENESS

Largely quantitative
longitudinal studies that
involve cohorts of
experiment and control
groups to determine
clinical viability and
effectiveness.
